# Correction: Down-regulated GAS6 impairs synovial macrophage efferocytosis and promotes obesity-associated osteoarthritis

**DOI:** 10.7554/eLife.110769

**Published:** 2026-01-29

**Authors:** Zihao Yao, Weizhong Qi, Hongbo Zhang, Zhicheng Zhang, Liangliang Liu, Yan Shao, Hua Zeng, Jianbin Yin, Haoyan Pan, Xiongtian Guo, Anling Liu, Daozhang Cai, Xiaochun Bai, Haiyan Zhang

**Keywords:** Mouse

Yao Z, Qi W, Zhang H, Zhang Z, Liu L, Shao Y, Zeng H, Yin J, Pan H, Guo X, Liu A, Cai D, Bai X, Zhang H. 2023. Down-regulated GAS6 impairs synovial macrophage efferocytosis and promotes obesity-associated osteoarthritis. *eLife* **12**:e83069. doi: 10.7554/eLife.83069.

Published 5 May 2023

During an internal audit, we identified three separate instances of unintended image duplication in the published figures. The errors and their causes are described below.

**1) In Figure 4—figure supplement 2 (Panel A)**

The panel labeled “LPS” was inadvertently duplicated from the Apoe⁻/⁻ group shown in Figure 4E, and the panel labeled “LPS +rmGAS6+R428” was reused from the R428-treated group in Figure 4H.

This occurred because raw TIFF files with nearly identical prefixes (“Effero_”) and the suffix “_400 X” were stored together in a central folder (“Figure 4>Efferocytosis”). Without checking the experimental records, the wrong image was accidentally selected during the assembly of the early draft version of the figure. The affected panels have been replaced with the correct original images, and all raw data are provided for reference.

**2) In Figure 4—figure supplement 1A and Figure 3A**

The panel intended to represent “Obese OA individuals” was mistakenly replaced with a fluorescence-staining image previously used for “Normal OA” in Figure 3A.

The two duplicate images were derived from different fields of view of the same synovial tissue stained with F4/80-GAS6 fluorescence. Our laboratory routinely captures multiple fields to document representative pathological features. The F4/80-MER fluorescent TIFF files (IF_Homo_Ob_OA.tif) in Figure 4—figure supplement shares the same “IF_Homo_” prefix as the F4/80-GAS6 files (IF_Homo_Nor_OA.tif) in Figure 3. During the revision process, while assembling the image panels for Figure 4—figure supplement 1A, we inadvertently selected the F4/80-GAS6 (IF_Homo_Nor_OA.tif) image from the adjacent Figure 3 folder. The “Obese OA” panel in Figure 4—figure supplement 1A was horizontally flipped to center the synovial villi structure in the figure. This unfortunate operational error resulted in partially overlapping and reversed regions between the two panels. We have corrected this by replacing the erroneous panel with the appropriate original image and have provided the original datasets for review.

**3) In Figure 2—figure supplement 2A**

The panel labeled “Apoe⁻/⁻” for DAPI/CD206 staining was mistakenly replaced with an image from the “C57BL/6” group.

These two images were taken from different fields of view of the synovial tissue in the sham Control C57BL/6 group. The TIFF files “IF_Mus_SA_11” and “IF_Mus_SC_2” were stored in adjacent folders and shared the same “IF_Mus” prefix, with only slight differences in the suffixes “SA” and “SC”. In thumbnail view, they appeared almost identical, which led to the wrong image being selected during assembly. As a result, both panels in the figure came from the C57BL/6 group, creating unintended overlap. We have replaced the image panel from the original data and provided all original data images for reference.

These errors arose primarily from storing related TIFF files in adjacent folders without sufficiently distinct naming conventions, coupled with the absence of a second-person verification step during revision.

All quantitative analyses in the manuscript were performed using the correct original images. Therefore, the numerical data, statistical outcomes, and overall conclusions of the paper remain valid.

**Correction #1:**

The corrected Figure 4—figure supplement 2 (with panel A updated) is shown here:

**Figure 1:**
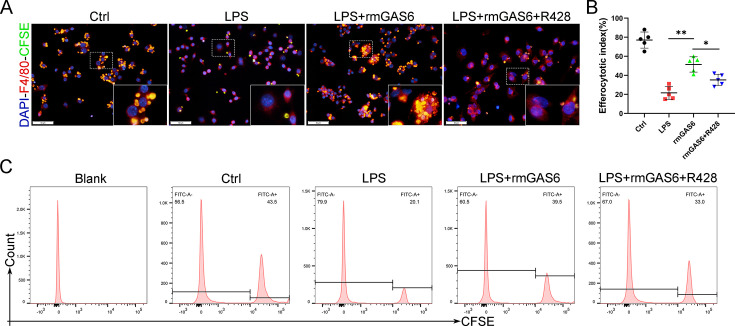


The originally published Figure 4—figure supplement 2 is shown for reference:

**Figure 2:**
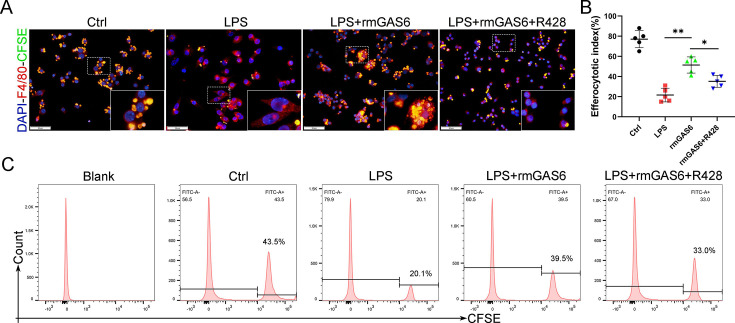


The legend associated with Figure 4—figure supplement 2 is shown here:

**Figure 4—figure supplement 2. rmGAS6 attenuated the impaired efferocytosis induced by LPS.**

Immunofluorescence staining for F4/80 (red) in bone marrow-derived macrophage (BMDM) cells and carboxyfluorescein succinimidyl ester (CFSE; green) in apoptotic thymocytes after phagocytosis for 2 hr. Scale bar: 50 µm. (B) Quantification of positive BMDM cells engulfing apoptotic thymocytes as a proportion of total F4/80-positive cells, n=5 per group. (C) Flow cytometry analysis of CFSE-positive cells in total macrophages is shown as fluorescence‐intensity distribution plots. *P<0.05, **P<0.01, NS = not significant. One-way analysis of variance (ANOVA) was performed. Data are shown as mean ± standard deviation (SD).

For context, we also show the panels from Figure 4E-H; the Apoe⁻/⁻ and R428 panels from this figure were duplicated in Figure 4—figure supplement 2A:

**Figure 3:**
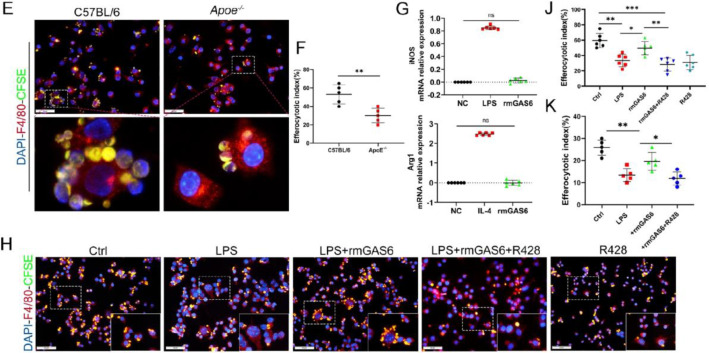


**Correction #2:**

The corrected Figure 4—figure supplement 1 (with panel A updated) is shown here:

**Figure 4:**
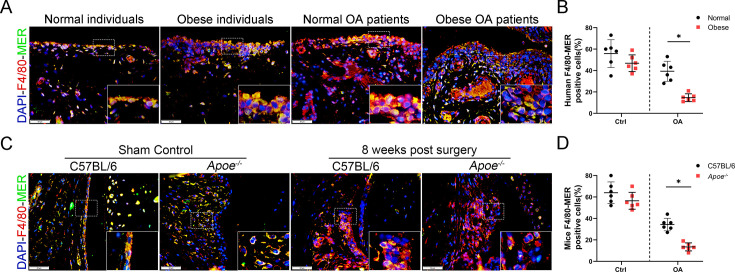


The originally published Figure 4—figure supplement 1 is shown for reference:

**Figure 5:**
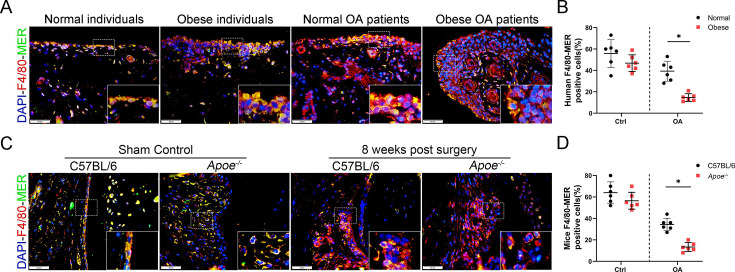


The legend associated with Figure 4—figure supplement 1 is shown here:

**Figure 4—figure supplement 1. The expression of MER decreased in obese OA patients and Apoe−/− OA mice**. (A) Immunofluorescence staining for F4/80 (red) and MER (green) in synovial tissue from normal individuals; osteoarthritis (OA) patients without obesity; obese individuals, and OA patients with obesity. Scale bar: 50 μm. (B) Quantification of F4/80-MER-positive macrophages as a proportion of total F4/80-positive lining cell population in (A), n=6 per group. (C) Immunofluorescence staining for F4/80 (red) and MER (green) in synovial issue of controls and destabilization of medial meniscus (DMM) from C57BL/6 and Apoe−/− mice. Scale bar: 50 μm. (D) Quantification of F4/80-MER- positive macrophages (yellow) as a proportion of total F4/80-positive cells in (C). n=6 per group. *P<0.05. One-way analysis of variance (ANOVA) was performed. Data are shown as mean ± standard deviation (SD).

For context, we also show the panels from Figure 3A; the “Normal OA patients” image was erroneously reused (the image was reversed and overlaps) to represent “Obese OA individuals” in Figure 4—figure supplement 1A:

**Figure 6:**
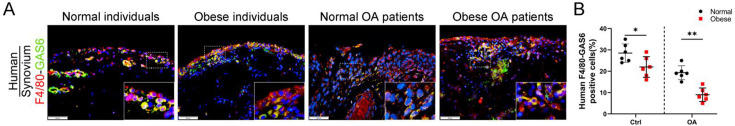


**Correction #3:**

The corrected Figure 2—figure supplement 2 (with panel A updated) is shown here:

**Figure 7:**
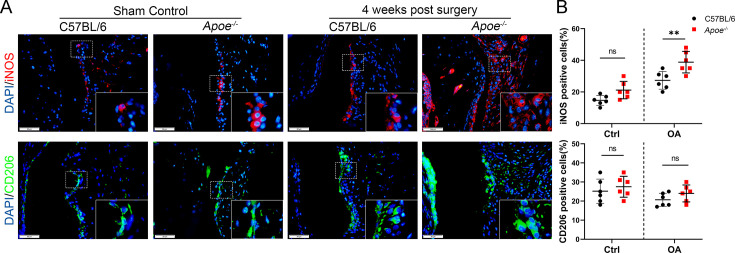


The originally published Figure 2—figure supplement 2 is shown for reference:

**Figure 8:**
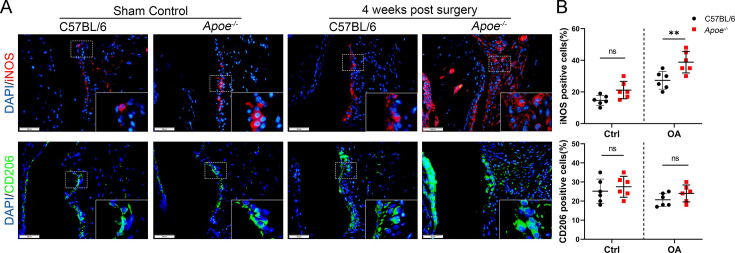


The legend associated with Figure 2—figure supplement 2 is shown here:

**Figure 2—figure supplement 2. Macrophage polarization in 4 week-old Apoe−/− OA mice**. (A) Immunofluorescence of inducible nitric oxide synthase (iNOS) and CD206 in controls and destabilization of medial meniscus (DMM) synovial tissues from normal and Apoe−/− mice 4 weeks after surgery. Scale bar: 50 μm. (B) Quantification of iNOS- and CD206-positive cells as a proportion of lining cells in (A). n=6 per group. **P<0.01, ns = not significant. One-way analysis of variance (ANOVA) was performed. Data are shown as mean ± standard deviation (SD).

The article has been corrected accordingly.

