# Supplementary material for: Down-regulated GAS6 impairs synovial macrophage efferocytosis and promotes obesity-associated osteoarthritis
Source: eLife. 2023 May 5;12:e83069. doi: 10.7554/eLife.83069 (PMC10191622; doi:10.7554/eLife.83069)
Supplement: Figure 3—figure supplement 1—source data 1. [file elife-83069-fig3-figsupp1-data1.zip › Figure 3- figure supplement 1-souce data 1.docx]

**Figure 3- figure supplement 2.** Results of GSE53986

| ID | adj.P.Val | P.Value | t | B | logFC | Gene.symbol | Gene.title | GO.Function | GO.Process | GO.Component |
| --- | --- | --- | --- | --- | --- | --- | --- | --- | --- | --- |
| 1425663_at | 0.000113 | 1.39E-08 | 2.61E+01 | 9.791954 | 6.41 | Il1rn | interleukin 1 receptor antagonist | cytokine activity///interleukin-1 Type I receptor antagonist activity///interleukin-1 Type II receptor antagonist activity///interleukin-1 receptor antagonist activity///interleukin-1 receptor binding///interleukin-1 receptor binding///interleukin-1, Type I receptor binding///interleukin-1, Type II receptor binding | cytokine-mediated signaling pathway///fever generation///insulin secretion///lipid metabolic process///memory///negative regulation of apoptotic process///negative regulation of cell migration///negative regulation of glutamate secretion///negative regulation of heterotypic cell-cell adhesion///negative regulation of interleukin-1-mediated signaling pathway///negative regulation of membrane potential///positive regulation of JUN kinase activity///response to glucocorticoid///sensory perception of pain | cytoplasm///extracellular exosome///extracellular region///extracellular space///extracellular space///vesicle |
| 1418930_at | 0.000113 | 1.47E-08 | 2.59E+01 | 9.755588 | 7.1 | Cxcl10 | chemokine (C-X-C motif) ligand 10 | CXCR chemokine receptor binding///CXCR3 chemokine receptor binding///chemokine activity///chemokine activity///cytokine activity///heparin binding | G-protein coupled receptor signaling pathway///G-protein coupled receptor signaling pathway///T cell chemotaxis///cellular response to lipopolysaccharide///chemokine-mediated signaling pathway///chemokine-mediated signaling pathway///chemotaxis///defense response to virus///endothelial cell activation///immune response///immune response///inflammatory response///negative regulation of angiogenesis///negative regulation of myoblast differentiation///negative regulation of myoblast fusion///positive regulation of T cell migration///positive regulation of cAMP metabolic process///positive regulation of cAMP-mediated signaling///positive regulation of cell migration///positive regulation of cell proliferation///positive regulation of leukocyte chemotaxis///positive regulation of leukocyte chemotaxis///positive regulation of monocyte chemotaxis///positive regulation of release of sequestered calcium ion into cytosol///regulation of T cell chemotaxis///regulation of cell proliferation///regulation of cell proliferation///regulation of endothelial tube morphogenesis///response to lipopolysaccharide | external side of plasma membrane///extracellular region///extracellular space///extracellular space |
| 1423017_a_at | 0.000113 | 1.47E-08 | 2.59E+01 | 9.75277 | 6.24 | Il1rn | interleukin 1 receptor antagonist | cytokine activity///interleukin-1 Type I receptor antagonist activity///interleukin-1 Type II receptor antagonist activity///interleukin-1 receptor antagonist activity///interleukin-1 receptor binding///interleukin-1 receptor binding///interleukin-1, Type I receptor binding///interleukin-1, Type II receptor binding | cytokine-mediated signaling pathway///fever generation///insulin secretion///lipid metabolic process///memory///negative regulation of apoptotic process///negative regulation of cell migration///negative regulation of glutamate secretion///negative regulation of heterotypic cell-cell adhesion///negative regulation of interleukin-1-mediated signaling pathway///negative regulation of membrane potential///positive regulation of JUN kinase activity///response to glucocorticoid///sensory perception of pain | cytoplasm///extracellular exosome///extracellular region///extracellular space///extracellular space///vesicle |
| 1418240_at | 0.000113 | 1.56E-08 | 2.56E+01 | 9.71288 | 5.22 | Gbp2 | guanylate binding protein 2 | GTP binding///GTPase activity///hydrolase activity///nucleotide binding | adhesion of symbiont to host///cellular response to interferon-beta///cellular response to interferon-gamma///cellular response to lipopolysaccharide///defense response to Gram-positive bacterium///defense response to protozoan | Golgi apparatus///cytoplasm///cytoplasmic vesicle///membrane///symbiont-containing vacuole membrane |
| 1434372_at | 0.000113 | 1.62E-08 | 2.55E+01 | 9.689475 | 5.36 | AW112010 | expressed sequence AW112010 | molecular_function | biological_process | cellular_component |
| 1435529_at | 0.000113 | 1.65E-08 | 2.55E+01 | 9.679083 | 7.25 | Ifit1bl1 | interferon induced protein with tetratricpeptide repeats 1B like 1 | RNA binding///RNA binding | defense response to virus | cytoplasm |
| 1435906_x_at | 0.000113 | 1.80E-08 | 2.51E+01 | 9.617299 | 4.9 | Gbp2 | guanylate binding protein 2 | GTP binding///GTPase activity///hydrolase activity///nucleotide binding | adhesion of symbiont to host///cellular response to interferon-beta///cellular response to interferon-gamma///cellular response to lipopolysaccharide///defense response to Gram-positive bacterium///defense response to protozoan | Golgi apparatus///cytoplasm///cytoplasmic vesicle///membrane///symbiont-containing vacuole membrane |
| 1425108_a_at | 0.000113 | 2.00E-08 | -2.48E+01 | 9.54737 | -3.72 | Smagp | small cell adhesion glycoprotein | molecular_function | biological_process | cell junction///cytoplasmic vesicle///integral component of membrane///membrane///nucleoplasm///plasma membrane |
| 1419714_at | 0.000121 | 2.88E-08 | 2.36E+01 | 9.294407 | 3.88 | Cd274 | CD274 antigen | protein binding | T cell costimulation///cell surface receptor signaling pathway///immune response///negative regulation of T cell proliferation///negative regulation of activated T cell proliferation///negative regulation of interferon-gamma production///negative regulation of interleukin-10 production///negative regulation of tumor necrosis factor superfamily cytokine production///positive regulation of T cell proliferation///positive regulation of cell migration///positive regulation of interleukin-10 secretion///response to cytokine///signal transduction///toxin transport | cell surface///external side of plasma membrane///extracellular exosome///integral component of membrane///membrane///plasma membrane |
| 1420928_at | 0.000121 | 3.31E-08 | -2.32E+01 | 9.19543 | -4.9 | St6gal1 | beta galactoside alpha 2,6 sialyltransferase 1 | beta-galactoside alpha-2,6-sialyltransferase activity///beta-galactoside alpha-2,6-sialyltransferase activity///sialyltransferase activity///sialyltransferase activity///transferase activity///transferase activity, transferring glycosyl groups | N-acetylneuraminate metabolic process///negative regulation of chemotaxis///negative regulation of macrophage apoptotic process///oligosaccharide metabolic process///positive regulation of mononuclear cell proliferation///protein N-linked glycosylation via asparagine///protein N-linked glycosylation via asparagine///protein glycosylation///protein sialylation///regulation of substrate adhesion-dependent cell spreading///sialylation | Golgi apparatus///Golgi medial cisterna///Golgi trans cisterna///endoplasmic reticulum///extracellular exosome///extracellular region///integral component of Golgi membrane///integral component of membrane///membrane |
| 1451798_at | 0.000121 | 3.90E-08 | 2.26E+01 | 9.077469 | 6.02 | Il1rn | interleukin 1 receptor antagonist | cytokine activity///interleukin-1 Type I receptor antagonist activity///interleukin-1 Type II receptor antagonist activity///interleukin-1 receptor antagonist activity///interleukin-1 receptor binding///interleukin-1 receptor binding///interleukin-1, Type I receptor binding///interleukin-1, Type II receptor binding | cytokine-mediated signaling pathway///fever generation///insulin secretion///lipid metabolic process///memory///negative regulation of apoptotic process///negative regulation of cell migration///negative regulation of glutamate secretion///negative regulation of heterotypic cell-cell adhesion///negative regulation of interleukin-1-mediated signaling pathway///negative regulation of membrane potential///positive regulation of JUN kinase activity///response to glucocorticoid///sensory perception of pain | cytoplasm///extracellular exosome///extracellular region///extracellular space///extracellular space///vesicle |
| 1434046_at | 0.000121 | 4.09E-08 | 2.25E+01 | 9.043219 | 5.28 | AA467197 | expressed sequence AA467197 | NADH dehydrogenase (ubiquinone) activity///contributes_to cytochrome-c oxidase activity | biological_process | mitochondrial respiratory chain complex IV///mitochondrion///nucleus |
| 1434380_at | 0.000121 | 4.18E-08 | 2.24E+01 | 9.026879 | 4.24 | Gbp7 | guanylate binding protein 7 | molecular_function | adhesion of symbiont to host///cellular response to interferon-gamma///defense response to Gram-positive bacterium///defense response to protozoan///defense response to protozoan | cytoplasmic vesicle///symbiont-containing vacuole membrane |
| 1418652_at | 0.000121 | 4.20E-08 | 2.24E+01 | 9.023587 | 1.02E+01 | Cxcl9 | chemokine (C-X-C motif) ligand 9 | CXCR chemokine receptor binding///chemokine activity///cytokine activity | G-protein coupled receptor signaling pathway///chemokine-mediated signaling pathway///chemotaxis///defense response to virus///immune response///inflammatory response///positive regulation of leukocyte chemotaxis///positive regulation of myoblast differentiation///positive regulation of myoblast fusion///regulation of cell proliferation///response to lipopolysaccharide | external side of plasma membrane///extracellular region///extracellular space |
| 1450430_at | 0.000121 | 4.27E-08 | -2.24E+01 | 9.011531 | -4.64 | Mrc1 | mannose receptor, C type 1 | carbohydrate binding///mannose binding///receptor activity///transmembrane signaling receptor activity | cellular response to interferon-gamma///cellular response to interleukin-4///cellular response to lipopolysaccharide///endocytosis///receptor-mediated endocytosis | cell surface///endosome///endosome membrane///integral component of membrane///membrane///plasma membrane |
| 1452458_s_at | 0.000121 | 4.29E-08 | -2.24E+01 | 9.007773 | -4.99 | Lrr1 | leucine rich repeat protein 1 | molecular_function | biological_process | cellular_component |
| 1450484_a_at | 0.000142 | 5.34E-08 | 2.17E+01 | 8.845412 | 5.37 | Cmpk2 | cytidine monophosphate (UMP-CMP) kinase 2, mitochondrial | ATP binding///UMP kinase activity///cytidylate kinase activity///kinase activity///nucleoside diphosphate kinase activity///nucleotide binding///thymidylate kinase activity///thymidylate kinase activity///transferase activity///uridylate kinase activity///uridylate kinase activity | cellular response to lipopolysaccharide///dTDP biosynthetic process///dTDP biosynthetic process///dTTP biosynthetic process///dUDP biosynthetic process///dUDP biosynthetic process///nucleoside diphosphate phosphorylation///nucleoside triphosphate biosynthetic process///phosphorylation///pyrimidine nucleotide biosynthetic process | cytosol///mitochondrion///mitochondrion///nucleoplasm///nucleus |
| 1433719_at | 0.00016 | 6.39E-08 | -2.12E+01 | 8.709028 | -4.47 | Slc9a9 | solute carrier family 9 (sodium/hydrogen exchanger), member 9 | antiporter activity///potassium:proton antiporter activity///sodium:proton antiporter activity///solute:proton antiporter activity | cation transport///ion transport///potassium ion transmembrane transport///regulation of intracellular pH///regulation of pH///sodium ion import across plasma membrane///sodium ion transport///transmembrane transport///transport | endosome///integral component of membrane///membrane///plasma membrane///recycling endosome///recycling endosome |
| 1438037_at | 0.000164 | 6.92E-08 | 2.09E+01 | 8.649053 | 3.97 | Herc6 | hect domain and RLD 6 | UDP-N-acetylmuramoylalanyl-D-glutamyl-2,6-diaminopimelate-D-alanyl-D-alanine ligase activity///coenzyme F420-0 gamma-glutamyl ligase activity///coenzyme F420-2 alpha-glutamyl ligase activity///cyclin binding///ligase activity///protein-glutamic acid ligase activity///protein-glycine ligase activity///protein-glycine ligase activity, elongating///protein-glycine ligase activity, initiating///ribosomal S6-glutamic acid ligase activity///tubulin-glutamic acid ligase activity///tubulin-glycine ligase activity///ubiquitin protein ligase activity///ubiquitin-protein transferase activity///ubiquitin-protein transferase activity | hematopoietic progenitor cell differentiation///immune system process///innate immune response///protein ubiquitination///protein ubiquitination involved in ubiquitin-dependent protein catabolic process | cytoplasm///cytoplasm///nucleus///nucleus |
| 1435389_at | 0.000165 | 7.32E-08 | -2.08E+01 | 8.605277 | -3.43 | Reps2 | RALBP1 associated Eps domain containing protein 2 | calcium ion binding///metal ion binding///molecular_function | biological_process | cellular_component///cytoplasm |
| 1460431_at | 0.000171 | 8.23E-08 | -2.05E+01 | 8.51488 | -2.94 | Gcnt1 | glucosaminyl (N-acetyl) transferase 1, core 2 | acetylglucosaminyltransferase activity///beta-1,3-galactosyl-O-glycosyl-glycoprotein beta-1,6-N-acetylglucosaminyltransferase activity///transferase activity///transferase activity, transferring glycosyl groups | cell adhesion molecule production///glycoprotein biosynthetic process///kidney morphogenesis///leukocyte tethering or rolling///tissue morphogenesis | Golgi apparatus///Golgi cisterna///extracellular space///integral component of membrane///membrane///trans-Golgi network |
| 1448627_s_at | 0.000171 | 8.52E-08 | -2.04E+01 | 8.487302 | -3.63 | Pbk | PDZ binding kinase | ATP binding///kinase activity///nucleotide binding///protein kinase activity///protein serine/threonine kinase activity///protein serine/threonine kinase activity///transferase activity | cellular response to UV///negative regulation of inflammatory response///negative regulation of proteasomal ubiquitin-dependent protein catabolic process///negative regulation of protein phosphorylation///negative regulation of stress-activated MAPK cascade///peptidyl-serine phosphorylation///peptidyl-threonine phosphorylation///phosphorylation///protein phosphorylation | nucleus |
| 1433711_s_at | 0.000171 | 9.05E-08 | -2.02E+01 | 8.440922 | -3.2 | Sesn1 | sestrin 1 | leucine binding///oxidoreductase activity///peroxiredoxin activity///protein binding | cellular oxidant detoxification///cellular response to amino acid stimulus///cellular response to amino acid stimulus///negative regulation of TORC1 signaling///negative regulation of TORC1 signaling///negative regulation of cell growth///oxidation-reduction process///reactive oxygen species metabolic process///regulation of protein kinase B signaling///regulation of response to reactive oxygen species | colocalizes_with GATOR2 complex///cytoplasm///nucleus |
| 1448944_at | 0.000171 | 9.07E-08 | -2.02E+01 | 8.438408 | -2.83 | Nrp1 | neuropilin 1 | growth factor binding///growth factor binding///heparin binding///metal ion binding///protein binding///semaphorin receptor activity///semaphorin receptor activity///semaphorin receptor activity///semaphorin receptor activity///vascular endothelial growth factor binding///vascular endothelial growth factor binding///vascular endothelial growth factor-activated receptor activity///vascular endothelial growth factor-activated receptor activity | VEGF-activated neuropilin signaling pathway///VEGF-activated neuropilin signaling pathway involved in axon guidance///angiogenesis///angiogenesis///angiogenesis///angiogenesis involved in coronary vascular morphogenesis///artery morphogenesis///axon extension involved in axon guidance///axon guidance///axon guidance///axon guidance///axonal fasciculation///axonogenesis involved in innervation///branchiomotor neuron axon guidance///cell differentiation///cell migration///cell migration involved in coronary vasculogenesis///cell migration involved in sprouting angiogenesis///cell migration involved in sprouting angiogenesis///cellular response to hepatocyte growth factor stimulus///cellular response to vascular endothelial growth factor stimulus///commissural neuron axon guidance///coronary artery morphogenesis///dendrite development///dichotomous subdivision of terminal units involved in salivary gland branching///dichotomous subdivision of terminal units involved in salivary gland branching///dorsal root ganglion morphogenesis///endothelial cell chemotaxis///facial nerve structural organization///facioacoustic ganglion development///gonadotrophin-releasing hormone neuronal migration to the hypothalamus///heart development///hepatocyte growth factor receptor signaling pathway///motor neuron axon guidance///multicellular organism development///negative regulation of axon extension///negative regulation of axon extension involved in axon guidance///negative regulation of axon extension involved in axon guidance///negative regulation of extrinsic apoptotic signaling pathway///negative regulation of neuron apoptotic process///nerve development///nervous system development///neural crest cell migration involved in autonomic nervous system development///neuron development///neuron migration///organ morphogenesis///otic placode development///patterning of blood vessels///platelet-derived growth factor receptor signaling pathway///positive chemotaxis///positive regulation of ERK1 and ERK2 cascade///positive regulation of axon extension involved in axon guidance///positive regulation of peptidyl-tyrosine phosphorylation///positive regulation of peptidyl-tyrosine phosphorylation///positive regulation of retinal ganglion cell axon guidance///protein localization to early endosome///regulation of axon extension involved in axon guidance///regulation of retinal ganglion cell axon guidance///renal artery morphogenesis///retina vasculature morphogenesis in camera-type eye///retinal ganglion cell axon guidance///semaphorin-plexin signaling pathway///semaphorin-plexin signaling pathway///semaphorin-plexin signaling pathway involved in axon guidance///semaphorin-plexin signaling pathway involved in neuron projection guidance///sensory neuron axon guidance///sprouting angiogenesis///sympathetic ganglion development///sympathetic nervous system development///sympathetic neuron projection extension///sympathetic neuron projection guidance///toxin transport///trigeminal ganglion development///trigeminal nerve morphogenesis///trigeminal nerve structural organization///vascular endothelial growth factor receptor signaling pathway///vascular endothelial growth factor receptor signaling pathway///vascular endothelial growth factor signaling pathway///ventral trunk neural crest cell migration///vestibulocochlear nerve structural organization | axon///axon///cell surface///cytosol///early endosome///extracellular space///focal adhesion///growth cone///integral component of membrane///membrane///neurofilament///neuronal cell body///plasma membrane///plasma membrane///sorting endosome |
| 1424339_at | 0.000172 | 9.62E-08 | 2.00E+01 | 8.392732 | 6.11 | Oasl1 | 2'-5' oligoadenylate synthetase-like 1 | NOT 2'-5'-oligoadenylate synthetase activity///ATP binding///DNA binding///RNA binding///double-stranded RNA binding///double-stranded RNA binding///poly(A) RNA binding///transferase activity | defense response to virus///immune response///immune system process///innate immune response///negative regulation of viral genome replication///response to virus | cytoplasm///membrane///nucleolus///nucleus |
| 1418392_a_at | 0.000172 | 1.01E-07 | 1.99E+01 | 8.35602 | 4.58 | Gbp3 | guanylate binding protein 3 | GTP binding///GTPase activity///hydrolase activity///nucleotide binding | adhesion of symbiont to host///cellular response to interferon-beta///cellular response to interferon-gamma///defense response to Gram-positive bacterium///defense response to protozoan | Golgi apparatus///cytoplasm///cytoplasmic vesicle///cytosol///membrane///nucleus///symbiont-containing vacuole membrane |
| 1442787_at | 0.000172 | 1.10E-07 | -1.97E+01 | 8.288104 | -3.82 |  |  |  |  |  |
| 1418612_at | 0.000172 | 1.10E-07 | 1.96E+01 | 8.282691 | 6.65 | Slfn1 | schlafen 1 | protein binding | cell cycle arrest///negative regulation of G1/S transition of mitotic cell cycle///negative regulation of G1/S transition of mitotic cell cycle///negative regulation of G1/S transition of mitotic cell cycle by negative regulation of transcription from RNA polymerase II promoter///negative regulation of T cell proliferation///negative regulation of cell proliferation///negative regulation of protein phosphorylation///negative regulation of transcription from RNA polymerase II promoter | cytoplasm///nucleus |
| 1425156_at | 0.000172 | 1.11E-07 | 1.96E+01 | 8.280693 | 4.39 | Gbp7 | guanylate binding protein 7 | molecular_function | adhesion of symbiont to host///cellular response to interferon-gamma///defense response to Gram-positive bacterium///defense response to protozoan///defense response to protozoan | cytoplasmic vesicle///symbiont-containing vacuole membrane |
| 1454699_at | 0.000174 | 1.16E-07 | -1.95E+01 | 8.245033 | -3.47 | Sesn1 | sestrin 1 | leucine binding///oxidoreductase activity///peroxiredoxin activity///protein binding | cellular oxidant detoxification///cellular response to amino acid stimulus///cellular response to amino acid stimulus///negative regulation of TORC1 signaling///negative regulation of TORC1 signaling///negative regulation of cell growth///oxidation-reduction process///reactive oxygen species metabolic process///regulation of protein kinase B signaling///regulation of response to reactive oxygen species | colocalizes_with GATOR2 complex///cytoplasm///nucleus |
| 1418084_at | 0.0002 | 1.42E-07 | -1.90E+01 | 8.082121 | -3.01 | Nrp1 | neuropilin 1 | growth factor binding///growth factor binding///heparin binding///metal ion binding///protein binding///semaphorin receptor activity///semaphorin receptor activity///semaphorin receptor activity///semaphorin receptor activity///vascular endothelial growth factor binding///vascular endothelial growth factor binding///vascular endothelial growth factor-activated receptor activity///vascular endothelial growth factor-activated receptor activity | VEGF-activated neuropilin signaling pathway///VEGF-activated neuropilin signaling pathway involved in axon guidance///angiogenesis///angiogenesis///angiogenesis///angiogenesis involved in coronary vascular morphogenesis///artery morphogenesis///axon extension involved in axon guidance///axon guidance///axon guidance///axon guidance///axonal fasciculation///axonogenesis involved in innervation///branchiomotor neuron axon guidance///cell differentiation///cell migration///cell migration involved in coronary vasculogenesis///cell migration involved in sprouting angiogenesis///cell migration involved in sprouting angiogenesis///cellular response to hepatocyte growth factor stimulus///cellular response to vascular endothelial growth factor stimulus///commissural neuron axon guidance///coronary artery morphogenesis///dendrite development///dichotomous subdivision of terminal units involved in salivary gland branching///dichotomous subdivision of terminal units involved in salivary gland branching///dorsal root ganglion morphogenesis///endothelial cell chemotaxis///facial nerve structural organization///facioacoustic ganglion development///gonadotrophin-releasing hormone neuronal migration to the hypothalamus///heart development///hepatocyte growth factor receptor signaling pathway///motor neuron axon guidance///multicellular organism development///negative regulation of axon extension///negative regulation of axon extension involved in axon guidance///negative regulation of axon extension involved in axon guidance///negative regulation of extrinsic apoptotic signaling pathway///negative regulation of neuron apoptotic process///nerve development///nervous system development///neural crest cell migration involved in autonomic nervous system development///neuron development///neuron migration///organ morphogenesis///otic placode development///patterning of blood vessels///platelet-derived growth factor receptor signaling pathway///positive chemotaxis///positive regulation of ERK1 and ERK2 cascade///positive regulation of axon extension involved in axon guidance///positive regulation of peptidyl-tyrosine phosphorylation///positive regulation of peptidyl-tyrosine phosphorylation///positive regulation of retinal ganglion cell axon guidance///protein localization to early endosome///regulation of axon extension involved in axon guidance///regulation of retinal ganglion cell axon guidance///renal artery morphogenesis///retina vasculature morphogenesis in camera-type eye///retinal ganglion cell axon guidance///semaphorin-plexin signaling pathway///semaphorin-plexin signaling pathway///semaphorin-plexin signaling pathway involved in axon guidance///semaphorin-plexin signaling pathway involved in neuron projection guidance///sensory neuron axon guidance///sprouting angiogenesis///sympathetic ganglion development///sympathetic nervous system development///sympathetic neuron projection extension///sympathetic neuron projection guidance///toxin transport///trigeminal ganglion development///trigeminal nerve morphogenesis///trigeminal nerve structural organization///vascular endothelial growth factor receptor signaling pathway///vascular endothelial growth factor receptor signaling pathway///vascular endothelial growth factor signaling pathway///ventral trunk neural crest cell migration///vestibulocochlear nerve structural organization | axon///axon///cell surface///cytosol///early endosome///extracellular space///focal adhesion///growth cone///integral component of membrane///membrane///neurofilament///neuronal cell body///plasma membrane///plasma membrane///sorting endosome |
| 1457999_at | 0.0002 | 1.46E-07 | -1.89E+01 | 8.05823 | -4.05 | Rmdn1 | regulator of microtubule dynamics 1 | molecular_function | biological_process | cytoplasm///cytoskeleton///microtubule///mitochondrion |
| 1422397_a_at | 0.0002 | 1.46E-07 | 1.89E+01 | 8.055618 | 3.61 | Il15ra | interleukin 15 receptor, alpha chain | | JAK-STAT cascade///negative regulation of neuron projection development///positive regulation of natural killer cell differentiation | cytoplasmic vesicle///extracellular region///integral component of membrane///membrane///nucleus///plasma membrane |
| 1421009_at | 0.000204 | 1.57E-07 | 1.87E+01 | 7.996584 | 5.45 | Rsad2 | radical S-adenosyl methionine domain containing 2 | 4 iron, 4 sulfur cluster binding///catalytic activity///iron-sulfur cluster binding///metal ion binding///protein binding///protein self-association | CD4-positive, alpha-beta T cell activation///CD4-positive, alpha-beta T cell differentiation///defense response to virus///defense response to virus///immune system process///innate immune response///negative regulation of protein secretion///negative regulation of viral genome replication///negative regulation of viral genome replication///ossification///positive regulation of T-helper 2 cell cytokine production///positive regulation of toll-like receptor 7 signaling pathway///positive regulation of toll-like receptor 9 signaling pathway///regulation of ossification///response to virus///response to virus | endoplasmic reticulum///endoplasmic reticulum///endoplasmic reticulum membrane///lipid particle///membrane///mitochondrial inner membrane///mitochondrial outer membrane///mitochondrion |
| 1427102_at | 0.000204 | 1.59E-07 | 1.87E+01 | 7.990559 | 7.61 | Slfn4 | schlafen 4 | molecular_function | biological_process | cellular_component |
| 1449450_at | 0.000272 | 2.17E-07 | 1.79E+01 | 7.729814 | 6.36 | Ptges | prostaglandin E synthase | glutathione binding///isomerase activity///prostaglandin-D synthase activity///prostaglandin-E synthase activity | fatty acid biosynthetic process///fatty acid metabolic process///lipid metabolic process///negative regulation of cell proliferation///prostaglandin biosynthetic process///prostaglandin metabolic process | cytoplasm///integral component of membrane///intracellular membrane-bounded organelle///membrane///nuclear envelope lumen///NOT nucleus///perinuclear region of cytoplasm |
| 1433454_at | 0.000298 | 2.45E-07 | 1.76E+01 | 7.629643 | 6.14 | Abtb2 | ankyrin repeat and BTB (POZ) domain containing 2 | protein heterodimerization activity///ubiquitin protein ligase binding | cellular response to toxic substance///proteasome-mediated ubiquitin-dependent protein catabolic process///protein ubiquitination involved in ubiquitin-dependent protein catabolic process///regulation of proteolysis | SCF ubiquitin ligase complex///cytoplasm///nucleoplasm///nucleus///nucleus |
| 1423883_at | 0.000303 | 2.55E-07 | 1.75E+01 | 7.593725 | 3.51 | Acsl1 | acyl-CoA synthetase long-chain family member 1 | ATP binding///acetate-CoA ligase (ADP-forming) activity///catalytic activity///decanoate--CoA ligase activity///ligase activity///long-chain fatty acid-CoA ligase activity///nucleotide binding | adiponectin-activated signaling pathway///fatty acid metabolic process///fatty acid transport///lipid biosynthetic process///lipid metabolic process///long-chain fatty acid import///long-chain fatty acid metabolic process///metabolic process///positive regulation of protein serine/threonine kinase activity///response to organic substance///triglyceride metabolic process///xenobiotic catabolic process | endoplasmic reticulum///integral component of membrane///intracellular membrane-bounded organelle///membrane///mitochondrial outer membrane///mitochondrion///mitochondrion///peroxisomal membrane///peroxisome///plasma membrane |
| 1449331_a_at | 0.000306 | 2.67E-07 | -1.74E+01 | 7.556202 | -5.13 | Rapsn | receptor-associated protein of the synapse | acetylcholine receptor binding///acetylcholine receptor binding///ionotropic glutamate receptor binding///metal ion binding///protein anchor///zinc ion binding | chemical synaptic transmission///positive regulation of neuromuscular synaptic transmission///positive regulation of neuron apoptotic process///regulation of postsynaptic membrane organization///skeletal muscle acetylcholine-gated channel clustering///synaptic transmission, cholinergic | Golgi apparatus///cell junction///cytoplasm///cytoskeleton///membrane///neuromuscular junction///neuromuscular junction///plasma membrane///postsynaptic membrane///synapse |
| 1448943_at | 0.000306 | 2.86E-07 | -1.72E+01 | 7.49888 | -2.72 | Nrp1 | neuropilin 1 | growth factor binding///growth factor binding///heparin binding///metal ion binding///protein binding///semaphorin receptor activity///semaphorin receptor activity///semaphorin receptor activity///semaphorin receptor activity///vascular endothelial growth factor binding///vascular endothelial growth factor binding///vascular endothelial growth factor-activated receptor activity///vascular endothelial growth factor-activated receptor activity | VEGF-activated neuropilin signaling pathway///VEGF-activated neuropilin signaling pathway involved in axon guidance///angiogenesis///angiogenesis///angiogenesis///angiogenesis involved in coronary vascular morphogenesis///artery morphogenesis///axon extension involved in axon guidance///axon guidance///axon guidance///axon guidance///axonal fasciculation///axonogenesis involved in innervation///branchiomotor neuron axon guidance///cell differentiation///cell migration///cell migration involved in coronary vasculogenesis///cell migration involved in sprouting angiogenesis///cell migration involved in sprouting angiogenesis///cellular response to hepatocyte growth factor stimulus///cellular response to vascular endothelial growth factor stimulus///commissural neuron axon guidance///coronary artery morphogenesis///dendrite development///dichotomous subdivision of terminal units involved in salivary gland branching///dichotomous subdivision of terminal units involved in salivary gland branching///dorsal root ganglion morphogenesis///endothelial cell chemotaxis///facial nerve structural organization///facioacoustic ganglion development///gonadotrophin-releasing hormone neuronal migration to the hypothalamus///heart development///hepatocyte growth factor receptor signaling pathway///motor neuron axon guidance///multicellular organism development///negative regulation of axon extension///negative regulation of axon extension involved in axon guidance///negative regulation of axon extension involved in axon guidance///negative regulation of extrinsic apoptotic signaling pathway///negative regulation of neuron apoptotic process///nerve development///nervous system development///neural crest cell migration involved in autonomic nervous system development///neuron development///neuron migration///organ morphogenesis///otic placode development///patterning of blood vessels///platelet-derived growth factor receptor signaling pathway///positive chemotaxis///positive regulation of ERK1 and ERK2 cascade///positive regulation of axon extension involved in axon guidance///positive regulation of peptidyl-tyrosine phosphorylation///positive regulation of peptidyl-tyrosine phosphorylation///positive regulation of retinal ganglion cell axon guidance///protein localization to early endosome///regulation of axon extension involved in axon guidance///regulation of retinal ganglion cell axon guidance///renal artery morphogenesis///retina vasculature morphogenesis in camera-type eye///retinal ganglion cell axon guidance///semaphorin-plexin signaling pathway///semaphorin-plexin signaling pathway///semaphorin-plexin signaling pathway involved in axon guidance///semaphorin-plexin signaling pathway involved in neuron projection guidance///sensory neuron axon guidance///sprouting angiogenesis///sympathetic ganglion development///sympathetic nervous system development///sympathetic neuron projection extension///sympathetic neuron projection guidance///toxin transport///trigeminal ganglion development///trigeminal nerve morphogenesis///trigeminal nerve structural organization///vascular endothelial growth factor receptor signaling pathway///vascular endothelial growth factor receptor signaling pathway///vascular endothelial growth factor signaling pathway///ventral trunk neural crest cell migration///vestibulocochlear nerve structural organization | axon///axon///cell surface///cytosol///early endosome///extracellular space///focal adhesion///growth cone///integral component of membrane///membrane///neurofilament///neuronal cell body///plasma membrane///plasma membrane///sorting endosome |
| 1416572_at | 0.000306 | 2.86E-07 | 1.72E+01 | 7.497247 | 4.19 | Mmp14 | matrix metallopeptidase 14 (membrane-inserted) | calcium ion binding///hydrolase activity///integrin binding///metal ion binding///metalloendopeptidase activity///metallopeptidase activity///peptidase activator activity///peptidase activity///zinc ion binding | bone development///branching morphogenesis of an epithelial tube///cell migration///chondrocyte proliferation///collagen catabolic process///craniofacial suture morphogenesis///embryonic cranial skeleton morphogenesis///endochondral ossification///lung development///negative regulation of Notch signaling pathway///negative regulation of focal adhesion assembly///ossification///positive regulation of B cell differentiation///positive regulation of cell growth///positive regulation of cell migration///positive regulation of myotube differentiation///proteolysis///response to hormone///tissue remodeling///zymogen activation | cytoplasm///colocalizes_with cytoplasm///cytoplasmic vesicle///extracellular matrix///focal adhesion///integral component of membrane///macropinosome///membrane///plasma membrane |
| 1438031_at | 0.000306 | 2.92E-07 | -1.72E+01 | 7.480803 | -6.58 | Rasgrp3 | RAS, guanyl releasing protein 3 | GTPase activator activity///Ras GTPase binding///Ras GTPase binding///Ras guanyl-nucleotide exchange factor activity///kinase binding | Ras protein signal transduction///regulation of GTPase activity | cytoplasm///guanyl-nucleotide exchange factor complex///perinuclear region of cytoplasm |
| 1431095_a_at | 0.000306 | 2.92E-07 | 1.72E+01 | 7.479474 | 4.01 | Herc6 | hect domain and RLD 6 | UDP-N-acetylmuramoylalanyl-D-glutamyl-2,6-diaminopimelate-D-alanyl-D-alanine ligase activity///coenzyme F420-0 gamma-glutamyl ligase activity///coenzyme F420-2 alpha-glutamyl ligase activity///cyclin binding///ligase activity///protein-glutamic acid ligase activity///protein-glycine ligase activity///protein-glycine ligase activity, elongating///protein-glycine ligase activity, initiating///ribosomal S6-glutamic acid ligase activity///tubulin-glutamic acid ligase activity///tubulin-glycine ligase activity///ubiquitin protein ligase activity///ubiquitin-protein transferase activity///ubiquitin-protein transferase activity | hematopoietic progenitor cell differentiation///immune system process///innate immune response///protein ubiquitination///protein ubiquitination involved in ubiquitin-dependent protein catabolic process | cytoplasm///cytoplasm///nucleus///nucleus |
| 1421066_at | 0.000307 | 2.99E-07 | 1.71E+01 | 7.458991 | 2.47 | Jak2 | Janus kinase 2 | ATP binding///SH2 domain binding///acetylcholine receptor binding///growth hormone receptor binding///growth hormone receptor binding///heme binding///histone binding///histone kinase activity (H3-Y41 specific)///histone kinase activity (H3-Y41 specific)///insulin receptor substrate binding///interleukin-12 receptor binding///kinase activity///metal ion binding///non-membrane spanning protein tyrosine kinase activity///nucleotide binding///peptide hormone receptor binding///phosphatidylinositol 3-kinase binding///protein C-terminus binding///protein binding///protein kinase activity///protein kinase binding///protein tyrosine kinase activity///protein tyrosine kinase activity///protein tyrosine kinase activity///receptor binding///transferase activity///type 1 angiotensin receptor binding | G-protein coupled receptor signaling pathway///JAK-STAT cascade///JAK-STAT cascade///JAK-STAT cascade///JAK-STAT cascade involved in growth hormone signaling pathway///JAK-STAT cascade involved in growth hormone signaling pathway///JAK-STAT cascade involved in growth hormone signaling pathway///STAT protein import into nucleus///activation of JAK2 kinase activity///activation of MAPKK activity///activation of cysteine-type endopeptidase activity involved in apoptotic process///activation of cysteine-type endopeptidase activity involved in apoptotic signaling pathway///adaptive immune response///axon regeneration///cell differentiation///cell migration///cellular response to dexamethasone stimulus///cellular response to interleukin-3///cellular response to lipopolysaccharide///covalent chromatin modification///cytokine-mediated signaling pathway///cytokine-mediated signaling pathway///cytokine-mediated signaling pathway///enzyme linked receptor protein signaling pathway///erythrocyte differentiation///extrinsic apoptotic signaling pathway///growth hormone receptor signaling pathway///histone H3-Y41 phosphorylation///hormone-mediated signaling pathway///host programmed cell death induced by symbiont///immune system process///inflammatory response///innate immune response///interferon-gamma-mediated signaling pathway///interleukin-12-mediated signaling pathway///intracellular signal transduction///intrinsic apoptotic signaling pathway in response to oxidative stress///mammary gland epithelium development///mineralocorticoid receptor signaling pathway///myeloid cell differentiation///negative regulation of DNA binding///negative regulation of apoptotic process///negative regulation of cardiac muscle cell apoptotic process///negative regulation of cell death///negative regulation of cell proliferation///negative regulation of cell-cell adhesion///negative regulation of heart contraction///negative regulation of neuron apoptotic process///peptidyl-tyrosine autophosphorylation///peptidyl-tyrosine phosphorylation///phosphorylation///platelet-derived growth factor receptor signaling pathway///positive regulation of DNA binding///positive regulation of apoptotic process///positive regulation of apoptotic signaling pathway///positive regulation of cell activation///positive regulation of cell differentiation///positive regulation of cell migration///positive regulation of cell proliferation///positive regulation of cell-substrate adhesion///positive regulation of cytosolic calcium ion concentration///positive regulation of epithelial cell apoptotic process///positive regulation of growth factor dependent skeletal muscle satellite cell proliferation///positive regulation of growth hormone receptor signaling pathway///positive regulation of inflammatory response///positive regulation of insulin secretion///positive regulation of interleukin-1 beta production///positive regulation of nitric oxide biosynthetic process///positive regulation of nitric-oxide synthase biosynthetic process///positive regulation of nitric-oxide synthase biosynthetic process///positive regulation of peptidyl-tyrosine phosphorylation///positive regulation of phosphatidylinositol 3-kinase signaling///positive regulation of phosphoprotein phosphatase activity///positive regulation of protein import into nucleus, translocation///positive regulation of sequence-specific DNA binding transcription factor activity///positive regulation of transcription from RNA polymerase II promoter///positive regulation of tumor necrosis factor production///positive regulation of tyrosine phosphorylation of Stat3 protein///positive regulation of tyrosine phosphorylation of Stat5 protein///positive regulation of vascular smooth muscle cell proliferation///post-embryonic hemopoiesis///protein autophosphorylation///protein autophosphorylation///protein phosphorylation///regulation of cell adhesion///regulation of inflammatory response///response to antibiotic///response to granulocyte macrophage colony-stimulating factor///response to hydroperoxide///response to interleukin-12///response to lipopolysaccharide///response to oxidative stress///response to tumor necrosis factor///signal transduction///tumor necrosis factor-mediated signaling pathway///tyrosine phosphorylation of STAT protein///tyrosine phosphorylation of STAT protein///tyrosine phosphorylation of Stat1 protein///tyrosine phosphorylation of Stat3 protein///tyrosine phosphorylation of Stat5 protein | caveola///cytoplasm///cytoplasm///cytoskeleton///cytosol///extrinsic component of cytoplasmic side of plasma membrane///membrane///membrane raft///nuclear matrix///nucleus///nucleus |
| 1419202_at | 0.00031 | 3.09E-07 | 1.71E+01 | 7.431324 | 5.45 | Cst7 | cystatin F (leukocystatin) | cysteine-type endopeptidase inhibitor activity///peptidase inhibitor activity | negative regulation of peptidase activity | extracellular region |
| 1450788_at | 0.000324 | 3.34E-07 | 1.69E+01 | 7.363857 | 7.19 | Saa1 | serum amyloid A 1 | G-protein coupled receptor binding///chemoattractant activity///heparin binding///protein binding | acute-phase response///cell chemotaxis///cholesterol metabolic process | cytoplasmic microtubule///extracellular exosome///extracellular region///extracellular space///high-density lipoprotein particle |
| 1417399_at | 0.000324 | 3.37E-07 | -1.69E+01 | 7.356022 | -3.27 | Gas6 | growth arrest specific 6 | binding, bridging///calcium ion binding///cysteine-type endopeptidase inhibitor activity involved in apoptotic process///metal ion binding///phosphatidylserine binding///protein tyrosine kinase activator activity///receptor agonist activity///receptor agonist activity///receptor binding///receptor tyrosine kinase binding///voltage-gated calcium channel activity | B cell chemotaxis///activation of protein kinase B activity///apoptotic cell clearance///apoptotic cell clearance///blood coagulation///calcium ion transmembrane transport///cell-substrate adhesion///cellular response to drug///cellular response to glucose stimulus///cellular response to growth factor stimulus///cellular response to growth factor stimulus///cellular response to interferon-alpha///cellular response to starvation///cellular response to vitamin K///enzyme linked receptor protein signaling pathway///fusion of virus membrane with host plasma membrane///hematopoietic stem cell migration to bone marrow///macrophage cytokine production///negative regulation of apoptotic process///negative regulation of apoptotic process///negative regulation of apoptotic process///negative regulation of apoptotic process///negative regulation of biomineral tissue development///negative regulation of cysteine-type endopeptidase activity involved in apoptotic process///negative regulation of dendritic cell apoptotic process///negative regulation of endothelial cell apoptotic process///negative regulation of fibroblast apoptotic process///negative regulation of interferon-gamma production///negative regulation of interleukin-1 secretion///negative regulation of interleukin-6 production///negative regulation of interleukin-6 secretion///negative regulation of oligodendrocyte apoptotic process///negative regulation of protein import into nucleus, translocation///negative regulation of sequence-specific DNA binding transcription factor activity///negative regulation of transcription, DNA-templated///negative regulation of tumor necrosis factor production///negative regulation of tumor necrosis factor-mediated signaling pathway///neuron migration///peptidyl-serine phosphorylation///phagocytosis///positive regulation of ERK1 and ERK2 cascade///positive regulation of TOR signaling///positive regulation of cytokine-mediated signaling pathway///positive regulation of dendritic cell chemotaxis///positive regulation of fibroblast proliferation///positive regulation of gene expression///positive regulation of glomerular filtration///positive regulation of natural killer cell differentiation///positive regulation of peptidyl-serine phosphorylation///positive regulation of phagocytosis///positive regulation of protein export from nucleus///positive regulation of protein kinase B signaling///positive regulation of protein kinase activity///positive regulation of protein phosphorylation///positive regulation of protein tyrosine kinase activity///protein kinase B signaling///protein kinase B signaling///protein kinase B signaling///protein phosphorylation///protein targeting to plasma membrane///receptor-mediated virion attachment to host cell///regulation of growth///signal transduction///viral entry into host cell///viral genome replication | cytoplasm///extracellular exosome///extracellular region///extracellular space///extracellular space///intracellular |
| 1421008_at | 0.000343 | 3.65E-07 | 1.67E+01 | 7.288443 | 5.71 | Rsad2 | radical S-adenosyl methionine domain containing 2 | 4 iron, 4 sulfur cluster binding///catalytic activity///iron-sulfur cluster binding///metal ion binding///protein binding///protein self-association | CD4-positive, alpha-beta T cell activation///CD4-positive, alpha-beta T cell differentiation///defense response to virus///defense response to virus///immune system process///innate immune response///negative regulation of protein secretion///negative regulation of viral genome replication///negative regulation of viral genome replication///ossification///positive regulation of T-helper 2 cell cytokine production///positive regulation of toll-like receptor 7 signaling pathway///positive regulation of toll-like receptor 9 signaling pathway///regulation of ossification///response to virus///response to virus | endoplasmic reticulum///endoplasmic reticulum///endoplasmic reticulum membrane///lipid particle///membrane///mitochondrial inner membrane///mitochondrial outer membrane///mitochondrion |
| 1436058_at | 0.000346 | 3.76E-07 | 1.66E+01 | 7.262539 | 4.46 | Rsad2 | radical S-adenosyl methionine domain containing 2 | 4 iron, 4 sulfur cluster binding///catalytic activity///iron-sulfur cluster binding///metal ion binding///protein binding///protein self-association | CD4-positive, alpha-beta T cell activation///CD4-positive, alpha-beta T cell differentiation///defense response to virus///defense response to virus///immune system process///innate immune response///negative regulation of protein secretion///negative regulation of viral genome replication///negative regulation of viral genome replication///ossification///positive regulation of T-helper 2 cell cytokine production///positive regulation of toll-like receptor 7 signaling pathway///positive regulation of toll-like receptor 9 signaling pathway///regulation of ossification///response to virus///response to virus | endoplasmic reticulum///endoplasmic reticulum///endoplasmic reticulum membrane///lipid particle///membrane///mitochondrial inner membrane///mitochondrial outer membrane///mitochondrion |
| 1452299_at | 0.000358 | 3.97E-07 | -1.65E+01 | 7.214368 | -3.43 | Wwp1 | WW domain containing E3 ubiquitin protein ligase 1 | UDP-N-acetylmuramoylalanyl-D-glutamyl-2,6-diaminopimelate-D-alanyl-D-alanine ligase activity///coenzyme F420-0 gamma-glutamyl ligase activity///coenzyme F420-2 alpha-glutamyl ligase activity///ligase activity///protein binding///protein-glutamic acid ligase activity///protein-glycine ligase activity///protein-glycine ligase activity, elongating///protein-glycine ligase activity, initiating///ribosomal S6-glutamic acid ligase activity///tubulin-glutamic acid ligase activity///tubulin-glycine ligase activity///ubiquitin protein ligase activity///ubiquitin-protein transferase activity///ubiquitin-protein transferase activity | T cell differentiation///lung development///negative regulation of transcription, DNA-templated///proteasome-mediated ubiquitin-dependent protein catabolic process///protein ubiquitination///protein ubiquitination///protein ubiquitination involved in ubiquitin-dependent protein catabolic process | cytoplasm///extracellular exosome///membrane///nucleus///plasma membrane |
| 1452248_at | 0.000361 | 4.08E-07 | -1.64E+01 | 7.190161 | -2.58 | Plekhg5 | pleckstrin homology domain containing, family G (with RhoGef domain) member 5 | Rho guanyl-nucleotide exchange factor activity///protein binding///signal transducer activity | endothelial cell chemotaxis///endothelial cell migration///positive regulation of I-kappaB kinase/NF-kappaB signaling///regulation of Rho protein signal transduction | cell junction///cell projection///cell-cell junction///cytoplasm///endocytic vesicle///lamellipodium///membrane///plasma membrane |
| 1453757_at | 0.000375 | 4.32E-07 | 1.63E+01 | 7.140758 | 4 | Herc6 | hect domain and RLD 6 | UDP-N-acetylmuramoylalanyl-D-glutamyl-2,6-diaminopimelate-D-alanyl-D-alanine ligase activity///coenzyme F420-0 gamma-glutamyl ligase activity///coenzyme F420-2 alpha-glutamyl ligase activity///cyclin binding///ligase activity///protein-glutamic acid ligase activity///protein-glycine ligase activity///protein-glycine ligase activity, elongating///protein-glycine ligase activity, initiating///ribosomal S6-glutamic acid ligase activity///tubulin-glutamic acid ligase activity///tubulin-glycine ligase activity///ubiquitin protein ligase activity///ubiquitin-protein transferase activity///ubiquitin-protein transferase activity | hematopoietic progenitor cell differentiation///immune system process///innate immune response///protein ubiquitination///protein ubiquitination involved in ubiquitin-dependent protein catabolic process | cytoplasm///cytoplasm///nucleus///nucleus |
| 1427098_at | 0.000408 | 4.81E-07 | -1.61E+01 | 7.046415 | -2.48 | Wwp1 | WW domain containing E3 ubiquitin protein ligase 1 | UDP-N-acetylmuramoylalanyl-D-glutamyl-2,6-diaminopimelate-D-alanyl-D-alanine ligase activity///coenzyme F420-0 gamma-glutamyl ligase activity///coenzyme F420-2 alpha-glutamyl ligase activity///ligase activity///protein binding///protein-glutamic acid ligase activity///protein-glycine ligase activity///protein-glycine ligase activity, elongating///protein-glycine ligase activity, initiating///ribosomal S6-glutamic acid ligase activity///tubulin-glutamic acid ligase activity///tubulin-glycine ligase activity///ubiquitin protein ligase activity///ubiquitin-protein transferase activity///ubiquitin-protein transferase activity | T cell differentiation///lung development///negative regulation of transcription, DNA-templated///proteasome-mediated ubiquitin-dependent protein catabolic process///protein ubiquitination///protein ubiquitination///protein ubiquitination involved in ubiquitin-dependent protein catabolic process | cytoplasm///extracellular exosome///membrane///nucleus///plasma membrane |
| 1421992_a_at | 0.000408 | 4.91E-07 | -1.60E+01 | 7.028759 | -3.8 | Igfbp4 | insulin-like growth factor binding protein 4 | growth factor binding///insulin-like growth factor I binding///insulin-like growth factor II binding///insulin-like growth factor binding | positive regulation of MAPK cascade///positive regulation of insulin-like growth factor receptor signaling pathway///regulation of cell growth///regulation of glucose metabolic process///regulation of growth///type B pancreatic cell proliferation | extracellular region///extracellular space |
| 1449449_at | 0.000408 | 4.98E-07 | 1.60E+01 | 7.016439 | 8.02 | Ptges | prostaglandin E synthase | glutathione binding///isomerase activity///prostaglandin-D synthase activity///prostaglandin-E synthase activity | fatty acid biosynthetic process///fatty acid metabolic process///lipid metabolic process///negative regulation of cell proliferation///prostaglandin biosynthetic process///prostaglandin metabolic process | cytoplasm///integral component of membrane///intracellular membrane-bounded organelle///membrane///nuclear envelope lumen///NOT nucleus///perinuclear region of cytoplasm |
| 1440451_at | 0.000418 | 5.19E-07 | 1.59E+01 | 6.978909 | 2.76 | Ankrd66 | ankyrin repeat domain 66 | | |  |
| 1449134_s_at | 0.00045 | 5.93E-07 | 1.56E+01 | 6.861284 | 4.4 | Spic | Spi-C transcription factor (Spi-1/PU.1 related) | DNA binding///RNA polymerase II transcription factor activity, sequence-specific DNA binding///sequence-specific DNA binding///transcription factor activity, sequence-specific DNA binding | blastocyst development///cell differentiation///positive regulation of transcription from RNA polymerase II promoter///regulation of transcription, DNA-templated///transcription, DNA-templated | nucleus |
| 1421065_at | 0.00045 | 6.00E-07 | 1.56E+01 | 6.850975 | 2.69 | Jak2 | Janus kinase 2 | ATP binding///SH2 domain binding///acetylcholine receptor binding///growth hormone receptor binding///growth hormone receptor binding///heme binding///histone binding///histone kinase activity (H3-Y41 specific)///histone kinase activity (H3-Y41 specific)///insulin receptor substrate binding///interleukin-12 receptor binding///kinase activity///metal ion binding///non-membrane spanning protein tyrosine kinase activity///nucleotide binding///peptide hormone receptor binding///phosphatidylinositol 3-kinase binding///protein C-terminus binding///protein binding///protein kinase activity///protein kinase binding///protein tyrosine kinase activity///protein tyrosine kinase activity///protein tyrosine kinase activity///receptor binding///transferase activity///type 1 angiotensin receptor binding | G-protein coupled receptor signaling pathway///JAK-STAT cascade///JAK-STAT cascade///JAK-STAT cascade///JAK-STAT cascade involved in growth hormone signaling pathway///JAK-STAT cascade involved in growth hormone signaling pathway///JAK-STAT cascade involved in growth hormone signaling pathway///STAT protein import into nucleus///activation of JAK2 kinase activity///activation of MAPKK activity///activation of cysteine-type endopeptidase activity involved in apoptotic process///activation of cysteine-type endopeptidase activity involved in apoptotic signaling pathway///adaptive immune response///axon regeneration///cell differentiation///cell migration///cellular response to dexamethasone stimulus///cellular response to interleukin-3///cellular response to lipopolysaccharide///covalent chromatin modification///cytokine-mediated signaling pathway///cytokine-mediated signaling pathway///cytokine-mediated signaling pathway///enzyme linked receptor protein signaling pathway///erythrocyte differentiation///extrinsic apoptotic signaling pathway///growth hormone receptor signaling pathway///histone H3-Y41 phosphorylation///hormone-mediated signaling pathway///host programmed cell death induced by symbiont///immune system process///inflammatory response///innate immune response///interferon-gamma-mediated signaling pathway///interleukin-12-mediated signaling pathway///intracellular signal transduction///intrinsic apoptotic signaling pathway in response to oxidative stress///mammary gland epithelium development///mineralocorticoid receptor signaling pathway///myeloid cell differentiation///negative regulation of DNA binding///negative regulation of apoptotic process///negative regulation of cardiac muscle cell apoptotic process///negative regulation of cell death///negative regulation of cell proliferation///negative regulation of cell-cell adhesion///negative regulation of heart contraction///negative regulation of neuron apoptotic process///peptidyl-tyrosine autophosphorylation///peptidyl-tyrosine phosphorylation///phosphorylation///platelet-derived growth factor receptor signaling pathway///positive regulation of DNA binding///positive regulation of apoptotic process///positive regulation of apoptotic signaling pathway///positive regulation of cell activation///positive regulation of cell differentiation///positive regulation of cell migration///positive regulation of cell proliferation///positive regulation of cell-substrate adhesion///positive regulation of cytosolic calcium ion concentration///positive regulation of epithelial cell apoptotic process///positive regulation of growth factor dependent skeletal muscle satellite cell proliferation///positive regulation of growth hormone receptor signaling pathway///positive regulation of inflammatory response///positive regulation of insulin secretion///positive regulation of interleukin-1 beta production///positive regulation of nitric oxide biosynthetic process///positive regulation of nitric-oxide synthase biosynthetic process///positive regulation of nitric-oxide synthase biosynthetic process///positive regulation of peptidyl-tyrosine phosphorylation///positive regulation of phosphatidylinositol 3-kinase signaling///positive regulation of phosphoprotein phosphatase activity///positive regulation of protein import into nucleus, translocation///positive regulation of sequence-specific DNA binding transcription factor activity///positive regulation of transcription from RNA polymerase II promoter///positive regulation of tumor necrosis factor production///positive regulation of tyrosine phosphorylation of Stat3 protein///positive regulation of tyrosine phosphorylation of Stat5 protein///positive regulation of vascular smooth muscle cell proliferation///post-embryonic hemopoiesis///protein autophosphorylation///protein autophosphorylation///protein phosphorylation///regulation of cell adhesion///regulation of inflammatory response///response to antibiotic///response to granulocyte macrophage colony-stimulating factor///response to hydroperoxide///response to interleukin-12///response to lipopolysaccharide///response to oxidative stress///response to tumor necrosis factor///signal transduction///tumor necrosis factor-mediated signaling pathway///tyrosine phosphorylation of STAT protein///tyrosine phosphorylation of STAT protein///tyrosine phosphorylation of Stat1 protein///tyrosine phosphorylation of Stat3 protein///tyrosine phosphorylation of Stat5 protein | caveola///cytoplasm///cytoplasm///cytoskeleton///cytosol///extrinsic component of cytoplasmic side of plasma membrane///membrane///membrane raft///nuclear matrix///nucleus///nucleus |
| 1433453_a_at | 0.00045 | 6.00E-07 | 1.56E+01 | 6.850396 | 4.61 | Abtb2 | ankyrin repeat and BTB (POZ) domain containing 2 | protein heterodimerization activity///ubiquitin protein ligase binding | cellular response to toxic substance///proteasome-mediated ubiquitin-dependent protein catabolic process///protein ubiquitination involved in ubiquitin-dependent protein catabolic process///regulation of proteolysis | SCF ubiquitin ligase complex///cytoplasm///nucleoplasm///nucleus///nucleus |
| 1417929_at | 0.00045 | 6.05E-07 | 1.56E+01 | 6.842949 | 2.25 | Slc7a8 | solute carrier family 7 (cationic amino acid transporter, y+ system), member 8 | L-amino acid transmembrane transporter activity///amine transmembrane transporter activity///amino acid transmembrane transporter activity///antiporter activity///neutral amino acid transmembrane transporter activity///organic cation transmembrane transporter activity///toxin transporter activity | L-amino acid transport///amino acid transmembrane transport///amino acid transport///amino acid transport///neutral amino acid transport///transport | cytoplasm///extracellular exosome///integral component of membrane///integral component of plasma membrane///membrane///plasma membrane///plasma membrane |
| 1420499_at | 0.00045 | 6.08E-07 | 1.55E+01 | 6.83853 | 2.75 | Gch1 | GTP cyclohydrolase 1 | GTP binding///GTP binding///GTP cyclohydrolase I activity///GTP cyclohydrolase I activity///GTP-dependent protein binding///calcium ion binding///catalytic activity///coenzyme binding///hydrolase activity///metal ion binding///nucleotide binding///protein homodimerization activity///translation initiation factor binding///zinc ion binding///zinc ion binding | dihydrobiopterin metabolic process///dopamine biosynthetic process///metabolic process///negative regulation of blood pressure///neuromuscular process controlling posture///positive regulation of nitric-oxide synthase activity///protein complex assembly///protein heterooligomerization///protein homooligomerization///pteridine-containing compound biosynthetic process///regulation of blood pressure///regulation of blood pressure///regulation of lung blood pressure///regulation of removal of superoxide radicals///response to interferon-gamma///response to pain///response to tumor necrosis factor///tetrahydrobiopterin biosynthetic process///tetrahydrobiopterin biosynthetic process///tetrahydrobiopterin biosynthetic process///tetrahydrofolate biosynthetic process///vasodilation | cytoplasm///cytoplasm///cytoplasmic vesicle///cytosol///nuclear membrane///nucleoplasm///nucleus///protein complex |
| 1434763_at | 0.000462 | 6.46E-07 | 1.54E+01 | 6.784258 | 2.39 | Orai2 | ORAI calcium release-activated calcium modulator 2 | store-operated calcium channel activity | store-operated calcium entry | growth cone///integral component of membrane///membrane |
| 1449875_s_at | 0.000462 | 6.50E-07 | 1.54E+01 | 6.778606 | 2.96 | H2-T9///H2-T22///H2-T10 | histocompatibility 2, T region locus 9///histocompatibility 2, T region locus 22///histocompatibility 2, T region locus 10 | peptide antigen binding///receptor binding | antigen processing and presentation of peptide antigen via MHC class I | plasma membrane |
| 1421991_a_at | 0.000462 | 6.61E-07 | -1.54E+01 | 6.76357 | -4.41 | Igfbp4 | insulin-like growth factor binding protein 4 | growth factor binding///insulin-like growth factor I binding///insulin-like growth factor II binding///insulin-like growth factor binding | positive regulation of MAPK cascade///positive regulation of insulin-like growth factor receptor signaling pathway///regulation of cell growth///regulation of glucose metabolic process///regulation of growth///type B pancreatic cell proliferation | extracellular region///extracellular space |
| 1449453_at | 0.000462 | 6.82E-07 | 1.53E+01 | 6.736317 | 5.34 | Bst1 | bone marrow stromal cell antigen 1 | NAD(P)+ nucleosidase activity///NAD+ nucleosidase activity///hydrolase activity///phosphorus-oxygen lyase activity///transferase activity | positive regulation of B cell proliferation | anchored component of membrane///extracellular exosome///membrane///plasma membrane |
| 1429646_at | 0.000462 | 6.94E-07 | 1.53E+01 | 6.720467 | 2.69 | Prss46 | protease, serine 46 | hydrolase activity///peptidase activity///serine-type endopeptidase activity///serine-type peptidase activity | proteolysis | integral component of membrane///membrane |
| 1434191_at | 0.000462 | 6.99E-07 | -1.52E+01 | 6.713701 | -3.55 | Agmo | alkylglycerol monooxygenase | glyceryl-ether monooxygenase activity///glyceryl-ether monooxygenase activity///iron ion binding///oxidoreductase activity | ether lipid metabolic process///fatty acid biosynthetic process///membrane lipid metabolic process///membrane lipid metabolic process///oxidation-reduction process | endoplasmic reticulum///endoplasmic reticulum///integral component of membrane///membrane |
| 1450033_a_at | 0.000462 | 7.02E-07 | 1.52E+01 | 6.710183 | 3.9 | Stat1 | signal transducer and activator of transcription 1 | CCR5 chemokine receptor binding///DNA binding///RNA polymerase II core promoter proximal region sequence-specific DNA binding///RNA polymerase II core promoter sequence-specific DNA binding///cadherin binding involved in cell-cell adhesion///double-stranded DNA binding///enzyme binding///identical protein binding///nuclear hormone receptor binding///protein binding///protein homodimerization activity///protein phosphatase 2A binding///sequence-specific DNA binding///signal transducer activity///transcription factor activity, RNA polymerase II core promoter sequence-specific///transcription factor activity, sequence-specific DNA binding///transcription factor activity, sequence-specific DNA binding///tumor necrosis factor receptor binding | JAK-STAT cascade///activation of cysteine-type endopeptidase activity involved in apoptotic process///blood circulation///cell-cell adhesion///cellular response to cytokine stimulus///cellular response to insulin stimulus///cellular response to interferon-beta///cellular response to lipopolysaccharide///cellular response to organic cyclic compound///cytokine-mediated signaling pathway///interferon-gamma-mediated signaling pathway///interferon-gamma-mediated signaling pathway///lipopolysaccharide-mediated signaling pathway///macrophage derived foam cell differentiation///negative regulation by virus of viral protein levels in host cell///negative regulation of I-kappaB kinase/NF-kappaB signaling///negative regulation of angiogenesis///negative regulation of endothelial cell proliferation///negative regulation of macrophage fusion///positive regulation of cell proliferation///positive regulation of smooth muscle cell proliferation///positive regulation of transcription from RNA polymerase II promoter///positive regulation of transcription, DNA-templated///regulation of transcription, DNA-templated///renal tubule development///response to bacterium///response to cAMP///response to cytokine///response to drug///response to exogenous dsRNA///response to interferon-beta///response to lipopolysaccharide///response to mechanical stimulus///response to nutrient///response to peptide hormone///response to type I interferon///signal transduction///transcription, DNA-templated///tumor necrosis factor-mediated signaling pathway///type I interferon signaling pathway | axon///cell-cell adherens junction///cytoplasm///cytoplasm///dendrite///nuclear chromatin///nucleolus///nucleoplasm///nucleus///nucleus///perinuclear region of cytoplasm |
| 1436448_a_at | 0.000462 | 7.06E-07 | -1.52E+01 | 6.704238 | -4.71 | Ptgs1 | prostaglandin-endoperoxide synthase 1 | dioxygenase activity///heme binding///lipid binding///metal ion binding///oxidoreductase activity///peroxidase activity///prostaglandin-endoperoxide synthase activity | cyclooxygenase pathway///fatty acid biosynthetic process///fatty acid metabolic process///inflammatory response///keratinocyte differentiation///learning///lipid metabolic process///maintenance of blood-brain barrier///memory///negative regulation of epinephrine secretion///negative regulation of norepinephrine secretion///oxidation-reduction process///positive regulation of smooth muscle contraction///positive regulation of vasoconstriction///prostaglandin biosynthetic process///prostaglandin biosynthetic process///prostaglandin metabolic process///regulation of blood pressure///regulation of cell proliferation///response to oxidative stress///sensory perception of pain | Golgi apparatus///cytoplasm///cytoplasm///endoplasmic reticulum///extracellular exosome///intracellular membrane-bounded organelle///membrane///nuclear envelope///NOT nucleus///photoreceptor outer segment |
| 1438027_at | 0.000482 | 7.54E-07 | 1.51E+01 | 6.645072 | 2.36 |  |  |  |  |  |
| 1440879_at | 0.000482 | 7.59E-07 | -1.51E+01 | 6.639791 | -3.89 | Abca9 | ATP-binding cassette, sub-family A (ABC1), member 9 | ATP binding///ATPase activity///ATPase activity, coupled to transmembrane movement of substances///nucleotide binding///transporter activity | lipid transport///transport | integral component of membrane///membrane///mitochondrial inner membrane///mitochondrion |
| 1450627_at | 0.000509 | 8.13E-07 | -1.49E+01 | 6.57669 | -3.65 | Ank | progressive ankylosis | inorganic diphosphate transmembrane transporter activity///inorganic diphosphate transmembrane transporter activity///inorganic phosphate transmembrane transporter activity///phosphate ion transmembrane transporter activity | phosphate ion transmembrane transport///phosphate ion transport///regulation of bone mineralization///transport | integral component of membrane///integral component of membrane///integral component of plasma membrane///membrane///plasma membrane |
| 1445882_at | 0.000527 | 8.53E-07 | -1.48E+01 | 6.53393 | -2.77 | Cd300lb | CD300 molecule like family member B | protein binding | cellular response to lipopolysaccharide///immune system process///neutrophil mediated immunity///positive regulation of mast cell activation | integral component of membrane///membrane///plasma membrane |
| 1416432_at | 0.000557 | 9.13E-07 | 1.47E+01 | 6.471113 | 3.18 | Pfkfb3 | 6-phosphofructo-2-kinase/fructose-2,6-biphosphatase 3 | 6-phosphofructo-2-kinase activity///fructose-2,6-bisphosphate 2-phosphatase activity | fructose 2,6-bisphosphate metabolic process | nucleoplasm |
| 1424826_s_at | 0.000558 | 9.28E-07 | -1.47E+01 | 6.457001 | -2.62 | Mtss1 | metastasis suppressor 1 | actin binding///actin filament binding///actin monomer binding///identical protein binding///receptor binding | actin cytoskeleton organization///actin filament organization///actin filament polymerization///adherens junction maintenance///bone mineralization///cellular response to fluid shear stress///epithelial cell proliferation involved in renal tubule morphogenesis///glomerulus morphogenesis///in utero embryonic development///magnesium ion homeostasis///muscle organ development///negative regulation of epithelial cell proliferation///nephron tubule epithelial cell differentiation///nervous system development///plasma membrane organization///positive regulation of actin filament bundle assembly///positive regulation of defense response to virus by host///renal system development///renal tubule morphogenesis///xenophagy | actin cytoskeleton///adherens junction///cytoplasm///cytoskeleton |
| 1455285_at | 0.000568 | 9.72E-07 | 1.46E+01 | 6.41402 | 2.56 | Slc31a1 | solute carrier family 31, member 1 | copper ion transmembrane transporter activity///copper uptake transmembrane transporter activity///copper uptake transmembrane transporter activity///protein binding | cellular copper ion homeostasis///cellular copper ion homeostasis///cellular response to cisplatin///copper ion import///copper ion import///copper ion import into cell///copper ion transmembrane transport///copper ion transport///drug transmembrane transport///ion transport///transport | cytoplasm///integral component of membrane///late endosome///membrane///neuronal cell body///plasma membrane///recycling endosome |
| 1421217_a_at | 0.000568 | 9.87E-07 | 1.45E+01 | 6.400452 | 2.75 | Lgals9 | lectin, galactose binding, soluble 9 | carbohydrate binding///carbohydrate binding///enzyme binding///galactoside binding///galactoside binding///protein binding///protein serine/threonine kinase activator activity///receptor binding | cellular response to virus///chemotaxis///female pregnancy///heterophilic cell-cell adhesion via plasma membrane cell adhesion molecules///immune system process///maintenance of protein location///negative regulation of CD4-positive, alpha-beta T cell proliferation///negative regulation of gene expression///negative regulation of inflammatory response///negative regulation of interferon-gamma production///negative regulation of interferon-gamma secretion///negative regulation of natural killer cell activation///negative regulation of natural killer cell degranulation///positive regulation of T cell migration///positive regulation of chemokine production///positive regulation of cytokine production///positive regulation of defense response to bacterium///positive regulation of gene expression///positive regulation of innate immune response///positive regulation of interleukin-1 production///positive regulation of interleukin-10 secretion///positive regulation of interleukin-6 secretion///positive regulation of macrophage activation///positive regulation of oxidoreductase activity///positive regulation of pathway-restricted SMAD protein phosphorylation///positive regulation of regulatory T cell differentiation///positive regulation of transcription regulatory region DNA binding///positive regulation of tumor necrosis factor production///NOT regulation of T cell chemotaxis///NOT regulation of T cell differentiation in thymus///NOT regulation of T-helper 17 type immune response///regulation of natural killer cell differentiation///response to lipopolysaccharide///toll-like receptor 3 signaling pathway | cytoplasm///extracellular region///intracellular///nucleus |
| 1433543_at | 0.000568 | 9.91E-07 | -1.45E+01 | 6.396126 | -3.6 | Anln | anillin, actin binding protein | actin binding///cadherin binding involved in cell-cell adhesion | cell cycle///cell division///glomerular visceral epithelial cell migration///hematopoietic progenitor cell differentiation///mitotic cytokinesis///mitotic nuclear division | actin cytoskeleton///actomyosin contractile ring///cell-cell adherens junction///cytoplasm///cytoskeleton///intracellular membrane-bounded organelle///nucleoplasm///nucleus |
| 1415849_s_at | 0.000568 | 9.96E-07 | -1.45E+01 | 6.392299 | -3.18 | Stmn1 | stathmin 1 | protein binding///tubulin binding///tubulin binding | axonogenesis///cell differentiation///microtubule depolymerization///microtubule depolymerization///mitotic cytokinesis///mitotic cytokinesis///mitotic spindle organization///multicellular organism development///negative regulation of microtubule polymerization///nervous system development///positive regulation of cellular component movement///regulation of microtubule polymerization or depolymerization | cytoplasm///cytoskeleton///cytosol///extracellular exosome///intracellular///membrane///microtubule///neuron projection |
| 1419152_at | 0.000582 | 1.03E-06 | -1.44E+01 | 6.359829 | -3.41 | 2810417H13Rik | RIKEN cDNA 2810417H13 gene | chromatin binding///chromatin binding | DNA repair///DNA replication///cellular response to DNA damage stimulus///centrosome organization///regulation of cell cycle///response to UV///translesion synthesis///translesion synthesis | cytoplasm///nucleus///nucleus///perinuclear region of cytoplasm |
| 1434139_at | 0.000593 | 1.06E-06 | 1.44E+01 | 6.330665 | 2.46 | Parp11 | poly (ADP-ribose) polymerase family, member 11 | NAD+ ADP-ribosyltransferase activity///transferase activity///transferase activity, transferring glycosyl groups | cell differentiation///mRNA transport///nuclear envelope organization///protein transport///spermatogenesis///transport | nuclear envelope///nuclear pore///nucleus |
| 1434036_at | 0.000611 | 1.11E-06 | -1.43E+01 | 6.291497 | -2.6 | Mtss1 | metastasis suppressor 1 | actin binding///actin filament binding///actin monomer binding///identical protein binding///receptor binding | actin cytoskeleton organization///actin filament organization///actin filament polymerization///adherens junction maintenance///bone mineralization///cellular response to fluid shear stress///epithelial cell proliferation involved in renal tubule morphogenesis///glomerulus morphogenesis///in utero embryonic development///magnesium ion homeostasis///muscle organ development///negative regulation of epithelial cell proliferation///nephron tubule epithelial cell differentiation///nervous system development///plasma membrane organization///positive regulation of actin filament bundle assembly///positive regulation of defense response to virus by host///renal system development///renal tubule morphogenesis///xenophagy | actin cytoskeleton///adherens junction///cytoplasm///cytoskeleton |
| 1428975_at | 0.000611 | 1.12E-06 | -1.43E+01 | 6.28026 | -2.71 | Susd3 | sushi domain containing 3 | molecular_function | biological_process | integral component of membrane///membrane///plasma membrane |
| 1451905_a_at | 0.000613 | 1.14E-06 | 1.42E+01 | 6.266503 | 6.05 | Mx1 | MX dynamin-like GTPase 1 | GTP binding///GTPase activity///nucleotide binding///protein binding | defense response to virus///immune system process///innate immune response///innate immune response///response to virus///response to virus | cytoplasm///endoplasmic reticulum///membrane///nucleus///nucleus |
| 1438948_x_at | 0.000617 | 1.24E-06 | 1.41E+01 | 6.188193 | 2.05 | Tspo | translocator protein | androgen binding///benzodiazepine receptor activity///ion channel binding | behavioral response to pain///chloride transport///contact inhibition///establishment of protein localization to mitochondrion///glial cell migration///ion transport///lipid transport///maintenance of protein location in mitochondrion///negative regulation of ATP metabolic process///negative regulation of glial cell proliferation///negative regulation of mitochondrion organization///negative regulation of mitophagy///negative regulation of nitric oxide biosynthetic process///negative regulation of protein ubiquitination///negative regulation of tumor necrosis factor production///positive regulation of apoptotic process///positive regulation of calcium ion transport///positive regulation of glial cell proliferation///positive regulation of mitochondrial depolarization///positive regulation of necrotic cell death///positive regulation of reactive oxygen species metabolic process///positive regulation of reactive oxygen species metabolic process///regulation of steroid biosynthetic process///steroid biosynthetic process///transport | cytoplasm///extracellular exosome///integral component of membrane///intracellular membrane-bounded organelle///membrane///mitochondrial outer membrane///mitochondrion///mitochondrion///nuclear membrane |
| 1435464_at | 0.000617 | 1.25E-06 | -1.41E+01 | 6.182359 | -2.19 | Smim14 | small integral membrane protein 14 | molecular_function | biological_process | endoplasmic reticulum///integral component of membrane///membrane |
| 1438931_s_at | 0.000617 | 1.26E-06 | -1.41E+01 | 6.178245 | -3.2 | Sesn1 | sestrin 1 | leucine binding///oxidoreductase activity///peroxiredoxin activity///protein binding | cellular oxidant detoxification///cellular response to amino acid stimulus///cellular response to amino acid stimulus///negative regulation of TORC1 signaling///negative regulation of TORC1 signaling///negative regulation of cell growth///oxidation-reduction process///reactive oxygen species metabolic process///regulation of protein kinase B signaling///regulation of response to reactive oxygen species | colocalizes_with GATOR2 complex///cytoplasm///nucleus |
| 1433741_at | 0.000617 | 1.26E-06 | 1.41E+01 | 6.176386 | 6.96 | Cd38 | CD38 antigen | NAD(P)+ nucleosidase activity///NAD+ nucleosidase activity///NAD+ nucleosidase activity///hydrolase activity///hydrolase activity, acting on glycosyl bonds///phosphorus-oxygen lyase activity///transferase activity | B cell receptor signaling pathway///long term synaptic depression///negative regulation of apoptotic process///negative regulation of bone resorption///negative regulation of transcription, DNA-templated///positive regulation of B cell proliferation///positive regulation of B cell proliferation///positive regulation of cell growth///positive regulation of cytosolic calcium ion concentration///positive regulation of insulin secretion///positive regulation of transcription, DNA-templated///positive regulation of vasoconstriction///response to drug///response to hormone///response to hydroperoxide///response to hypoxia | cell surface///extracellular exosome///integral component of membrane///intracellular membrane-bounded organelle///membrane///nucleus///plasma membrane///plasma membrane |
| 1419905_s_at | 0.000617 | 1.26E-06 | -1.40E+01 | 6.173894 | -7.91 | Hpgd | hydroxyprostaglandin dehydrogenase 15 (NAD) | 15-hydroxyprostaglandin dehydrogenase (NAD+) activity///15-hydroxyprostaglandin dehydrogenase (NAD+) activity///NAD binding///NAD+ binding///catalytic activity///oxidoreductase activity///prostaglandin E receptor activity | ductus arteriosus closure///fatty acid metabolic process///female pregnancy///lipid metabolic process///negative regulation of cell cycle///oxidation-reduction process///parturition///prostaglandin metabolic process///transforming growth factor beta receptor signaling pathway | basolateral plasma membrane///cytoplasm///extracellular exosome///nucleoplasm |
| 1448383_at | 0.000617 | 1.27E-06 | 1.40E+01 | 6.171545 | 4.25 | Mmp14 | matrix metallopeptidase 14 (membrane-inserted) | calcium ion binding///hydrolase activity///integrin binding///metal ion binding///metalloendopeptidase activity///metallopeptidase activity///peptidase activator activity///peptidase activity///zinc ion binding | bone development///branching morphogenesis of an epithelial tube///cell migration///chondrocyte proliferation///collagen catabolic process///craniofacial suture morphogenesis///embryonic cranial skeleton morphogenesis///endochondral ossification///lung development///negative regulation of Notch signaling pathway///negative regulation of focal adhesion assembly///ossification///positive regulation of B cell differentiation///positive regulation of cell growth///positive regulation of cell migration///positive regulation of myotube differentiation///proteolysis///response to hormone///tissue remodeling///zymogen activation | cytoplasm///colocalizes_with cytoplasm///cytoplasmic vesicle///extracellular matrix///focal adhesion///integral component of membrane///macropinosome///membrane///plasma membrane |
| 1444969_at | 0.000617 | 1.27E-06 | 1.40E+01 | 6.166364 | 3.03 | Pid1 | phosphotyrosine interaction domain containing 1 | protein binding | cellular response to cytokine stimulus///cellular response to fatty acid///cellular response to interleukin-6///cellular response to leptin stimulus///cellular response to tumor necrosis factor///mitochondrion morphogenesis///negative regulation of ATP biosynthetic process///negative regulation of establishment of protein localization to plasma membrane///negative regulation of glucose import///negative regulation of glucose import in response to insulin stimulus///negative regulation of insulin receptor signaling pathway///negative regulation of mitochondrial DNA replication///negative regulation of protein phosphorylation///positive regulation of ATP biosynthetic process///positive regulation of fat cell proliferation///positive regulation of gene expression///positive regulation of reactive oxygen species metabolic process///positive regulation of transcription from RNA polymerase II promoter///regulation of G1/S transition of mitotic cell cycle///regulation of mitochondrial membrane potential///regulation of reactive oxygen species metabolic process | cytoplasm///cytoplasm///nucleoplasm |
| 1419569_a_at | 0.000617 | 1.29E-06 | 1.40E+01 | 6.150357 | 5.82 | Isg20 | interferon-stimulated protein | 3'-5'-exoribonuclease activity///RNA binding///U1 snRNA binding///U2 snRNA binding///U3 snoRNA binding///exonuclease activity///exoribonuclease II activity///hydrolase activity///metal ion binding///nuclease activity///nucleic acid binding///single-stranded DNA 3'-5' exodeoxyribonuclease activity | DNA catabolic process, exonucleolytic///RNA catabolic process///defense response to virus///immune system process///innate immune response///negative regulation of viral genome replication///rRNA processing///response to virus | Cajal body///PML body///cytoplasm///nucleolus///nucleus |
| 1417172_at | 0.000617 | 1.31E-06 | 1.40E+01 | 6.14123 | 3.02 | Ube2l6 | ubiquitin-conjugating enzyme E2L 6 | ATP binding///ISG15 transferase activity///nucleotide binding///protein binding///transferase activity///ubiquitin binding///ubiquitin-like protein transferase activity | ISG15-protein conjugation///modification-dependent protein catabolic process | |
| 1451777_at | 0.000617 | 1.31E-06 | 1.40E+01 | 6.140218 | 4.07 | Ddx60 | DEAD (Asp-Glu-Ala-Asp) box polypeptide 60 | RNA binding///double-stranded DNA binding///double-stranded RNA binding///protein binding///single-stranded RNA binding | defense response to virus///positive regulation of MDA-5 signaling pathway///positive regulation of RIG-I signaling pathway///response to virus | cytoplasm///cytoplasm///intermediate filament cytoskeleton |
| 1438676_at | 0.000617 | 1.31E-06 | 1.40E+01 | 6.13893 | 6.55 | Gbp6 | guanylate binding protein 6 | molecular_function | adhesion of symbiont to host///cellular response to interferon-beta///cellular response to interferon-gamma///cellular response to lipopolysaccharide///defense response to Gram-positive bacterium///defense response to Gram-positive bacterium///defense response to protozoan | cytoplasmic vesicle///extracellular exosome///symbiont-containing vacuole membrane |
| 1422619_at | 0.000617 | 1.33E-06 | 1.39E+01 | 6.124641 | 4.18 | Plpp1 | phospholipid phosphatase 1 | hydrolase activity///lipid phosphatase activity///phosphatidate phosphatase activity///phosphatidate phosphatase activity///phosphatidate phosphatase activity | ceramide metabolic process///diacylglycerol biosynthetic process///lipid metabolic process///phospholipid dephosphorylation///phospholipid metabolic process///protein dephosphorylation///protein dephosphorylation///signal transduction///sphingosine metabolic process | integral component of membrane///integral component of plasma membrane///membrane///plasma membrane |
| 1424463_at | 0.000617 | 1.34E-06 | -1.39E+01 | 6.118447 | -2.23 | Mfsd6 | major facilitator superfamily domain containing 6 | | adaptive immune response///immune system process | integral component of membrane///membrane |
| 1448123_s_at | 0.000617 | 1.35E-06 | 1.39E+01 | 6.114006 | 2.23 | Tgfbi | transforming growth factor, beta induced | cell adhesion molecule binding///collagen binding///extracellular matrix binding///integrin binding | cell adhesion///cell proliferation///extracellular matrix organization | basement membrane///extracellular exosome///extracellular matrix///colocalizes_with extracellular matrix///extracellular region///extracellular space///extracellular space///proteinaceous extracellular matrix///trans-Golgi network |
| 1420380_at | 0.000617 | 1.35E-06 | 1.39E+01 | 6.108922 | 5.41 | Ccl2 | chemokine (C-C motif) ligand 2 | CCR2 chemokine receptor binding///CCR2 chemokine receptor binding///G-protein coupled receptor binding///chemokine activity///chemokine activity///cytokine activity///heparin binding///protein binding | G-protein coupled receptor signaling pathway///angiogenesis///cellular calcium ion homeostasis///cellular response to interferon-gamma///cellular response to interleukin-1///cellular response to lipopolysaccharide///cellular response to tumor necrosis factor///chemokine-mediated signaling pathway///chemokine-mediated signaling pathway///chemotaxis///cytokine-mediated signaling pathway///cytoskeleton organization///eosinophil chemotaxis///glial cell migration///helper T cell extravasation///immune response///inflammatory response///leukocyte migration involved in inflammatory response///lymphocyte chemotaxis///lymphocyte chemotaxis///macrophage chemotaxis///monocyte homeostasis///negative regulation of angiogenesis///neutrophil chemotaxis///neutrophil chemotaxis///positive regulation of ERK1 and ERK2 cascade///positive regulation of GTPase activity///positive regulation of T cell activation///positive regulation of apoptotic cell clearance///positive regulation of cell adhesion///positive regulation of cell-cell adhesion///positive regulation of cellular extravasation///positive regulation of collagen biosynthetic process///positive regulation of endothelial cell proliferation///positive regulation of immune complex clearance by monocytes and macrophages///positive regulation of inflammatory response///positive regulation of leukocyte mediated cytotoxicity///positive regulation of leukocyte migration///positive regulation of macrophage chemotaxis///positive regulation of macrophage chemotaxis///positive regulation of monocyte chemotaxis///positive regulation of monocyte chemotaxis///positive regulation of nitric-oxide synthase biosynthetic process///positive regulation of protein targeting to membrane///positive regulation of synaptic transmission///positive regulation of tumor necrosis factor production///regulation of cell shape///regulation of vascular endothelial growth factor production///response to heat///response to wounding///transforming growth factor beta receptor signaling pathway///vascular endothelial growth factor receptor signaling pathway | colocalizes_with C-fiber///axon terminus///cytoplasm///dendrite///endocytic vesicle///extracellular region///extracellular space///extracellular space///neuronal cell body///perikaryon///perinuclear region of cytoplasm///rough endoplasmic reticulum///synapse |
| 1443078_at | 0.00063 | 1.41E-06 | -1.38E+01 | 6.069612 | -4.25 | 6030439D06Rik | RIKEN cDNA 6030439D06 gene | | | |
| 1421911_at | 0.00063 | 1.41E-06 | 1.38E+01 | 6.067738 | 3.24 | Stat2 | signal transducer and activator of transcription 2 | DNA binding///identical protein binding///protein binding///signal transducer activity///transcription factor activity, sequence-specific DNA binding | defense response to virus///mitophagy in response to mitochondrial depolarization///positive regulation of defense response to virus by host///regulation of mitochondrial fission///regulation of protein phosphorylation///regulation of transcription, DNA-templated///signal transduction///transcription, DNA-templated///type I interferon signaling pathway///type I interferon signaling pathway///xenophagy | cytoplasm///cytoplasm///nucleus///plasma membrane |
| 1420659_at | 0.00063 | 1.42E-06 | 1.38E+01 | 6.061519 | 3.52 | Slamf6 | SLAM family member 6 | | T-helper 17 cell lineage commitment///adaptive immune response///immune system process///innate immune response///natural killer cell differentiation///natural killer cell proliferation///positive regulation of interferon-gamma production///positive regulation of interleukin-17 production///positive regulation of natural killer cell mediated cytotoxicity | extracellular exosome///integral component of membrane///integral component of plasma membrane///membrane///plasma membrane |
| 1445308_at | 0.00063 | 1.44E-06 | -1.38E+01 | 6.051582 | -3.21 | Tssc1 | tumor suppressing subtransferable candidate 1 | molecular_function | biological_process///positive regulation of dense core granule transport | cellular_component |
| 1437463_x_at | 0.000647 | 1.49E-06 | 1.37E+01 | 6.017802 | 3.06 | Tgfbi | transforming growth factor, beta induced | cell adhesion molecule binding///collagen binding///extracellular matrix binding///integrin binding | cell adhesion///cell proliferation///extracellular matrix organization | basement membrane///extracellular exosome///extracellular matrix///colocalizes_with extracellular matrix///extracellular region///extracellular space///extracellular space///proteinaceous extracellular matrix///trans-Golgi network |
| 1440156_s_at | 0.000676 | 1.62E-06 | -1.36E+01 | 5.942819 | -4.03 | Tox2 | TOX high mobility group box family member 2 | RNA polymerase II transcription factor binding | positive regulation of transcription from RNA polymerase II promoter | nucleoplasm///nucleus |
| 1450097_s_at | 0.000676 | 1.63E-06 | -1.36E+01 | 5.938024 | -2.17 | Gna12 | guanine nucleotide binding protein, alpha 12 | D5 dopamine receptor binding///D5 dopamine receptor binding///G-protein beta/gamma-subunit complex binding///G-protein coupled receptor binding///GTP binding///GTPase activity///GTPase activity///guanyl nucleotide binding///metal ion binding///nucleotide binding///signal transducer activity | G-protein coupled receptor signaling pathway///G-protein coupled receptor signaling pathway///Rho protein signal transduction///adenylate cyclase-modulating G-protein coupled receptor signaling pathway///cell differentiation///embryonic digit morphogenesis///in utero embryonic development///intracellular signal transduction///regulation of TOR signaling///regulation of TOR signaling///regulation of cell shape///regulation of fibroblast migration///regulation of proteasomal ubiquitin-dependent protein catabolic process///regulation of proteasomal ubiquitin-dependent protein catabolic process///signal transduction///small GTPase mediated signal transduction | brush border membrane///brush border membrane///focal adhesion///heterotrimeric G-protein complex///membrane |
| 1417605_s_at | 0.000676 | 1.63E-06 | -1.36E+01 | 5.934631 | -1.86 | Camk1 | calcium/calmodulin-dependent protein kinase I | ATP binding///calmodulin binding///calmodulin-dependent protein kinase activity///catalytic activity///kinase activity///nucleotide binding///protein binding///protein kinase activity///protein serine/threonine kinase activity///transferase activity | cell cycle///cell differentiation///metabolic process///multicellular organism development///negative regulation of protein binding///nervous system development///nucleocytoplasmic transport///nucleocytoplasmic transport///phosphorylation///positive regulation of dendritic spine development///positive regulation of muscle cell differentiation///positive regulation of neuron projection development///positive regulation of peptidyl-serine phosphorylation///positive regulation of protein acetylation///positive regulation of protein export from nucleus///positive regulation of protein export from nucleus///positive regulation of protein serine/threonine kinase activity///positive regulation of synapse structural plasticity///positive regulation of syncytium formation by plasma membrane fusion///positive regulation of transcription from RNA polymerase II promoter///protein phosphorylation///protein phosphorylation///protein phosphorylation///regulation of muscle cell differentiation///regulation of protein binding///regulation of protein localization///signal transduction | cytoplasm///nucleus |
| 1415871_at | 0.000676 | 1.64E-06 | 1.35E+01 | 5.929988 | 2.92 | Tgfbi | transforming growth factor, beta induced | cell adhesion molecule binding///collagen binding///extracellular matrix binding///integrin binding | cell adhesion///cell proliferation///extracellular matrix organization | basement membrane///extracellular exosome///extracellular matrix///colocalizes_with extracellular matrix///extracellular region///extracellular space///extracellular space///proteinaceous extracellular matrix///trans-Golgi network |
| 1451322_at | 0.000676 | 1.68E-06 | -1.35E+01 | 5.909295 | -5.08 | Cmbl | carboxymethylenebutenolidase-like (Pseudomonas) | T/G mismatch-specific endonuclease activity///hydrolase activity///molecular_function///nuclease activity///phosphoric ester hydrolase activity///retroviral 3' processing activity | biological_process | cytoplasm///extracellular exosome |
| 1422751_at | 0.000676 | 1.71E-06 | -1.35E+01 | 5.889184 | -2.49 | Tle1 | transducin-like enhancer of split 1 | RNA polymerase II transcription corepressor activity///chromatin binding///identical protein binding///protein binding///transcription corepressor activity///transcription factor binding | Wnt signaling pathway///negative regulation of I-kappaB kinase/NF-kappaB signaling///negative regulation of anoikis///negative regulation of transcription from RNA polymerase II promoter///negative regulation of transcription, DNA-templated///positive regulation of gene expression///regulation of transcription from RNA polymerase II promoter///regulation of transcription, DNA-templated///transcription, DNA-templated | cytoplasm///cytosol///nucleolus///nucleus///nucleus///transcription factor complex |
| 1417404_at | 0.000676 | 1.71E-06 | -1.35E+01 | 5.888185 | -2.23 | Elovl6 | ELOVL family member 6, elongation of long chain fatty acids (yeast) | 3-oxo-arachidoyl-CoA synthase activity///3-oxo-cerotoyl-CoA synthase activity///3-oxo-lignoceronyl-CoA synthase activity///fatty acid elongase activity///fatty acid elongase activity///transferase activity///transferase activity, transferring acyl groups other than amino-acyl groups | fatty acid biosynthetic process///fatty acid elongation///fatty acid elongation, monounsaturated fatty acid///fatty acid elongation, polyunsaturated fatty acid///fatty acid elongation, saturated fatty acid///fatty acid elongation, saturated fatty acid///fatty acid metabolic process///lipid metabolic process///long-chain fatty acid biosynthetic process///sphingolipid biosynthetic process///very long-chain fatty acid biosynthetic process | endoplasmic reticulum///integral component of endoplasmic reticulum membrane///integral component of membrane///membrane |
| 1416016_at | 0.000676 | 1.72E-06 | 1.35E+01 | 5.884235 | 3.24 | Tap1 | transporter 1, ATP-binding cassette, sub-family B (MDR/TAP) | ADP binding///ATP binding///ATPase activity///ATPase activity, coupled to transmembrane movement of substances///MHC class I protein binding///MHC class I protein binding///MHC class Ib protein binding///MHC protein binding///TAP1 binding///TAP2 binding///nucleotide binding///contributes_to peptide antigen binding///peptide antigen-transporting ATPase activity///peptide transporter activity///contributes_to peptide transporter activity///peptide-transporting ATPase activity///protein heterodimerization activity///protein homodimerization activity///tapasin binding | adaptive immune response///antigen processing and presentation of endogenous peptide antigen via MHC class I///antigen processing and presentation of endogenous peptide antigen via MHC class I via ER pathway, TAP-dependent///antigen processing and presentation of endogenous peptide antigen via MHC class Ib via ER pathway, TAP-dependent///antigen processing and presentation of exogenous protein antigen via MHC class Ib, TAP-dependent///cytosol to ER transport///defense response///immune system process///intracellular transport of viral protein in host cell///peptide transport///positive regulation of antigen processing and presentation of peptide antigen via MHC class I///protection from natural killer cell mediated cytotoxicity///protein transport///transmembrane transport///transport | TAP complex///endoplasmic reticulum///integral component of endoplasmic reticulum membrane///integral component of membrane///integral component of membrane///intracellular membrane-bounded organelle///membrane///mitochondrion |
| 1450378_at | 0.000676 | 1.75E-06 | 1.34E+01 | 5.867658 | 2.01 | Tapbp | TAP binding protein | TAP1 binding///TAP2 binding///protein binding | antigen processing and presentation of endogenous peptide antigen via MHC class I///antigen processing and presentation of exogenous peptide antigen via MHC class I, TAP-dependent///defense response///peptide antigen stabilization///regulation of gene expression///regulation of protein complex stability | Golgi membrane///MHC class I peptide loading complex///colocalizes_with TAP complex///endoplasmic reticulum///endoplasmic reticulum membrane///integral component of membrane///integral component of plasma membrane///membrane |
| 1416381_a_at | 0.000676 | 1.75E-06 | 1.34E+01 | 5.867583 | 1.9 | Prdx5 | peroxiredoxin 5 | RNA polymerase III regulatory region DNA binding///antioxidant activity///cysteine-type endopeptidase inhibitor activity involved in apoptotic process///oxidoreductase activity///peroxidase activity///peroxiredoxin activity///protein dimerization activity///receptor binding///receptor binding///thioredoxin peroxidase activity | cell redox homeostasis///cellular response to reactive oxygen species///hydrogen peroxide catabolic process///negative regulation of apoptotic process///negative regulation of transcription from RNA polymerase III promoter///oxidation-reduction process///response to oxidative stress///response to oxidative stress | cytoplasm///cytoplasm///cytoplasmic vesicle///cytosol///extracellular exosome///extracellular space///intracellular membrane-bounded organelle///mitochondrion///mitochondrion///nucleus///perinuclear region of cytoplasm///peroxisomal matrix///peroxisome |
| 1440920_at | 0.000676 | 1.76E-06 | 1.34E+01 | 5.86142 | 4.58 | Mmp14 | matrix metallopeptidase 14 (membrane-inserted) | calcium ion binding///hydrolase activity///integrin binding///metal ion binding///metalloendopeptidase activity///metallopeptidase activity///peptidase activator activity///peptidase activity///zinc ion binding | bone development///branching morphogenesis of an epithelial tube///cell migration///chondrocyte proliferation///collagen catabolic process///craniofacial suture morphogenesis///embryonic cranial skeleton morphogenesis///endochondral ossification///lung development///negative regulation of Notch signaling pathway///negative regulation of focal adhesion assembly///ossification///positive regulation of B cell differentiation///positive regulation of cell growth///positive regulation of cell migration///positive regulation of myotube differentiation///proteolysis///response to hormone///tissue remodeling///zymogen activation | cytoplasm///colocalizes_with cytoplasm///cytoplasmic vesicle///extracellular matrix///focal adhesion///integral component of membrane///macropinosome///membrane///plasma membrane |
| 1450136_at | 0.000676 | 1.77E-06 | 1.34E+01 | 5.855604 | 6.99 | Cd38 | CD38 antigen | NAD(P)+ nucleosidase activity///NAD+ nucleosidase activity///NAD+ nucleosidase activity///hydrolase activity///hydrolase activity, acting on glycosyl bonds///phosphorus-oxygen lyase activity///transferase activity | B cell receptor signaling pathway///long term synaptic depression///negative regulation of apoptotic process///negative regulation of bone resorption///negative regulation of transcription, DNA-templated///positive regulation of B cell proliferation///positive regulation of B cell proliferation///positive regulation of cell growth///positive regulation of cytosolic calcium ion concentration///positive regulation of insulin secretion///positive regulation of transcription, DNA-templated///positive regulation of vasoconstriction///response to drug///response to hormone///response to hydroperoxide///response to hypoxia | cell surface///extracellular exosome///integral component of membrane///intracellular membrane-bounded organelle///membrane///nucleus///plasma membrane///plasma membrane |
| 1422160_at | 0.000676 | 1.78E-06 | 1.34E+01 | 5.851963 | 3.4 | H2-T24 | histocompatibility 2, T region locus 24 | peptide antigen binding///receptor binding | antigen processing and presentation of peptide antigen via MHC class I | plasma membrane |
| 1448407_at | 0.000676 | 1.78E-06 | -1.34E+01 | 5.851439 | -2.13 | Vsir | V-set immunoregulatory receptor | protein binding | BMP signaling pathway///negative regulation of CD4-positive, alpha-beta T cell proliferation///negative regulation of T cell cytokine production///positive regulation of BMP signaling pathway///positive regulation of BMP signaling pathway///positive regulation of stem cell differentiation///stem cell differentiation | cell-cell junction///external side of plasma membrane///integral component of membrane///membrane///plasma membrane |
| 1446957_s_at | 0.000676 | 1.80E-06 | 1.34E+01 | 5.842691 | 2.51 | N4bp1 | NEDD4 binding protein 1 | RNA binding///protein binding | cellular response to UV///cellular response to UV///negative regulation of proteasomal ubiquitin-dependent protein catabolic process///negative regulation of protein ubiquitination | PML body///nucleolus///nucleus |
| 1449009_at | 0.000676 | 1.81E-06 | 1.34E+01 | 5.834446 | 5.84 | Tgtp2///Tgtp1 | T cell specific GTPase 2///T cell specific GTPase 1 | molecular_function///GTP binding///GTPase activity | biological_process///cellular response to interferon-beta///response to interferon-alpha///response to interferon-gamma | cellular_component///cellular_component |
| 1452348_s_at | 0.000676 | 1.82E-06 | 1.34E+01 | 5.833051 | 2.68 | Mndal///Mnda///Ifi205///Ifi204 | myeloid nuclear differentiation antigen like///myeloid cell nuclear differentiation antigen///interferon activated gene 205///interferon activated gene 204 | RNA polymerase II core promoter proximal region sequence-specific DNA binding///core promoter binding///double-stranded DNA binding///poly(A) RNA binding///transcription factor binding///transcriptional repressor activity, RNA polymerase II core promoter proximal region sequence-specific binding///RNA polymerase II core promoter proximal region sequence-specific DNA binding///core promoter binding///double-stranded DNA binding///poly(A) RNA binding///protein binding///transcription factor binding///transcriptional repressor activity, RNA polymerase II core promoter proximal region sequence-specific binding///RNA polymerase II core promoter proximal region sequence-specific DNA binding///core promoter binding///double-stranded DNA binding///double-stranded DNA binding///poly(A) RNA binding///transcription factor binding///transcriptional repressor activity, RNA polymerase II core promoter proximal region sequence-specific binding///RNA polymerase II core promoter proximal region sequence-specific DNA binding///core promoter binding///double-stranded DNA binding///double-stranded DNA binding///poly(A) RNA binding///protein binding///transcription cofactor activity///transcription factor binding///transcriptional repressor activity, RNA polymerase II core promoter proximal region sequence-specific binding | negative regulation of cell growth///regulation of growth///cellular response to interferon-beta///regulation of transcription, DNA-templated///transcription, DNA-templated///cellular response to interferon-alpha///cellular response to interferon-beta///inner ear development///intrinsic apoptotic signaling pathway in response to DNA damage by p53 class mediator///positive regulation of macrophage activation///positive regulation of osteoblast differentiation///regulation of transcription from RNA polymerase II promoter | cytoplasm///membrane///nucleolus///nucleus///nucleus///cytoplasm///membrane///nuclear periphery///nuclear speck///nucleolus///nucleus///cytoplasm///membrane///nucleolus///nucleus///cytoplasm///membrane///nuclear inclusion body///nuclear speck///nucleolus///nucleolus///nucleoplasm///nucleus |
| 1445639_at | 0.000676 | 1.83E-06 | 1.33E+01 | 5.826422 | 4.94 | Adgb | androglobin | calcium-dependent cysteine-type endopeptidase activity///heme binding///oxygen binding | proteolysis | cytoplasm///intracellular |
| 1422526_at | 0.000678 | 1.85E-06 | 1.33E+01 | 5.81688 | 5.3 | Acsl1 | acyl-CoA synthetase long-chain family member 1 | ATP binding///acetate-CoA ligase (ADP-forming) activity///catalytic activity///decanoate--CoA ligase activity///ligase activity///long-chain fatty acid-CoA ligase activity///nucleotide binding | adiponectin-activated signaling pathway///fatty acid metabolic process///fatty acid transport///lipid biosynthetic process///lipid metabolic process///long-chain fatty acid import///long-chain fatty acid metabolic process///metabolic process///positive regulation of protein serine/threonine kinase activity///response to organic substance///triglyceride metabolic process///xenobiotic catabolic process | endoplasmic reticulum///integral component of membrane///intracellular membrane-bounded organelle///membrane///mitochondrial outer membrane///mitochondrion///mitochondrion///peroxisomal membrane///peroxisome///plasma membrane |
| 1416868_at | 0.00068 | 1.88E-06 | -1.33E+01 | 5.798652 | -2.5 | Cdkn2c | cyclin-dependent kinase inhibitor 2C (p18, inhibits CDK4) | cyclin-dependent protein serine/threonine kinase inhibitor activity///cyclin-dependent protein serine/threonine kinase inhibitor activity///protein kinase binding///protein kinase binding | G1/S transition of mitotic cell cycle///cell cycle///cell cycle arrest///negative regulation of cell growth///negative regulation of cell proliferation///negative regulation of cell proliferation///negative regulation of cyclin-dependent protein serine/threonine kinase activity///negative regulation of phosphorylation///regulation of cyclin-dependent protein serine/threonine kinase activity | cytoplasm///nucleus |
| 1460364_at | 0.00068 | 1.90E-06 | -1.33E+01 | 5.791928 | -3.17 | Gtf2ird1 | general transcription factor II I repeat domain-containing 1 | DNA binding///RNA polymerase II transcription factor activity, sequence-specific DNA binding///transcription factor activity, sequence-specific DNA binding | embryo development///multicellular organism development///regulation of transcription, DNA-templated///transcription, DNA-templated///transition between slow and fast fiber | cytoplasm///cytoplasm///nucleoplasm///nucleoplasm///nucleus |
| 1433564_at | 0.00068 | 1.91E-06 | -1.33E+01 | 5.786911 | -1.85 | Dgkd | diacylglycerol kinase, delta | diacylglycerol kinase activity///protein heterodimerization activity///protein homodimerization activity | diacylglycerol metabolic process///protein homooligomerization///response to organic substance | cytoplasm///cytoplasmic, membrane-bounded vesicle///plasma membrane |
| 1434437_x_at | 0.00068 | 1.92E-06 | -1.33E+01 | 5.783493 | -3.55 | Rrm2 | ribonucleotide reductase M2 | metal ion binding///oxidoreductase activity///protein binding///ribonucleoside-diphosphate reductase activity, thioredoxin disulfide as acceptor///contributes_to ribonucleoside-diphosphate reductase activity, thioredoxin disulfide as acceptor///contributes_to ribonucleoside-diphosphate reductase activity, thioredoxin disulfide as acceptor | DNA replication///deoxyribonucleoside diphosphate metabolic process///deoxyribonucleotide biosynthetic process///deoxyribonucleotide metabolic process///oxidation-reduction process///protein heterotetramerization///protein oligomerization | cytoplasm///nuclear envelope///nucleus///ribonucleoside-diphosphate reductase complex |
| 1432026_a_at | 0.00068 | 1.93E-06 | 1.32E+01 | 5.776043 | 3.79 | Herc6 | hect domain and RLD 6 | UDP-N-acetylmuramoylalanyl-D-glutamyl-2,6-diaminopimelate-D-alanyl-D-alanine ligase activity///coenzyme F420-0 gamma-glutamyl ligase activity///coenzyme F420-2 alpha-glutamyl ligase activity///cyclin binding///ligase activity///protein-glutamic acid ligase activity///protein-glycine ligase activity///protein-glycine ligase activity, elongating///protein-glycine ligase activity, initiating///ribosomal S6-glutamic acid ligase activity///tubulin-glutamic acid ligase activity///tubulin-glycine ligase activity///ubiquitin protein ligase activity///ubiquitin-protein transferase activity///ubiquitin-protein transferase activity | hematopoietic progenitor cell differentiation///immune system process///innate immune response///protein ubiquitination///protein ubiquitination involved in ubiquitin-dependent protein catabolic process | cytoplasm///cytoplasm///nucleus///nucleus |
| 1440481_at | 0.00068 | 1.96E-06 | 1.32E+01 | 5.763896 | 3.04 | Stat1 | signal transducer and activator of transcription 1 | CCR5 chemokine receptor binding///DNA binding///RNA polymerase II core promoter proximal region sequence-specific DNA binding///RNA polymerase II core promoter sequence-specific DNA binding///cadherin binding involved in cell-cell adhesion///double-stranded DNA binding///enzyme binding///identical protein binding///nuclear hormone receptor binding///protein binding///protein homodimerization activity///protein phosphatase 2A binding///sequence-specific DNA binding///signal transducer activity///transcription factor activity, RNA polymerase II core promoter sequence-specific///transcription factor activity, sequence-specific DNA binding///transcription factor activity, sequence-specific DNA binding///tumor necrosis factor receptor binding | JAK-STAT cascade///activation of cysteine-type endopeptidase activity involved in apoptotic process///blood circulation///cell-cell adhesion///cellular response to cytokine stimulus///cellular response to insulin stimulus///cellular response to interferon-beta///cellular response to lipopolysaccharide///cellular response to organic cyclic compound///cytokine-mediated signaling pathway///interferon-gamma-mediated signaling pathway///interferon-gamma-mediated signaling pathway///lipopolysaccharide-mediated signaling pathway///macrophage derived foam cell differentiation///negative regulation by virus of viral protein levels in host cell///negative regulation of I-kappaB kinase/NF-kappaB signaling///negative regulation of angiogenesis///negative regulation of endothelial cell proliferation///negative regulation of macrophage fusion///positive regulation of cell proliferation///positive regulation of smooth muscle cell proliferation///positive regulation of transcription from RNA polymerase II promoter///positive regulation of transcription, DNA-templated///regulation of transcription, DNA-templated///renal tubule development///response to bacterium///response to cAMP///response to cytokine///response to drug///response to exogenous dsRNA///response to interferon-beta///response to lipopolysaccharide///response to mechanical stimulus///response to nutrient///response to peptide hormone///response to type I interferon///signal transduction///transcription, DNA-templated///tumor necrosis factor-mediated signaling pathway///type I interferon signaling pathway | axon///cell-cell adherens junction///cytoplasm///cytoplasm///dendrite///nuclear chromatin///nucleolus///nucleoplasm///nucleus///nucleus///perinuclear region of cytoplasm |
| 1451611_at | 0.00068 | 1.96E-06 | 1.32E+01 | 5.761865 | 2.48 | Pla2g16 | phospholipase A2, group XVI | 1-acyl-2-lysophosphatidylserine acylhydrolase activity///hydrolase activity///phosphatidylcholine 1-acylhydrolase activity///phosphatidylserine 1-acylhydrolase activity///phospholipase A2 activity///phospholipase A2 activity///NOT transferase activity, transferring acyl groups | lipid catabolic process///lipid metabolic process///negative regulation of cell cycle///phospholipid biosynthetic process///phospholipid metabolic process | cytoplasm///endoplasmic reticulum///integral component of membrane///membrane///perinuclear region of cytoplasm |
| 1424542_at | 0.00068 | 1.98E-06 | -1.32E+01 | 5.754329 | -2.89 | S100a4 | S100 calcium binding protein A4 | RAGE receptor binding///actin binding///calcium ion binding///calcium ion binding///calcium-dependent protein binding///identical protein binding///metal ion binding///poly(A) RNA binding///protein binding | positive regulation of I-kappaB kinase/NF-kappaB signaling | extracellular exosome///extracellular space///neuron projection///nucleus///perinuclear region of cytoplasm |
| 1426133_a_at | 0.000682 | 2.00E-06 | 1.32E+01 | 5.743563 | 2.17 | Mitd1 | MIT, microtubule interacting and transport, domain containing 1 | phosphatidylinositol binding///protein domain specific binding///protein homodimerization activity | cell cycle///cell division///cell separation after cytokinesis///mitotic cytokinesis///negative regulation of protein binding///transport | endosome///extracellular exosome///extrinsic component of membrane///intracellular membrane-bounded organelle///membrane///midbody///colocalizes_with midbody |
| 1420501_at | 0.000682 | 2.01E-06 | 1.32E+01 | 5.737245 | 2.69 | Dnajc1 | DnaJ heat shock protein family (Hsp40) member C1 | DNA binding///chaperone binding///protein binding///ribosome binding | protein folding///regulation of protein secretion///regulation of translation | cytosol///endoplasmic reticulum///endoplasmic reticulum lumen///integral component of membrane///intracellular membrane-bounded organelle///membrane///nucleus///plasma membrane |
| 1419697_at | 0.000682 | 2.03E-06 | 1.32E+01 | 5.730318 | 7.37 | Cxcl11 | chemokine (C-X-C motif) ligand 11 | | | |
| 1417632_at | 0.000682 | 2.04E-06 | -1.31E+01 | 5.722802 | -2.52 | Atp6v0a1 | ATPase, H+ transporting, lysosomal V0 subunit A1 | ATPase binding///ATPase binding///hydrogen ion transmembrane transporter activity///protein binding///proton-transporting ATPase activity, rotational mechanism | ATP hydrolysis coupled proton transport///ATP synthesis coupled proton transport///ion transport///proton transport///regulation of macroautophagy///toxin transport///transport///vacuolar acidification///vacuolar proton-transporting V-type ATPase complex assembly | Golgi apparatus///cytoplasm///cytoplasm///cytoplasmic vesicle///extracellular exosome///integral component of membrane///intracellular membrane-bounded organelle///membrane///nucleoplasm///plasma membrane///proton-transporting V-type ATPase, V0 domain///terminal bouton///vacuolar proton-transporting V-type ATPase complex///vacuolar proton-transporting V-type ATPase, V0 domain |
| 1451426_at | 0.000708 | 2.13E-06 | 1.31E+01 | 5.681152 | 3.05 | Dhx58 | DEXH (Asp-Glu-X-His) box polypeptide 58 | ATP binding///DNA binding///RNA binding///double-stranded RNA binding///double-stranded RNA binding///helicase activity///hydrolase activity///hydrolase activity, acting on acid anhydrides///metal ion binding///nucleotide binding///single-stranded RNA binding///zinc ion binding | defense response to virus///immune system process///innate immune response///negative regulation of MDA-5 signaling pathway///negative regulation of RIG-I signaling pathway///negative regulation of RIG-I signaling pathway///negative regulation of innate immune response///negative regulation of innate immune response///negative regulation of type I interferon production///negative regulation of type I interferon production///positive regulation of MDA-5 signaling pathway///positive regulation of RIG-I signaling pathway///positive regulation of type I interferon production///regulation of innate immune response///response to virus | cytoplasm |
| 1435695_a_at | 0.000716 | 2.18E-06 | 1.30E+01 | 5.662546 | 3.08 | Ggct | gamma-glutamyl cyclotransferase | gamma-glutamylcyclotransferase activity///protein homodimerization activity///transferase activity///transferase activity, transferring acyl groups | glutathione biosynthetic process///release of cytochrome c from mitochondria | cytosol///extracellular exosome |
| 1456251_x_at | 0.000728 | 2.23E-06 | 1.30E+01 | 5.640041 | 2.26 | Tspo | translocator protein | androgen binding///benzodiazepine receptor activity///ion channel binding | behavioral response to pain///chloride transport///contact inhibition///establishment of protein localization to mitochondrion///glial cell migration///ion transport///lipid transport///maintenance of protein location in mitochondrion///negative regulation of ATP metabolic process///negative regulation of glial cell proliferation///negative regulation of mitochondrion organization///negative regulation of mitophagy///negative regulation of nitric oxide biosynthetic process///negative regulation of protein ubiquitination///negative regulation of tumor necrosis factor production///positive regulation of apoptotic process///positive regulation of calcium ion transport///positive regulation of glial cell proliferation///positive regulation of mitochondrial depolarization///positive regulation of necrotic cell death///positive regulation of reactive oxygen species metabolic process///positive regulation of reactive oxygen species metabolic process///regulation of steroid biosynthetic process///steroid biosynthetic process///transport | cytoplasm///extracellular exosome///integral component of membrane///intracellular membrane-bounded organelle///membrane///mitochondrial outer membrane///mitochondrion///mitochondrion///nuclear membrane |
| 1437405_a_at | 0.000747 | 2.30E-06 | -1.29E+01 | 5.608538 | -4.82 | Igfbp4 | insulin-like growth factor binding protein 4 | growth factor binding///insulin-like growth factor I binding///insulin-like growth factor II binding///insulin-like growth factor binding | positive regulation of MAPK cascade///positive regulation of insulin-like growth factor receptor signaling pathway///regulation of cell growth///regulation of glucose metabolic process///regulation of growth///type B pancreatic cell proliferation | extracellular region///extracellular space |
| 1419153_at | 0.000756 | 2.35E-06 | -1.29E+01 | 5.5913 | -3.22 | 2810417H13Rik | RIKEN cDNA 2810417H13 gene | chromatin binding///chromatin binding | DNA repair///DNA replication///cellular response to DNA damage stimulus///centrosome organization///regulation of cell cycle///response to UV///translesion synthesis///translesion synthesis | cytoplasm///nucleus///nucleus///perinuclear region of cytoplasm |
| 1419042_at | 0.000756 | 2.36E-06 | 1.29E+01 | 5.584307 | 6.28 | Iigp1 | interferon inducible GTPase 1 | GDP binding///GTP binding///GTPase activity///GTPase activity///hydrolase activity///identical protein binding///nucleotide binding | cellular response to interferon-beta///cytokine-mediated signaling pathway///defense response to Gram-negative bacterium///defense response to protozoan///immune system process///innate immune response///regulation of autophagy | Golgi apparatus///cytoplasm///endoplasmic reticulum///endoplasmic reticulum membrane///membrane///nucleus///symbiont-containing vacuole membrane |
| 1448632_at | 0.000768 | 2.43E-06 | 1.28E+01 | 5.559137 | 1.77 | Psmb10 | proteasome (prosome, macropain) subunit, beta type 10 | endopeptidase activity///hydrolase activity///peptidase activity///threonine-type endopeptidase activity | T cell proliferation///cell morphogenesis///proteolysis///proteolysis involved in cellular protein catabolic process | cytoplasm///nucleus///proteasome complex///proteasome core complex///spermatoproteasome complex |
| 1419604_at | 0.000768 | 2.44E-06 | 1.28E+01 | 5.551744 | 5.51 | Zbp1 | Z-DNA binding protein 1 | DNA binding///DNA binding///RNA binding///double-stranded RNA adenosine deaminase activity///left-handed Z-DNA binding | defense response to virus///immune system process///innate immune response///positive regulation of type I interferon-mediated signaling pathway///viral process | cytoplasm///cytosol///nucleus |
| 1421456_at | 0.000768 | 2.45E-06 | -1.28E+01 | 5.548323 | -4.14 | P2ry1 | purinergic receptor P2Y, G-protein coupled 1 | A1 adenosine receptor binding///ADP binding///ADP-activated adenosine receptor activity///ATP binding///ATP-activated adenosine receptor activity///G-protein coupled purinergic nucleotide receptor activity///G-protein coupled receptor activity///protein heterodimerization activity///scaffold protein binding///signal transducer activity | G-protein coupled purinergic nucleotide receptor signaling pathway///G-protein coupled receptor signaling pathway///adenosine receptor signaling pathway///adenylate cyclase-inhibiting G-protein coupled receptor signaling pathway///cellular response to organic cyclic compound///eating behavior///glial cell migration///negative regulation of binding///negative regulation of norepinephrine secretion///phospholipase C-activating G-protein coupled receptor signaling pathway///platelet activation///positive regulation of ERK1 and ERK2 cascade///positive regulation of cytosolic calcium ion concentration///positive regulation of hormone secretion///positive regulation of inositol trisphosphate biosynthetic process///positive regulation of ion transport///positive regulation of ion transport///positive regulation of penile erection///positive regulation of protein phosphorylation///positive regulation of transcription from RNA polymerase II promoter///protein localization to plasma membrane///regulation of receptor activity///regulation of vasodilation///relaxation of muscle///response to mechanical stimulus///sensory perception of pain///signal transduction///signal transduction involved in regulation of gene expression | apical plasma membrane///basolateral plasma membrane///cell body///cell surface///dendrite///integral component of membrane///membrane///colocalizes_with mitochondrion///plasma membrane///plasma membrane///postsynaptic density///postsynaptic membrane |
| 1417707_at | 0.000771 | 2.50E-06 | -1.28E+01 | 5.531022 | -2.35 | N4bp2l1 | NEDD4 binding protein 2-like 1 | molecular_function | biological_process | cellular_component |
| 1418825_at | 0.000771 | 2.51E-06 | 1.28E+01 | 5.528325 | 2.74 | Irgm1 | immunity-related GTPase family M member 1 | GTP binding///hydrolase activity///nucleotide binding | autophagy///defense response///immune system process///innate immune response | Golgi apparatus///cell projection///cytoplasmic vesicle///endoplasmic reticulum///endosome///late endosome///lysosome///membrane///plasma membrane |
| 1435903_at | 0.000771 | 2.53E-06 | -1.28E+01 | 5.518449 | -3.76 | Cd300a | CD300A molecule | phosphatase binding///phosphatidylethanolamine binding///phosphatidylserine binding///protein binding///signaling receptor activity | immune system process///negative regulation of B cell proliferation///negative regulation of B cell receptor signaling pathway///negative regulation of MAP kinase activity///negative regulation of NK T cell activation///negative regulation of activation of JAK2 kinase activity///negative regulation of eosinophil activation///negative regulation of eosinophil migration///negative regulation of fibroblast proliferation///negative regulation of mast cell activation involved in immune response///negative regulation of mast cell degranulation///negative regulation of mast cell degranulation///negative regulation of neutrophil activation///negative regulation of phagocytosis, engulfment///negative regulation of serotonin secretion///positive regulation of phosphoprotein phosphatase activity///receptor-mediated endocytosis///regulation of T cell receptor signaling pathway///serotonin secretion by mast cell | extracellular exosome///integral component of membrane///membrane///plasma membrane |
| 1422637_at | 0.000771 | 2.55E-06 | -1.27E+01 | 5.511737 | -2.02 | Rassf5 | Ras association (RalGDS/AF-6) domain family member 5 | Ras GTPase binding///metal ion binding///protein binding | apoptotic process///intracellular signal transduction///negative regulation of cell proliferation///positive regulation of protein ubiquitination///regulation of apoptotic process///regulation of protein localization to nucleus///signal transduction | cytoplasm///cytoskeleton///microtubule///nucleus |
| 1428909_at | 0.000771 | 2.57E-06 | 1.27E+01 | 5.50561 | 1.97 | A130040M12Rik | RIKEN cDNA A130040M12 gene | | | |
| 1418555_x_at | 0.000771 | 2.57E-06 | 1.27E+01 | 5.502572 | 4.54 | Spic | Spi-C transcription factor (Spi-1/PU.1 related) | DNA binding///RNA polymerase II transcription factor activity, sequence-specific DNA binding///sequence-specific DNA binding///transcription factor activity, sequence-specific DNA binding | blastocyst development///cell differentiation///positive regulation of transcription from RNA polymerase II promoter///regulation of transcription, DNA-templated///transcription, DNA-templated | nucleus |
| 1417314_at | 0.000771 | 2.58E-06 | 1.27E+01 | 5.499818 | 7.84 | Cfb | complement factor B | hydrolase activity///peptidase activity///serine-type endopeptidase activity///serine-type peptidase activity | cell proliferation///complement activation///complement activation, alternative pathway///complement activation, alternative pathway///immune system process///innate immune response///proteolysis///response to thyroid hormone | blood microparticle///extracellular exosome///extracellular region///extracellular space |
| 1422771_at | 0.000806 | 2.74E-06 | 1.26E+01 | 5.442865 | 2.69 | Smad6 | SMAD family member 6 | DNA binding///I-SMAD binding///R-SMAD binding///RNA polymerase II core promoter proximal region sequence-specific DNA binding///chromatin binding///co-SMAD binding///identical protein binding///metal ion binding///protein binding///transcription factor activity, sequence-specific DNA binding///transcription regulatory region DNA binding///transforming growth factor beta receptor, inhibitory cytoplasmic mediator activity///type I activin receptor binding///type I transforming growth factor beta receptor binding///ubiquitin protein ligase binding | BMP signaling pathway///aorta development///cardiac vascular smooth muscle cell development///cell-substrate adhesion///coronary vasculature development///coronary vasculature morphogenesis///fat cell differentiation///heart valve development///immune response///mitral valve morphogenesis///negative regulation of BMP signaling pathway///negative regulation of SMAD protein complex assembly///negative regulation of apoptotic process///negative regulation of cell proliferation///negative regulation of ossification///negative regulation of pathway-restricted SMAD protein phosphorylation///negative regulation of transforming growth factor beta receptor signaling pathway///negative regulation of vasodilation///outflow tract septum morphogenesis///positive regulation of pri-miRNA transcription from RNA polymerase II promoter///positive regulation of vasoconstriction///pulmonary valve morphogenesis///regulation of transcription, DNA-templated///transcription, DNA-templated///transforming growth factor beta receptor signaling pathway///ureteric bud development///ventricular septum development///zygotic specification of dorsal/ventral axis | intracellular///nucleus///protein complex///transcription factor complex |
| 1436625_at | 0.000806 | 2.75E-06 | 1.26E+01 | 5.439767 | 2.16 | Fcgr1 | Fc receptor, IgG, high affinity I | IgG binding///IgG binding///IgG receptor activity///leukotriene receptor binding | antibody-dependent cellular cytotoxicity///antigen processing and presentation of exogenous antigen///antigen processing and presentation of exogenous peptide antigen via MHC class I///cell surface receptor signaling pathway///defense response to bacterium///immune system process///innate immune response///phagocytosis, engulfment///phagocytosis, recognition///positive regulation of phagocytosis///positive regulation of phagocytosis, engulfment///positive regulation of type III hypersensitivity///positive regulation of type IIa hypersensitivity///receptor-mediated endocytosis///regulation of immune response///response to bacterium | external side of plasma membrane///integral component of membrane///membrane///membrane raft///plasma membrane |
| 1435331_at | 0.000806 | 2.75E-06 | 1.26E+01 | 5.438302 | 4.53 | Pyhin1 | pyrin and HIN domain family, member 1 | RNA polymerase II core promoter proximal region sequence-specific DNA binding///core promoter binding///double-stranded DNA binding///poly(A) RNA binding///transcription factor binding///transcriptional repressor activity, RNA polymerase II core promoter proximal region sequence-specific binding | cellular response to interferon-beta | cytoplasm///membrane///nucleolus///nucleus |
| 1419589_at | 0.000807 | 2.79E-06 | -1.26E+01 | 5.424679 | -2.41 | Cd93 | CD93 antigen | calcium ion binding///carbohydrate binding///complement component C1q binding | cell adhesion///mitophagy in response to mitochondrial depolarization///positive regulation of defense response to virus by host///single organismal cell-cell adhesion///single organismal cell-cell adhesion///xenophagy | cell surface///cytoplasmic, membrane-bounded vesicle///extracellular matrix///integral component of membrane///membrane///plasma membrane///plasma membrane |
| 1419043_a_at | 0.000807 | 2.82E-06 | 1.26E+01 | 5.414261 | 5.57 | Iigp1 | interferon inducible GTPase 1 | GDP binding///GTP binding///GTPase activity///GTPase activity///hydrolase activity///identical protein binding///nucleotide binding | cellular response to interferon-beta///cytokine-mediated signaling pathway///defense response to Gram-negative bacterium///defense response to protozoan///immune system process///innate immune response///regulation of autophagy | Golgi apparatus///cytoplasm///endoplasmic reticulum///endoplasmic reticulum membrane///membrane///nucleus///symbiont-containing vacuole membrane |
| 1451567_a_at | 0.000807 | 2.82E-06 | 1.26E+01 | 5.413885 | 3.61 | LOC102641031///LOC100862473///Gm16340///Ifi203 | pyrin domain-containing protein 3///cell wall protein AWA1-like///interferon-activable protein 203-like///interferon activated gene 203 | RNA polymerase II core promoter proximal region sequence-specific DNA binding///core promoter binding///double-stranded DNA binding///poly(A) RNA binding///transcription factor binding///transcriptional repressor activity, RNA polymerase II core promoter proximal region sequence-specific binding | cellular response to interferon-beta | cytoplasm///membrane///nucleolus///nucleus |
| 1445597_s_at | 0.000807 | 2.84E-06 | 1.25E+01 | 5.408551 | 2.48 | Pla2g16 | phospholipase A2, group XVI | 1-acyl-2-lysophosphatidylserine acylhydrolase activity///hydrolase activity///phosphatidylcholine 1-acylhydrolase activity///phosphatidylserine 1-acylhydrolase activity///phospholipase A2 activity///phospholipase A2 activity///NOT transferase activity, transferring acyl groups | lipid catabolic process///lipid metabolic process///negative regulation of cell cycle///phospholipid biosynthetic process///phospholipid metabolic process | cytoplasm///endoplasmic reticulum///integral component of membrane///membrane///perinuclear region of cytoplasm |
| 1429012_at | 0.000807 | 2.85E-06 | -1.25E+01 | 5.406302 | -1.8 | Arhgef6 | Rac/Cdc42 guanine nucleotide exchange factor (GEF) 6 | Rho guanyl-nucleotide exchange factor activity///guanyl-nucleotide exchange factor activity///protein binding | intracellular signal transduction///lamellipodium assembly///regulation of Rho protein signal transduction | cell projection///cell-cell junction///lamellipodium |
| 1436689_a_at | 0.000809 | 2.88E-06 | -1.25E+01 | 5.394355 | -2.25 | Aldh9a1 | aldehyde dehydrogenase 9, subfamily A1 | 4-trimethylammoniobutyraldehyde dehydrogenase activity///4-trimethylammoniobutyraldehyde dehydrogenase activity///NAD binding///aldehyde dehydrogenase (NAD) activity///amine binding///aminobutyraldehyde dehydrogenase activity///aminobutyraldehyde dehydrogenase activity///oxidoreductase activity///oxidoreductase activity, acting on the aldehyde or oxo group of donors, NAD or NADP as acceptor///protein homodimerization activity | carnitine metabolic process///cellular aldehyde metabolic process///metabolic process///neurotransmitter biosynthetic process///oxidation-reduction process | cytoplasm///cytosol///cytosol///extracellular exosome///mitochondrion |
| 1431110_at | 0.000809 | 2.91E-06 | -1.25E+01 | 5.385052 | -3.36 | Plxdc2 | plexin domain containing 2 | | | extracellular exosome///integral component of membrane///membrane///plasma membrane |
| 1429570_at | 0.000809 | 2.91E-06 | 1.25E+01 | 5.384991 | 3.59 | Mlkl | mixed lineage kinase domain-like | ATP binding///ATP binding///nucleotide binding///protein complex binding///NOT protein kinase activity///protein kinase binding | necroptotic process///necroptotic process///programmed cell death///protein homotrimerization///protein phosphorylation | cytoplasm///membrane///plasma membrane |
| 1450332_s_at | 0.000809 | 2.92E-06 | -1.25E+01 | 5.380825 | -2.28 | Fmo5 | flavin containing monooxygenase 5 | N,N-dimethylaniline monooxygenase activity///N,N-dimethylaniline monooxygenase activity///NADP binding///flavin adenine dinucleotide binding///monooxygenase activity///oxidoreductase activity | drug metabolic process///oxidation-reduction process | cytoplasm///endoplasmic reticulum///integral component of membrane///intracellular membrane-bounded organelle///membrane |
| 1435456_at | 0.000823 | 3.01E-06 | -1.24E+01 | 5.352636 | -3.57 | Ttc28 | tetratricopeptide repeat domain 28 | molecular_function | cell cycle///cell division///mitotic nuclear division///regulation of mitotic cell cycle | centrosome///cytoplasm///cytoskeleton///midbody///mitotic spindle///mitotic spindle midzone///mitotic spindle pole |
| 1450643_s_at | 0.000823 | 3.02E-06 | 1.24E+01 | 5.348016 | 4.77 | Acsl1 | acyl-CoA synthetase long-chain family member 1 | ATP binding///acetate-CoA ligase (ADP-forming) activity///catalytic activity///decanoate--CoA ligase activity///ligase activity///long-chain fatty acid-CoA ligase activity///nucleotide binding | adiponectin-activated signaling pathway///fatty acid metabolic process///fatty acid transport///lipid biosynthetic process///lipid metabolic process///long-chain fatty acid import///long-chain fatty acid metabolic process///metabolic process///positive regulation of protein serine/threonine kinase activity///response to organic substance///triglyceride metabolic process///xenobiotic catabolic process | endoplasmic reticulum///integral component of membrane///intracellular membrane-bounded organelle///membrane///mitochondrial outer membrane///mitochondrion///mitochondrion///peroxisomal membrane///peroxisome///plasma membrane |
| 1417910_at | 0.000823 | 3.03E-06 | -1.24E+01 | 5.346496 | -3.95 | Ccna2 | cyclin A2 | protein binding///protein kinase binding | cell cycle///cell division///mitotic nuclear division///positive regulation of fibroblast proliferation///positive regulation of transcription, DNA-templated///positive regulation of transcription, DNA-templated///regulation of G2/M transition of mitotic cell cycle///regulation of cyclin-dependent protein serine/threonine kinase activity | Golgi apparatus///cyclin A2-CDK2 complex///cytoplasm///cytoplasm///female pronucleus///male pronucleus///nucleoplasm///nucleus///nucleus |
| 1418293_at | 0.000843 | 3.14E-06 | 1.24E+01 | 5.311624 | 4.53 | Ifit2 | interferon-induced protein with tetratricopeptide repeats 2 | RNA binding///poly(A) RNA binding | apoptotic process///cellular response to interferon-alpha///defense response to virus///immune system process///innate immune response///negative regulation of protein binding///positive regulation of apoptotic process///response to virus///response to virus | cytoplasm///endoplasmic reticulum |
| 1456250_x_at | 0.000843 | 3.15E-06 | 1.24E+01 | 5.309269 | 2.43 | Tgfbi | transforming growth factor, beta induced | cell adhesion molecule binding///collagen binding///extracellular matrix binding///integrin binding | cell adhesion///cell proliferation///extracellular matrix organization | basement membrane///extracellular exosome///extracellular matrix///colocalizes_with extracellular matrix///extracellular region///extracellular space///extracellular space///proteinaceous extracellular matrix///trans-Golgi network |
| 1445334_at | 0.000843 | 3.16E-06 | -1.24E+01 | 5.306384 | -1.64 | 1700047I17Rik2///Fam177a | RIKEN cDNA 1700047I17 gene 2///family with sequence similarity 177, member A | molecular_function///molecular_function | biological_process///biological_process | cellular_component///cellular_component |
| 1422300_at | 0.000866 | 3.27E-06 | 1.23E+01 | 5.27388 | 3.1 | Nog | noggin | cytokine binding///protein complex binding///protein homodimerization activity | BMP signaling pathway///anatomical structure formation involved in morphogenesis///anatomical structure formation involved in morphogenesis///axial mesoderm development///axon guidance///brain development///cartilage development///cell differentiation///cell differentiation in hindbrain///cellular response to BMP stimulus///central nervous system development///dorsal/ventral pattern formation///embryonic digit morphogenesis///embryonic skeletal joint morphogenesis///embryonic skeletal system development///endoderm formation///epithelial to mesenchymal transition///face morphogenesis///fibroblast growth factor receptor signaling pathway involved in neural plate anterior/posterior pattern formation///in utero embryonic development///limb development///lung morphogenesis///memory///mesenchymal cell differentiation///mesoderm formation///middle ear morphogenesis///motor neuron axon guidance///multicellular organism development///negative regulation of BMP signaling pathway///negative regulation of BMP signaling pathway///negative regulation of BMP signaling pathway///negative regulation of BMP signaling pathway///negative regulation of apoptotic signaling pathway///negative regulation of astrocyte differentiation///negative regulation of astrocyte differentiation///negative regulation of canonical Wnt signaling pathway///negative regulation of cardiac muscle cell proliferation///negative regulation of cartilage development///negative regulation of cell differentiation///negative regulation of cell migration///negative regulation of cytokine activity///negative regulation of gene expression///negative regulation of osteoblast differentiation///negative regulation of pathway-restricted SMAD protein phosphorylation///negative regulation of transcription from RNA polymerase II promoter///neural plate morphogenesis///neural tube closure///neural tube development///notochord morphogenesis///osteoblast differentiation///pattern specification process///pituitary gland development///positive regulation of branching involved in ureteric bud morphogenesis///positive regulation of cell proliferation///positive regulation of epithelial cell proliferation///positive regulation of epithelial cell proliferation///positive regulation of glomerulus development///positive regulation of oligodendrocyte progenitor proliferation///positive regulation of transcription from RNA polymerase II promoter///positive regulation of transcription from RNA polymerase II promoter///prostatic bud formation///regulation of BMP signaling pathway///regulation of fibroblast growth factor receptor signaling pathway involved in neural plate anterior/posterior pattern formation///skeletal system development///somatic stem cell population maintenance///somite development///spinal cord development///ureteric bud development///ureteric bud formation///urogenital system development///visual learning///wound healing | extracellular region///extracellular space///extracellular space///protein complex |
| 1426276_at | 0.000866 | 3.28E-06 | 1.23E+01 | 5.26911 | 2.46 | Ifih1 | interferon induced with helicase C domain 1 | ATP binding///DNA binding///RNA binding///double-stranded RNA binding///helicase activity///hydrolase activity///hydrolase activity, acting on acid anhydrides///metal ion binding///nucleotide binding///protein binding///ribonucleoprotein complex binding///single-stranded RNA binding///zinc ion binding | MDA-5 signaling pathway///defense response to virus///immune system process///innate immune response///positive regulation of interferon-alpha production///positive regulation of interferon-beta production///protein sumoylation///response to virus | cytoplasm///nucleus |
| 1432399_a_at | 0.000874 | 3.33E-06 | 1.23E+01 | 5.254703 | 4.35 | Epha1 | Eph receptor A1 | ATP binding///ephrin receptor activity///kinase activity///nucleotide binding///protein kinase activity///protein kinase binding///protein tyrosine kinase activity///transferase activity///transmembrane receptor protein tyrosine kinase activity///transmembrane-ephrin receptor activity | activation of GTPase activity///angiogenesis///cell adhesion///cell surface receptor signaling pathway///ephrin receptor signaling pathway///negative regulation of cell migration///negative regulation of protein kinase activity///peptidyl-tyrosine phosphorylation///phosphorylation///positive regulation of angiogenesis///positive regulation of cell migration///positive regulation of cell proliferation///positive regulation of cell-matrix adhesion///positive regulation of stress fiber assembly///protein autophosphorylation///protein phosphorylation///regulation of GTPase activity///substrate adhesion-dependent cell spreading///transmembrane receptor protein tyrosine kinase signaling pathway | integral component of membrane///integral component of plasma membrane///membrane///plasma membrane |
| 1419833_s_at | 0.00088 | 3.38E-06 | -1.22E+01 | 5.241694 | -3.64 | Arap3 | ArfGAP with RhoGAP domain, ankyrin repeat and PH domain 3 | GTPase activator activity///NOT GTPase activator activity///phosphatidylinositol-3,4,5-trisphosphate binding///phosphatidylinositol-3,4-bisphosphate binding///protein binding | negative regulation of Rac protein signal transduction///negative regulation of Rho protein signal transduction///negative regulation of cell migration///regulation of cell shape///signal transduction | cell projection///cytoplasm///cytoskeleton///lamellipodium///membrane///plasma membrane///ruffle |
| 1436080_at | 0.000906 | 3.50E-06 | 1.22E+01 | 5.207755 | 4.36 | AW011738 | expressed sequence AW011738 | | | |
| 1450424_a_at | 0.000907 | 3.52E-06 | 1.22E+01 | 5.202197 | 3.53 | Il18bp | interleukin 18 binding protein | interleukin-18 binding///interleukin-18 binding///receptor antagonist activity | T-helper 1 type immune response///T-helper 1 type immune response | extracellular exosome///extracellular region///extracellular space///extracellular space |
| 1434684_at | 0.000916 | 3.62E-06 | -1.21E+01 | 5.175032 | -2.36 | Rin3 | Ras and Rab interactor 3 | GTPase activator activity///Rab GTPase binding///Rab guanyl-nucleotide exchange factor activity | signal transduction | cytoplasm///cytoplasmic vesicle///early endosome///endosome |
| 1423704_at | 0.000916 | 3.63E-06 | -1.21E+01 | 5.172823 | -2.06 | Pla2g15 | phospholipase A2, group XV | 2,4,4-trimethyl-3-oxopentanoyl-CoA 2-C-propanoyl transferase activity///2,6-dimethyl-5-methylene-3-oxo-heptanoyl-CoA C-acetyltransferase activity///2-methylhexanoyl-CoA C-acetyltransferase activity///3-hydroxybutyryl-CoA thiolase activity///3-ketopimelyl-CoA thiolase activity///C-acyltransferase activity///C-palmitoyltransferase activity///L-2-aminoadipate N-acetyltransferase activity///N-acetyltransferase activity///N-acyltransferase activity///N-palmitoyltransferase activity///N-succinyltransferase activity///O-acetyltransferase activity///O-acyltransferase activity///O-acyltransferase activity///O-octanoyltransferase activity///O-palmitoyltransferase activity///O-sinapoyltransferase activity///O-succinyltransferase activity///Ras palmitoyltransferase activity///S-acetyltransferase activity///S-acyltransferase activity///S-malonyltransferase activity///S-succinyltransferase activity///acetyl-CoA:L-lysine N6-acetyltransferase///acetyltransferase activity///acyl-CoA N-acyltransferase activity///acylglycerol O-acyltransferase activity///azetidine-2-carboxylic acid acetyltransferase activity///benzoyl acetate-CoA thiolase activity///butyryl-CoA 2-C-propionyltransferase activity///calcium-independent phospholipase A2 activity///calcium-independent phospholipase A2 activity///carnitine O-acyltransferase activity///dihydrolipoamide S-acyltransferase activity///dihydrolipoamide branched chain acyltransferase activity///ergosterol O-acyltransferase activity///glucosaminyl-phosphotidylinositol O-acyltransferase activity///hydrolase activity///keto acid formate lyase activity///lanosterol O-acyltransferase activity///NOT lysophospholipase activity///malonyltransferase activity///myristoyltransferase activity///naphthyl-2-oxomethyl-succinyl-CoA succinyl transferase activity///octanoyltransferase activity///palmitoleoyl [acyl-carrier-protein]-dependent acyltransferase activity///palmitoyltransferase activity///peptidyl-lysine N-acetyltransferase activity, acting on acetyl phosphate as donor///peptidyl-lysine N6-myristoyltransferase activity///peptidyl-lysine N6-palmitoyltransferase activity///protein-cysteine S-acyltransferase activity///protein-cysteine S-myristoyltransferase activity///serine O-acyltransferase activity///sinapoyltransferase activity///sterol O-acyltransferase activity///succinyltransferase activity///transferase activity///transferase activity, transferring acyl groups | ceramide metabolic process///ceramide metabolic process///fatty acid metabolic process///glycerophospholipid metabolic process///glycerophospholipid metabolic process///lipid metabolic process///phosphatidylcholine catabolic process///phosphatidylcholine metabolic process///phosphatidylethanolamine catabolic process | extracellular exosome///extracellular region///extracellular space///extracellular space///lysosome///membrane///mitochondrion |
| 1440666_at | 0.000916 | 3.65E-06 | 1.21E+01 | 5.166949 | 3.91 | 4933430I17Rik | RIKEN cDNA 4933430I17 gene | molecular_function | biological_process | cellular_component |
| 1437152_at | 0.000916 | 3.65E-06 | -1.21E+01 | 5.166185 | -2.65 | Mex3b | mex3 RNA binding family member B | GTPase activating protein binding///RNA binding///metal ion binding///nucleic acid binding///poly(A) RNA binding///zinc ion binding | positive regulation of GTPase activity///positive regulation of cell-cell adhesion///positive regulation of phagocytosis///protein localization to cell cortex | cytoplasm///nucleus |
| 1426260_a_at | 0.000916 | 3.66E-06 | -1.21E+01 | 5.164483 | -1.83 | Ugt1a1///Ugt1a6b///Ugt1a9///Ugt1a5///Ugt1a7c///Ugt1a10///Ugt1a6a///Ugt1a2 | UDP glucuronosyltransferase 1 family, polypeptide A1///UDP glucuronosyltransferase 1 family, polypeptide A6B///UDP glucuronosyltransferase 1 family, polypeptide A9///UDP glucuronosyltransferase 1 family, polypeptide A5///UDP glucuronosyltransferase 1 family, polypeptide A7C///UDP glycosyltransferase 1 family, polypeptide A10///UDP glucuronosyltransferase 1 family, polypeptide A6A///UDP glucuronosyltransferase 1 family, polypeptide A2 | enzyme binding///enzyme inhibitor activity///glucuronosyltransferase activity///glucuronosyltransferase activity///glucuronosyltransferase activity///protein heterodimerization activity///protein homodimerization activity///retinoic acid binding///steroid binding///transferase activity///transferase activity, transferring glycosyl groups///transferase activity, transferring hexosyl groups///glucuronosyltransferase activity///glucuronosyltransferase activity///protein complex binding///protein heterodimerization activity///protein homodimerization activity///enzyme inhibitor activity///fatty acid binding///glucuronosyltransferase activity///glucuronosyltransferase activity///protein heterodimerization activity///protein homodimerization activity///protein kinase C binding///steroid binding///transferase activity///transferase activity, transferring glycosyl groups///transferase activity, transferring hexosyl groups///glucuronosyltransferase activity///glucuronosyltransferase activity///protein heterodimerization activity///protein homodimerization activity///glucuronosyltransferase activity///glucuronosyltransferase activity///transferase activity///transferase activity, transferring glycosyl groups///transferase activity, transferring hexosyl groups///enzyme inhibitor activity///fatty acid binding///glucuronosyltransferase activity///glucuronosyltransferase activity///protein heterodimerization activity///protein homodimerization activity///protein kinase C binding///steroid binding///glucuronosyltransferase activity///glucuronosyltransferase activity///protein complex binding///protein heterodimerization activity///protein homodimerization activity///transferase activity///transferase activity, transferring glycosyl groups///transferase activity, transferring hexosyl groups///glucuronosyltransferase activity///glucuronosyltransferase activity///protein heterodimerization activity///protein homodimerization activity///retinoic acid binding///transferase activity///transferase activity, transferring glycosyl groups///transferase activity, transferring hexosyl groups | biphenyl catabolic process///cellular glucuronidation///flavone metabolic process///flavonoid biosynthetic process///flavonoid glucuronidation///flavonoid glucuronidation///metabolic process///response to organic substance///xenobiotic glucuronidation///xenobiotic glucuronidation///flavonoid biosynthetic process///flavonoid glucuronidation///xenobiotic glucuronidation///flavonoid biosynthetic process///flavonoid glucuronidation///metabolic process///xenobiotic glucuronidation///cellular glucuronidation///flavonoid biosynthetic process///flavonoid glucuronidation///xenobiotic glucuronidation///aromatic compound catabolic process///coumarin catabolic process///estrogen catabolic process///flavonoid biosynthetic process///flavonoid glucuronidation///metabolic process///xenobiotic glucuronidation///flavonoid biosynthetic process///flavonoid glucuronidation///xenobiotic glucuronidation///flavonoid biosynthetic process///flavonoid glucuronidation///glucuronate metabolic process///glucuronate metabolic process///metabolic process///xenobiotic glucuronidation///cellular glucuronidation///flavonoid biosynthetic process///flavonoid glucuronidation///flavonoid glucuronidation///metabolic process///xenobiotic glucuronidation///xenobiotic glucuronidation | cytochrome complex///endoplasmic reticulum///endoplasmic reticulum chaperone complex///integral component of membrane///integral component of plasma membrane///intracellular membrane-bounded organelle///membrane///endoplasmic reticulum///extracellular exosome///intracellular membrane-bounded organelle///protein complex///endoplasmic reticulum///extracellular exosome///integral component of membrane///intracellular membrane-bounded organelle///membrane///endoplasmic reticulum///endoplasmic reticulum///integral component of membrane///intracellular membrane-bounded organelle///membrane///endoplasmic reticulum///extracellular exosome///membrane///endoplasmic reticulum///extracellular exosome///integral component of membrane///intracellular membrane-bounded organelle///membrane///mitochondrial inner membrane///protein complex///endoplasmic reticulum///integral component of membrane///intracellular membrane-bounded organelle///membrane |
| 1452178_at | 0.000924 | 3.73E-06 | 1.21E+01 | 5.146017 | 2.72 | Parp10///Plec | poly (ADP-ribose) polymerase family, member 10///plectin | K63-linked polyubiquitin binding///NAD+ ADP-ribosyltransferase activity///actin binding///ankyrin binding///cadherin binding involved in cell-cell adhesion///cytoskeletal protein binding///poly(A) RNA binding///protein N-terminus binding///protein binding///structural constituent of cytoskeleton | negative regulation of NF-kappaB transcription factor activity///negative regulation of fibroblast proliferation///negative regulation of gene expression///negative regulation of protein K63-linked ubiquitination///negative regulation of protein import into nucleus, translocation///negative regulation of viral genome replication///protein ADP-ribosylation///protein auto-ADP-ribosylation///protein poly-ADP-ribosylation///regulation of chromatin assembly///hemidesmosome assembly///response to nutrient | Golgi apparatus///cytoplasm///nucleoplasm///nucleus///apical plasma membrane///basal plasma membrane///brush border///cell junction///cell-cell adherens junction///contractile fiber///cytoplasm///cytoskeleton///extracellular exosome///extracellular matrix///focal adhesion///hemidesmosome///intermediate filament cytoskeleton///perinuclear region of cytoplasm///sarcolemma///sarcoplasm |
| 1416239_at | 0.000924 | 3.74E-06 | 1.21E+01 | 5.143115 | 8.19 | Gm5424///Ass1 | argininosuccinate synthase pseudogene///argininosuccinate synthetase 1 | ATP binding///amino acid binding///argininosuccinate synthase activity///argininosuccinate synthase activity///identical protein binding///ligase activity///nucleotide binding///poly(A) RNA binding///toxic substance binding | arginine biosynthetic process///arginine biosynthetic process///argininosuccinate metabolic process///argininosuccinate metabolic process///aspartate metabolic process///cellular amino acid biosynthetic process///cellular response to laminar fluid shear stress///citrulline metabolic process///negative regulation of leukocyte cell-cell adhesion///positive regulation of nitric oxide biosynthetic process///response to glucocorticoid///response to mycotoxin///response to zinc ion///urea cycle///urea cycle | cell body fiber///cytoplasm///endoplasmic reticulum///extracellular exosome///lysosome///mitochondrial outer membrane///mitochondrion///myelin sheath///neuron projection///neuronal cell body///nucleus///perikaryon |
| 1420915_at | 0.000924 | 3.75E-06 | 1.21E+01 | 5.140188 | 2.8 | Stat1 | signal transducer and activator of transcription 1 | CCR5 chemokine receptor binding///DNA binding///RNA polymerase II core promoter proximal region sequence-specific DNA binding///RNA polymerase II core promoter sequence-specific DNA binding///cadherin binding involved in cell-cell adhesion///double-stranded DNA binding///enzyme binding///identical protein binding///nuclear hormone receptor binding///protein binding///protein homodimerization activity///protein phosphatase 2A binding///sequence-specific DNA binding///signal transducer activity///transcription factor activity, RNA polymerase II core promoter sequence-specific///transcription factor activity, sequence-specific DNA binding///transcription factor activity, sequence-specific DNA binding///tumor necrosis factor receptor binding | JAK-STAT cascade///activation of cysteine-type endopeptidase activity involved in apoptotic process///blood circulation///cell-cell adhesion///cellular response to cytokine stimulus///cellular response to insulin stimulus///cellular response to interferon-beta///cellular response to lipopolysaccharide///cellular response to organic cyclic compound///cytokine-mediated signaling pathway///interferon-gamma-mediated signaling pathway///interferon-gamma-mediated signaling pathway///lipopolysaccharide-mediated signaling pathway///macrophage derived foam cell differentiation///negative regulation by virus of viral protein levels in host cell///negative regulation of I-kappaB kinase/NF-kappaB signaling///negative regulation of angiogenesis///negative regulation of endothelial cell proliferation///negative regulation of macrophage fusion///positive regulation of cell proliferation///positive regulation of smooth muscle cell proliferation///positive regulation of transcription from RNA polymerase II promoter///positive regulation of transcription, DNA-templated///regulation of transcription, DNA-templated///renal tubule development///response to bacterium///response to cAMP///response to cytokine///response to drug///response to exogenous dsRNA///response to interferon-beta///response to lipopolysaccharide///response to mechanical stimulus///response to nutrient///response to peptide hormone///response to type I interferon///signal transduction///transcription, DNA-templated///tumor necrosis factor-mediated signaling pathway///type I interferon signaling pathway | axon///cell-cell adherens junction///cytoplasm///cytoplasm///dendrite///nuclear chromatin///nucleolus///nucleoplasm///nucleus///nucleus///perinuclear region of cytoplasm |
| 1454942_at | 0.00093 | 3.80E-06 | -1.20E+01 | 5.128532 | -3.71 | Fam129a | family with sequence similarity 129, member A | molecular_function | negative regulation of protein phosphorylation///positive regulation of protein phosphorylation///positive regulation of translation///regulation of translation///response to endoplasmic reticulum stress | cytoplasm///extracellular exosome///membrane///plasma membrane |
| 1418872_at | 0.000936 | 3.85E-06 | -1.20E+01 | 5.11586 | -3.24 | Abcb1b | ATP-binding cassette, sub-family B (MDR/TAP), member 1B | ATP binding///ATPase activity///ATPase activity, coupled to transmembrane movement of substances///drug transmembrane transporter activity///hydrolase activity///nucleotide binding///transporter activity///xenobiotic-transporting ATPase activity | antigen processing and presentation of endogenous peptide antigen via MHC class I via ER pathway, TAP-dependent///antigen processing and presentation of endogenous peptide antigen via MHC class Ib via ER pathway, TAP-dependent///antigen processing and presentation of exogenous protein antigen via MHC class Ib, TAP-dependent///drug transport///establishment of endothelial blood-brain barrier///positive regulation of antigen processing and presentation of peptide antigen via MHC class I///response to drug///transmembrane transport///transport | Golgi membrane///apical plasma membrane///integral component of membrane///intercellular canaliculus///membrane///mitochondrion///plasma membrane |
| 1447851_x_at | 0.000936 | 3.86E-06 | 1.20E+01 | 5.111678 | 2.88 | Atp10a | ATPase, class V, type 10A | ATP binding///hydrolase activity///magnesium ion binding///metal ion binding///nucleotide binding///phospholipid-translocating ATPase activity | lipid transport///phospholipid transport///transport | endoplasmic reticulum///integral component of membrane///membrane///plasma membrane |
| 1457634_at | 0.000958 | 3.99E-06 | -1.20E+01 | 5.080127 | -4.4 | Tmsb15b2///Tmsb15b1///Tmsb15l | thymosin beta 15b2///thymosin beta 15b1///thymosin beta 15b like | actin monomer binding///molecular_function///actin monomer binding | negative regulation of actin filament polymerization///negative regulation of stress fiber assembly///sequestering of actin monomers///biological_process///negative regulation of actin filament polymerization///negative regulation of stress fiber assembly///sequestering of actin monomers | filamentous actin///stress fiber///cellular_component///filamentous actin///stress fiber |
| 1447927_at | 0.000958 | 3.99E-06 | 1.20E+01 | 5.079209 | 5.7 | Gbp6 | guanylate binding protein 6 | molecular_function | adhesion of symbiont to host///cellular response to interferon-beta///cellular response to interferon-gamma///cellular response to lipopolysaccharide///defense response to Gram-positive bacterium///defense response to Gram-positive bacterium///defense response to protozoan | cytoplasmic vesicle///extracellular exosome///symbiont-containing vacuole membrane |
| 1429947_a_at | 0.00096 | 4.02E-06 | 1.19E+01 | 5.072076 | 4.83 | Zbp1 | Z-DNA binding protein 1 | DNA binding///DNA binding///RNA binding///double-stranded RNA adenosine deaminase activity///left-handed Z-DNA binding | defense response to virus///immune system process///innate immune response///positive regulation of type I interferon-mediated signaling pathway///viral process | cytoplasm///cytosol///nucleus |
| 1459900_at | 0.000966 | 4.07E-06 | -1.19E+01 | 5.060782 | -2.75 | C79468 | expressed sequence C79468 | | | |
| 1417292_at | 0.000966 | 4.09E-06 | 1.19E+01 | 5.055962 | 3.46 | Ifi47 | interferon gamma inducible protein 47 | | defense response | endoplasmic reticulum |
| 1423414_at | 0.00097 | 4.14E-06 | -1.19E+01 | 5.044082 | -5.13 | Ptgs1 | prostaglandin-endoperoxide synthase 1 | dioxygenase activity///heme binding///lipid binding///metal ion binding///oxidoreductase activity///peroxidase activity///prostaglandin-endoperoxide synthase activity | cyclooxygenase pathway///fatty acid biosynthetic process///fatty acid metabolic process///inflammatory response///keratinocyte differentiation///learning///lipid metabolic process///maintenance of blood-brain barrier///memory///negative regulation of epinephrine secretion///negative regulation of norepinephrine secretion///oxidation-reduction process///positive regulation of smooth muscle contraction///positive regulation of vasoconstriction///prostaglandin biosynthetic process///prostaglandin biosynthetic process///prostaglandin metabolic process///regulation of blood pressure///regulation of cell proliferation///response to oxidative stress///sensory perception of pain | Golgi apparatus///cytoplasm///cytoplasm///endoplasmic reticulum///extracellular exosome///intracellular membrane-bounded organelle///membrane///nuclear envelope///NOT nucleus///photoreceptor outer segment |
| 1455015_at | 0.00097 | 4.15E-06 | 1.19E+01 | 5.041168 | 2.66 | Tbc1d9 | TBC1 domain family, member 9 | GTPase activator activity///Rab GTPase binding///calcium ion binding | activation of GTPase activity///intracellular protein transport///regulation of vesicle fusion | endomembrane system///intracellular |
| 1419647_a_at | 0.000987 | 4.25E-06 | 1.19E+01 | 5.019302 | 2.25 | Ier3 | immediate early response 3 | protein binding | intrinsic apoptotic signaling pathway in response to DNA damage///mitotic G2 DNA damage checkpoint///negative regulation of apoptotic process///negative regulation of glycolytic process///negative regulation of inflammatory response///negative regulation of mitochondrial outer membrane permeabilization involved in apoptotic signaling pathway///negative regulation of systemic arterial blood pressure///positive regulation of protein catabolic process///regulation of DNA repair///regulation of nucleocytoplasmic transport///regulation of reactive oxygen species metabolic process///regulation of response to DNA damage stimulus///regulation of response to DNA damage stimulus///response to protozoan | integral component of membrane///membrane///nucleus///nucleus |
| 1442134_at | 0.00099 | 4.28E-06 | -1.18E+01 | 5.01145 | -4.33 | Prr11 | proline rich 11 | molecular_function | regulation of cell cycle | cytoplasm///membrane///nucleus |
| 1437760_at | 0.000998 | 4.35E-06 | -1.18E+01 | 4.996277 | -2.88 | Galnt12 | UDP-N-acetyl-alpha-D-galactosamine:polypeptide N-acetylgalactosaminyltransferase 12 | 1,2-dihydroxy-phenanthrene glycosyltransferase activity///1-phenanthrol glycosyltransferase activity///9-phenanthrol UDP-glucuronosyltransferase activity///9-phenanthrol glycosyltransferase activity///O antigen polymerase activity///UDP-glucose:glycoprotein glucosyltransferase activity///acetylgalactosaminyltransferase activity///acetylglucosaminyltransferase activity///alpha-(1->3)-fucosyltransferase activity///alpha-(1->6)-fucosyltransferase activity///alpha-1,2-galactosyltransferase activity///alpha-1,2-mannosyltransferase activity///alpha-1,3-galactosyltransferase activity///alpha-1,3-mannosyltransferase activity///alpha-1,6-mannosyltransferase activity///benzoic acid glucosyltransferase activity///beta-1,4-mannosyltransferase activity///carbohydrate binding///cellulose synthase activity///chondroitin hydrolase activity///cytokinin 9-beta-glucosyltransferase activity///dolichyl pyrophosphate Glc1Man9GlcNAc2 alpha-1,3-glucosyltransferase activity///dolichyl pyrophosphate Man7GlcNAc2 alpha-1,3-glucosyltransferase activity///dolichyl pyrophosphate Man9GlcNAc2 alpha-1,3-glucosyltransferase activity///dolichyl-phosphate-glucose-glycolipid alpha-glucosyltransferase activity///dolichyl-pyrophosphate Man7GlcNAc2 alpha-1,6-mannosyltransferase activity///fucosyltransferase activity///galactosyltransferase activity///glucosyltransferase activity///glycolipid mannosyltransferase activity///indole-3-butyrate beta-glucosyltransferase activity///inositol phosphoceramide synthase activity///lipopolysaccharide-1,5-galactosyltransferase activity///lipopolysaccharide-1,6-galactosyltransferase activity///mannosyltransferase activity///metal ion binding///molecular_function///oligosaccharyl transferase activity///phenanthrol glycosyltransferase activity///polypeptide N-acetylgalactosaminyltransferase activity///salicylic acid glucosyltransferase (ester-forming) activity///salicylic acid glucosyltransferase (glucoside-forming) activity///transferase activity///transferase activity, transferring glycosyl groups | biological_process | Golgi apparatus///cellular_component///integral component of membrane///membrane |
| 1458432_at | 0.000998 | 4.38E-06 | -1.18E+01 | 4.989325 | -3.37 |  |  |  |  |  |
| 1457293_at | 0.000998 | 4.39E-06 | -1.18E+01 | 4.987613 | -2.33 | Zbtb4 | zinc finger and BTB domain containing 4 | DNA binding///RNA polymerase II regulatory region sequence-specific DNA binding///metal ion binding///methyl-CpG binding///methyl-CpNpG binding///nucleic acid binding///protein homodimerization activity///protein kinase binding///sequence-specific DNA binding///transcriptional repressor activity, RNA polymerase II transcription regulatory region sequence-specific binding | cellular response to DNA damage stimulus///negative regulation of transcription from RNA polymerase II promoter///negative regulation of transcription, DNA-templated///regulation of transcription, DNA-templated///transcription, DNA-templated | chromosome///nucleus |
| 1419045_at | 0.000998 | 4.43E-06 | -1.18E+01 | 4.977996 | -2.01 | Slc25a23 | solute carrier family 25 (mitochondrial carrier; phosphate carrier), member 23 | calcium ion binding///metal ion binding///structural constituent of ribosome | adenine nucleotide transport///calcium ion transmembrane import into mitochondrion///cellular response to calcium ion///mitochondrial calcium ion transport///regulation of cellular respiration///regulation of oxidative phosphorylation///regulation of sequestering of calcium ion///translation///transmembrane transport///transport///urea homeostasis | integral component of membrane///membrane///mitochondrial inner membrane///mitochondrion |
