# Supplementary figures and images for: Down-regulated GAS6 impairs synovial macrophage efferocytosis and promotes obesity-associated osteoarthritis
Source: eLife. 2023 May 5;12:e83069. doi: 10.7554/eLife.83069 (PMC10191622; doi:10.7554/eLife.83069)

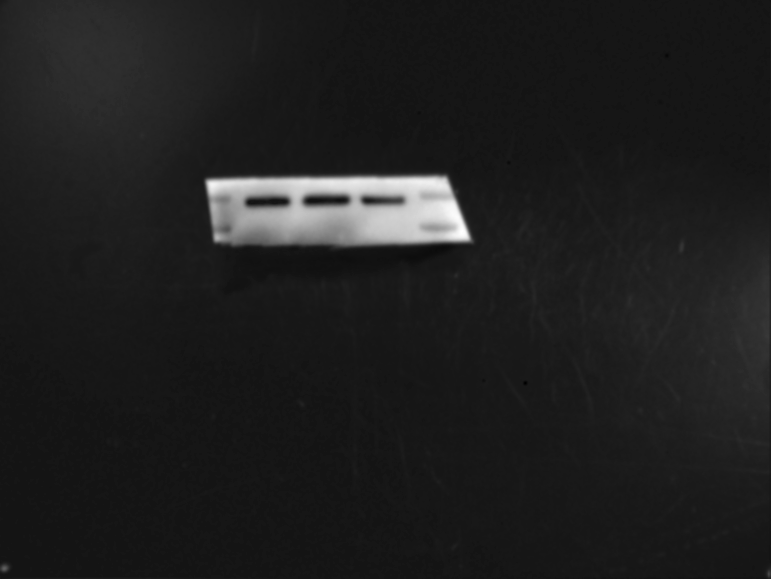

Supplement: Figure 3—figure supplement 2—source data 1. [file elife-83069-fig3-figsupp2-data1.zip › Figure 3- figure supplement 2 primary blots/CD86-BAND.tif]

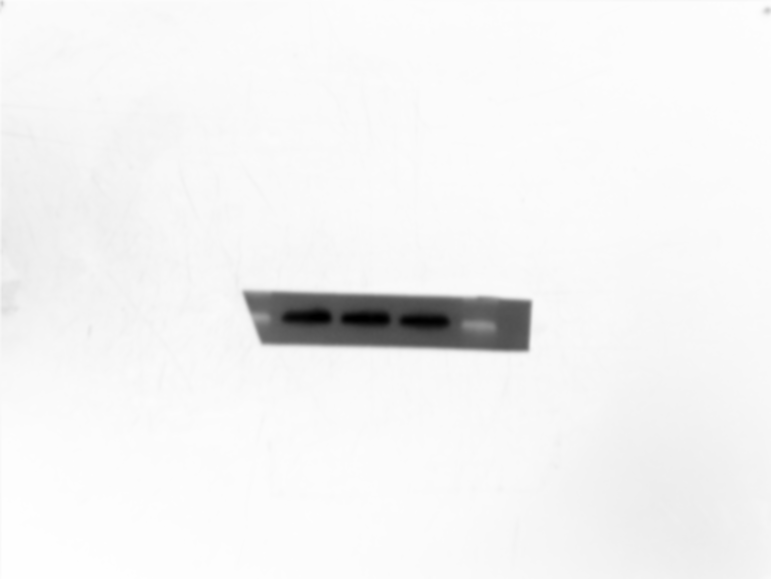

Supplement: Figure 3—figure supplement 2—source data 1. [file elife-83069-fig3-figsupp2-data1.zip › Figure 3- figure supplement 2 primary blots/GAPDH-band.tif]

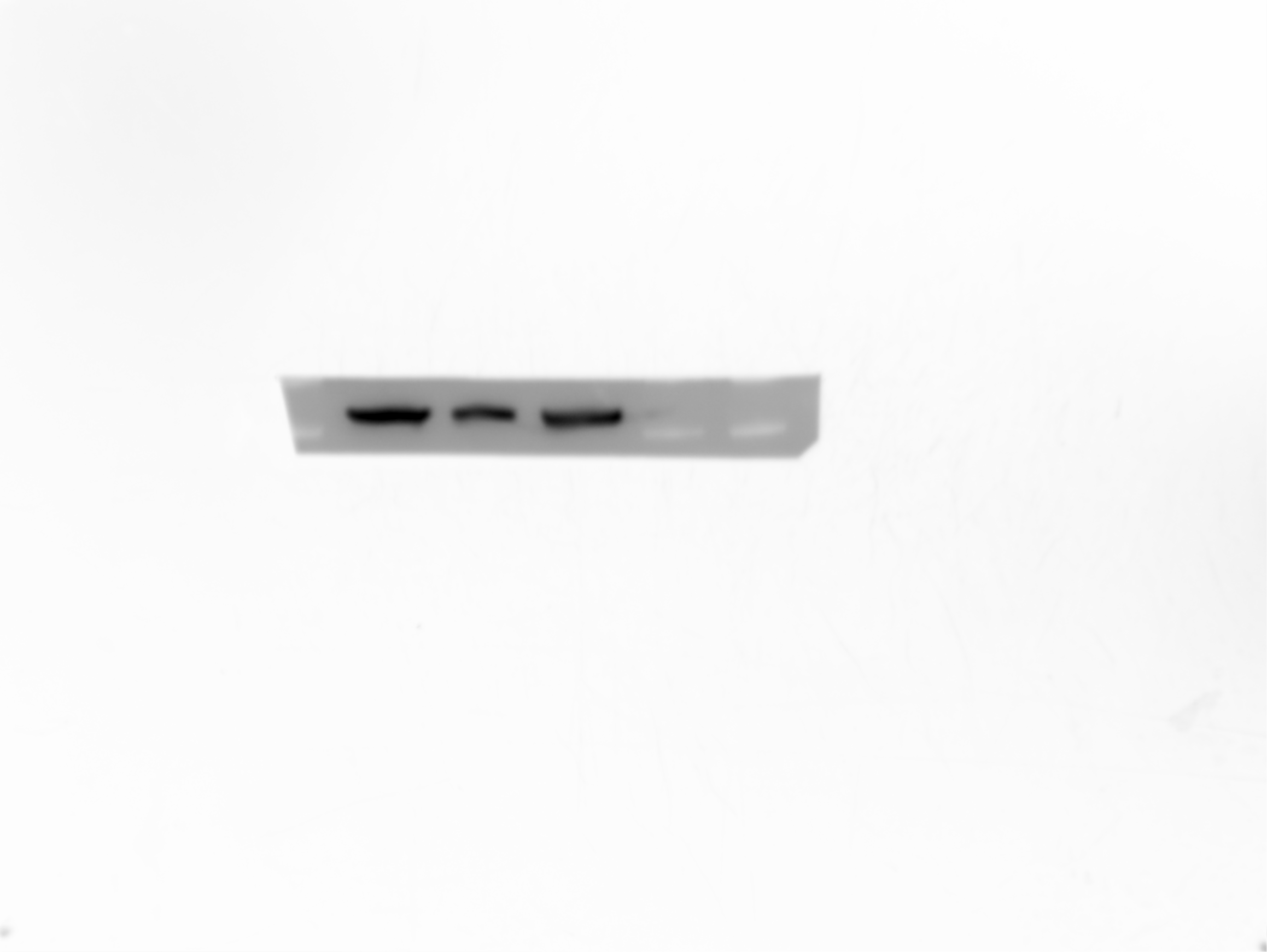

Supplement: Figure 3—figure supplement 2—source data 1. [file elife-83069-fig3-figsupp2-data1.zip › Figure 3- figure supplement 2 primary blots/GAS6-BAND.tif]

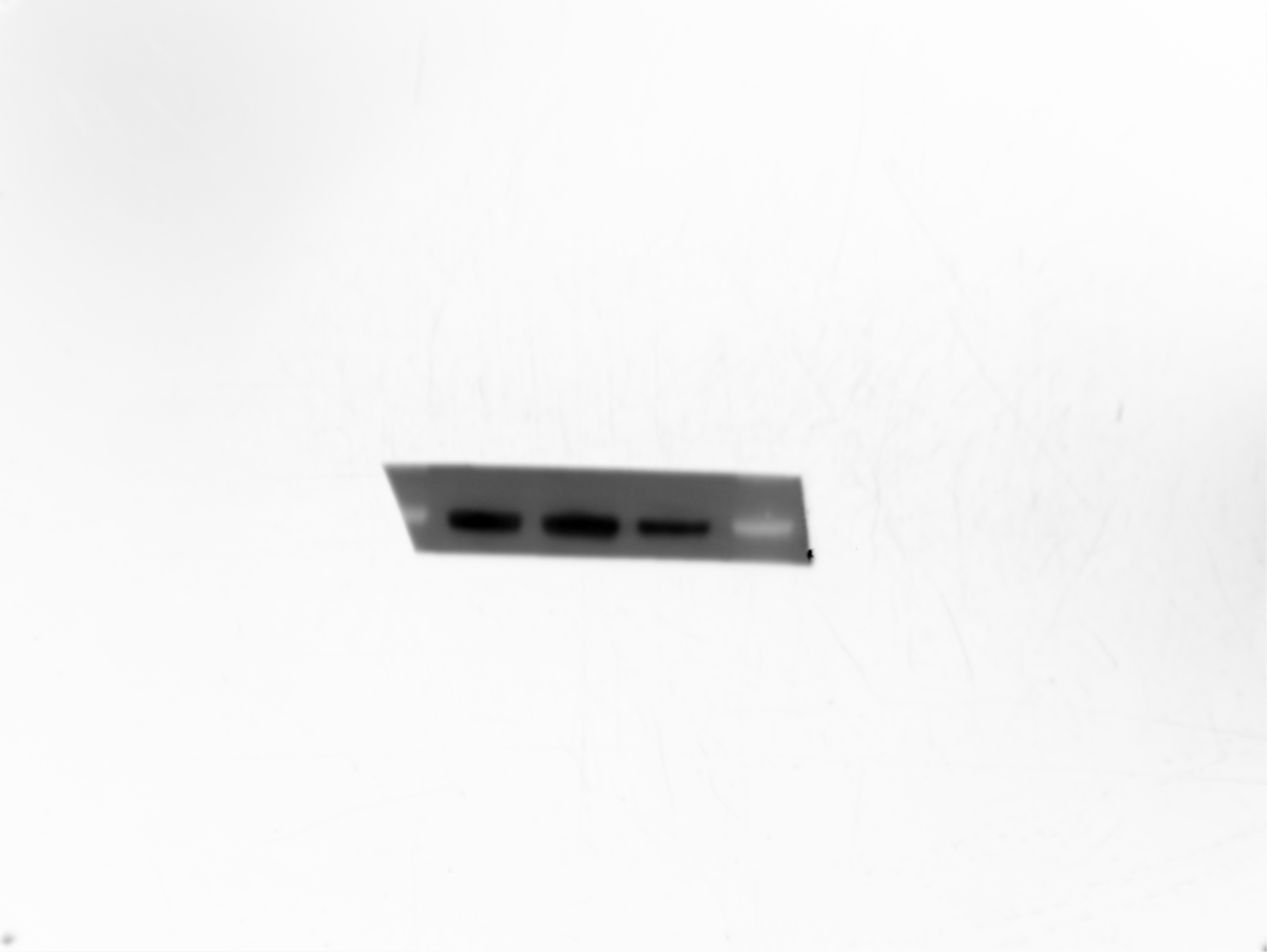

Supplement: Figure 3—figure supplement 2—source data 1. [file elife-83069-fig3-figsupp2-data1.zip › Figure 3- figure supplement 2 primary blots/nos2-band.tif]

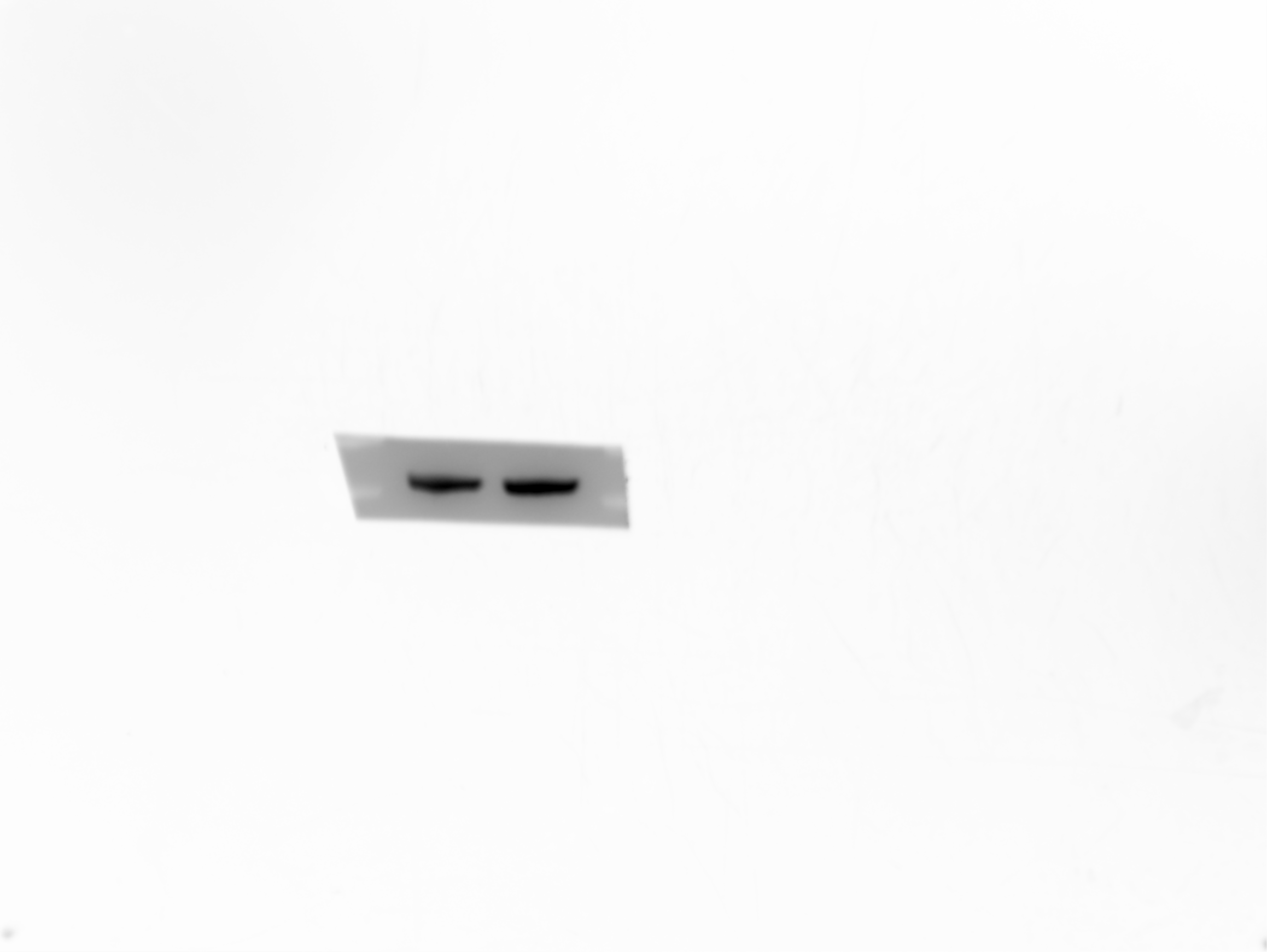

Supplement: Figure 4—figure supplement 3—source data 1. [file elife-83069-fig4-figsupp3-data1.zip › Figure 4-figure supplement 3 primary blots/CD86.tif]

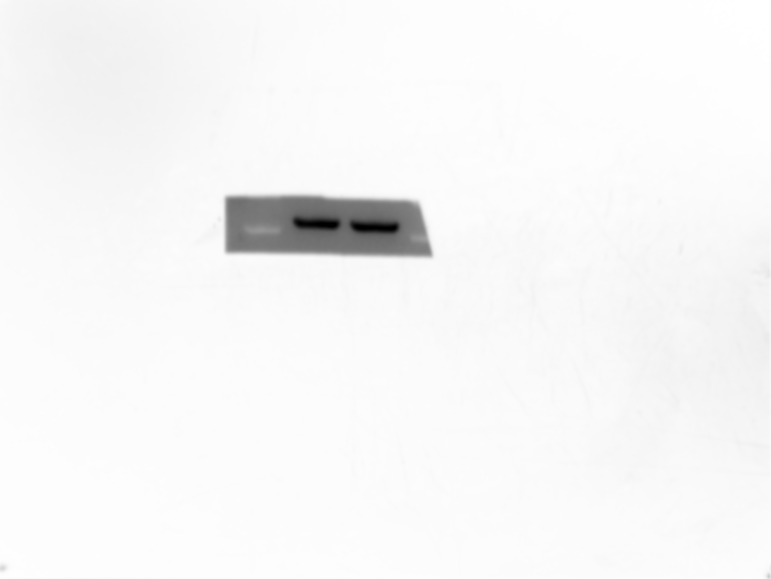

Supplement: Figure 4—figure supplement 3—source data 1. [file elife-83069-fig4-figsupp3-data1.zip › Figure 4-figure supplement 3 primary blots/GAPDH.tif]

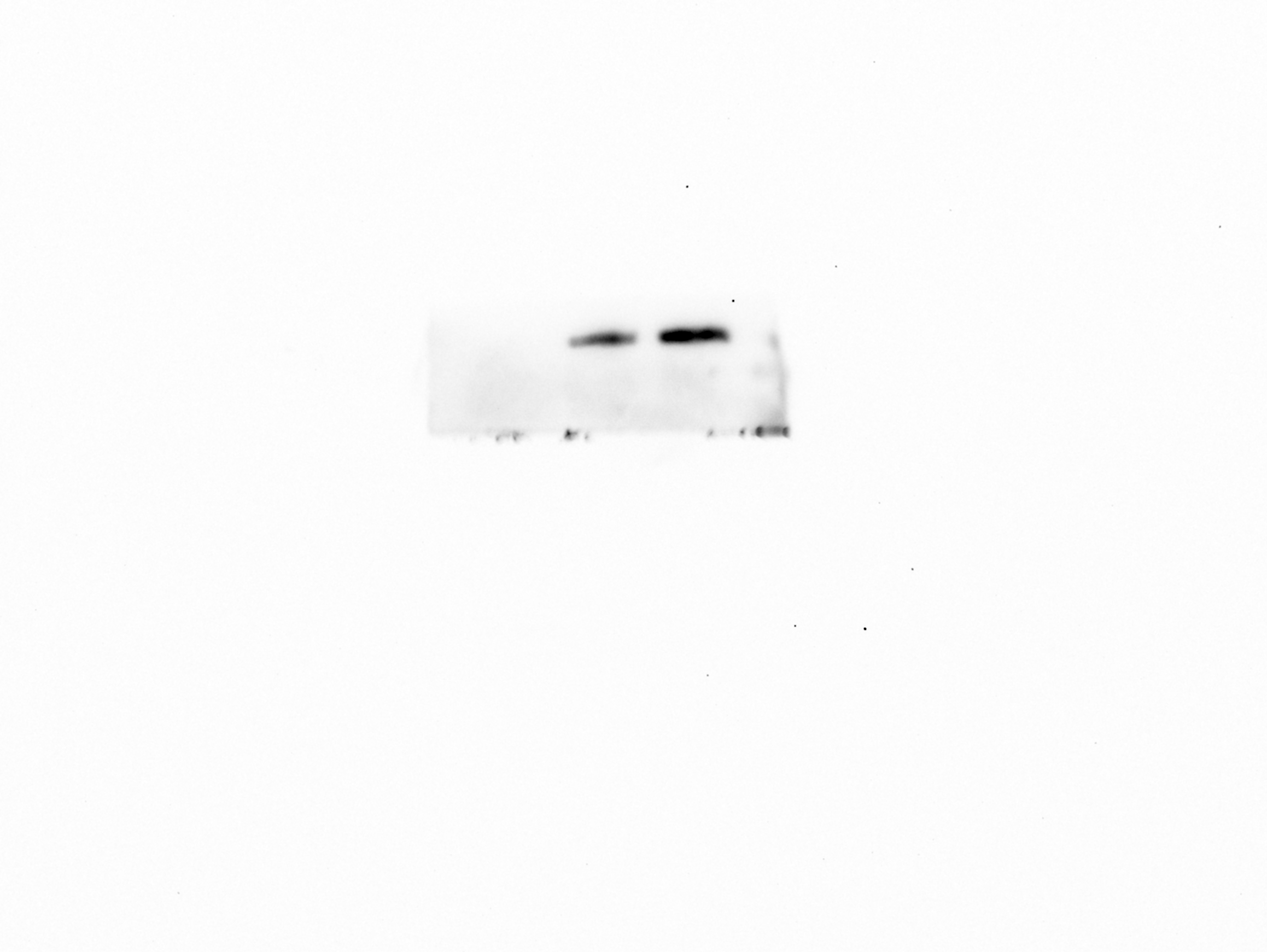

Supplement: Figure 4—figure supplement 3—source data 1. [file elife-83069-fig4-figsupp3-data1.zip › Figure 4-figure supplement 3 primary blots/iNOS.tif]

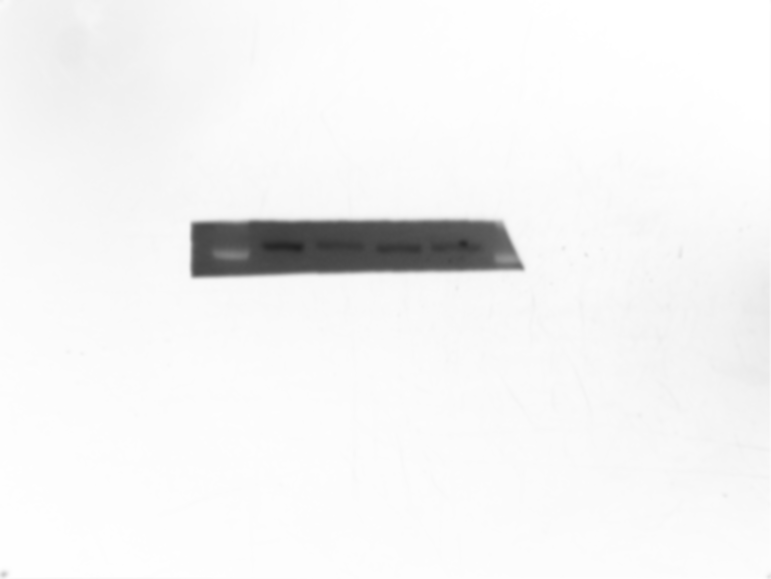

Supplement: Figure 5—figure supplement 1—source data 1. [file elife-83069-fig5-figsupp1-data1.zip › Figure 5-Figure supplement 1 primary blots/COL2.tif]

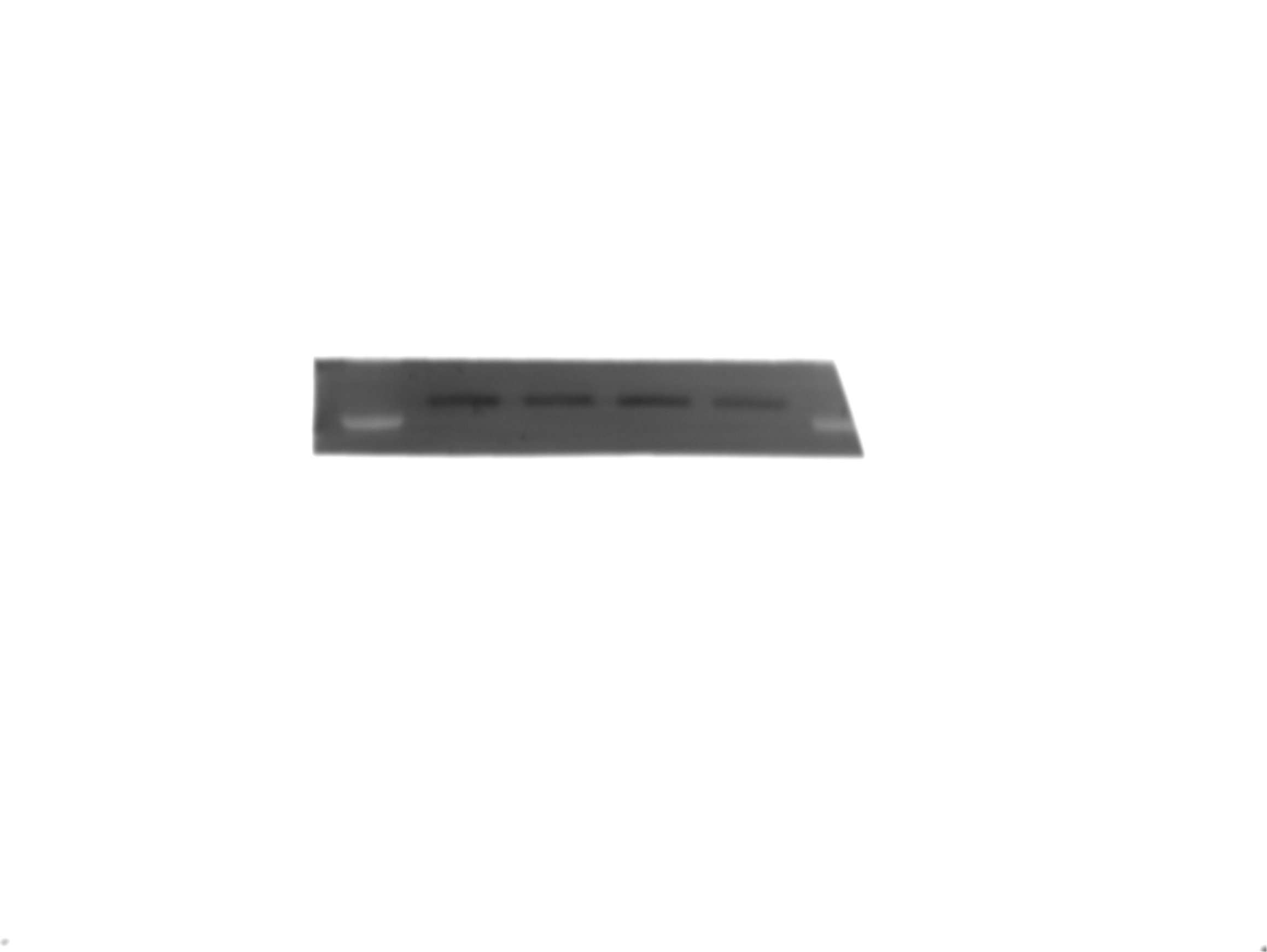

Supplement: Figure 5—figure supplement 1—source data 1. [file elife-83069-fig5-figsupp1-data1.zip › Figure 5-Figure supplement 1 primary blots/GAPDH.tif]

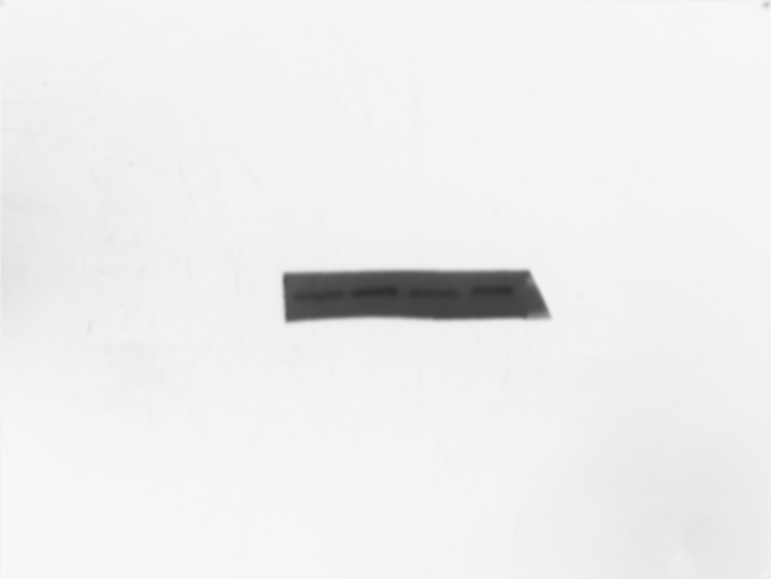

Supplement: Figure 5—figure supplement 1—source data 1. [file elife-83069-fig5-figsupp1-data1.zip › Figure 5-Figure supplement 1 primary blots/MMP13.tif]

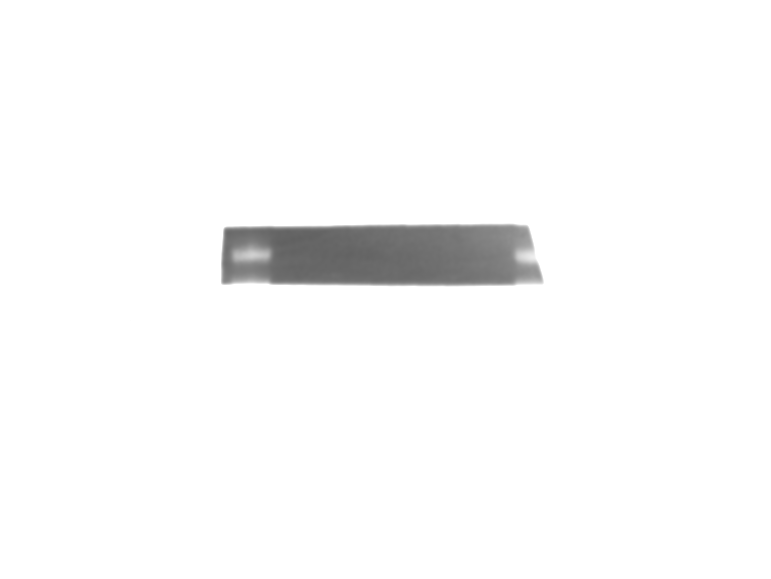

Supplement: Figure 5—figure supplement 1—source data 1. [file elife-83069-fig5-figsupp1-data1.zip › Figure 5-Figure supplement 1 primary blots/p16.tif]

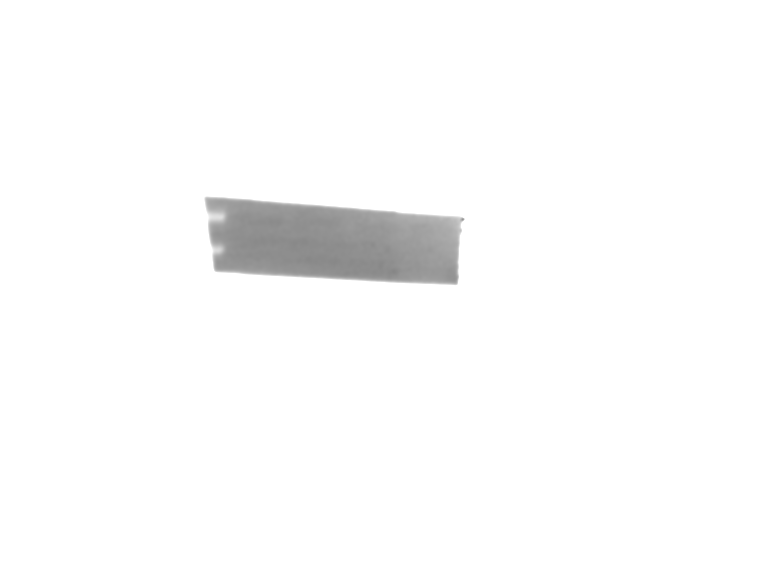

Supplement: Figure 5—figure supplement 1—source data 1. [file elife-83069-fig5-figsupp1-data1.zip › Figure 5-Figure supplement 1 primary blots/p21.tif]

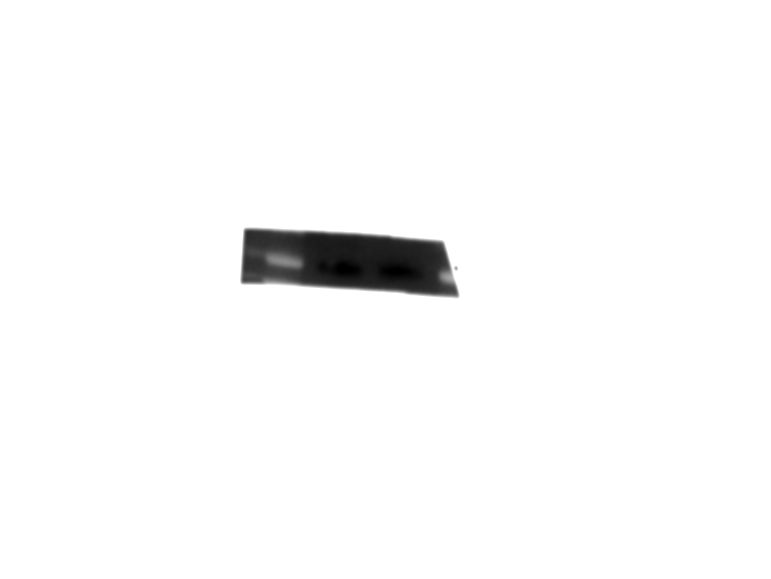

Supplement: Figure 5—figure supplement 3—source data 1. [file elife-83069-fig5-figsupp3-data1.zip › Figure 5-Figure supplement 3 primary blots/col2-band - 副本.tif]

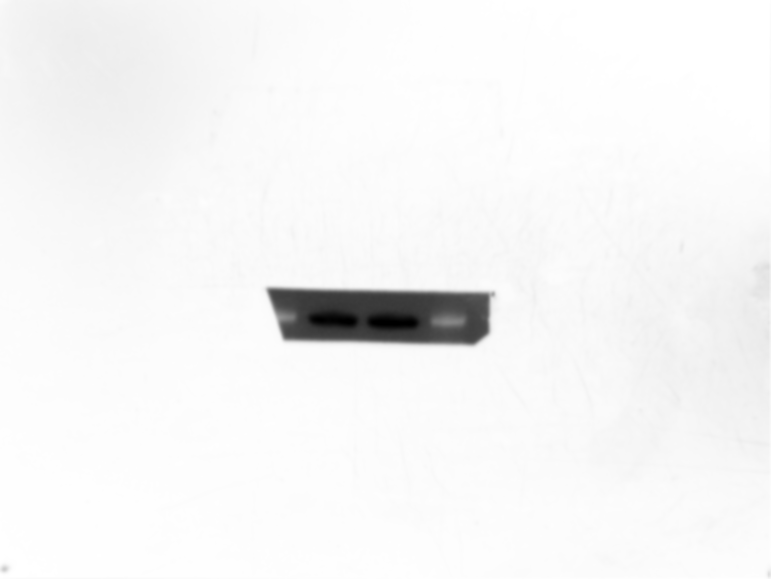

Supplement: Figure 5—figure supplement 3—source data 1. [file elife-83069-fig5-figsupp3-data1.zip › Figure 5-Figure supplement 3 primary blots/gapdh-band.tif]

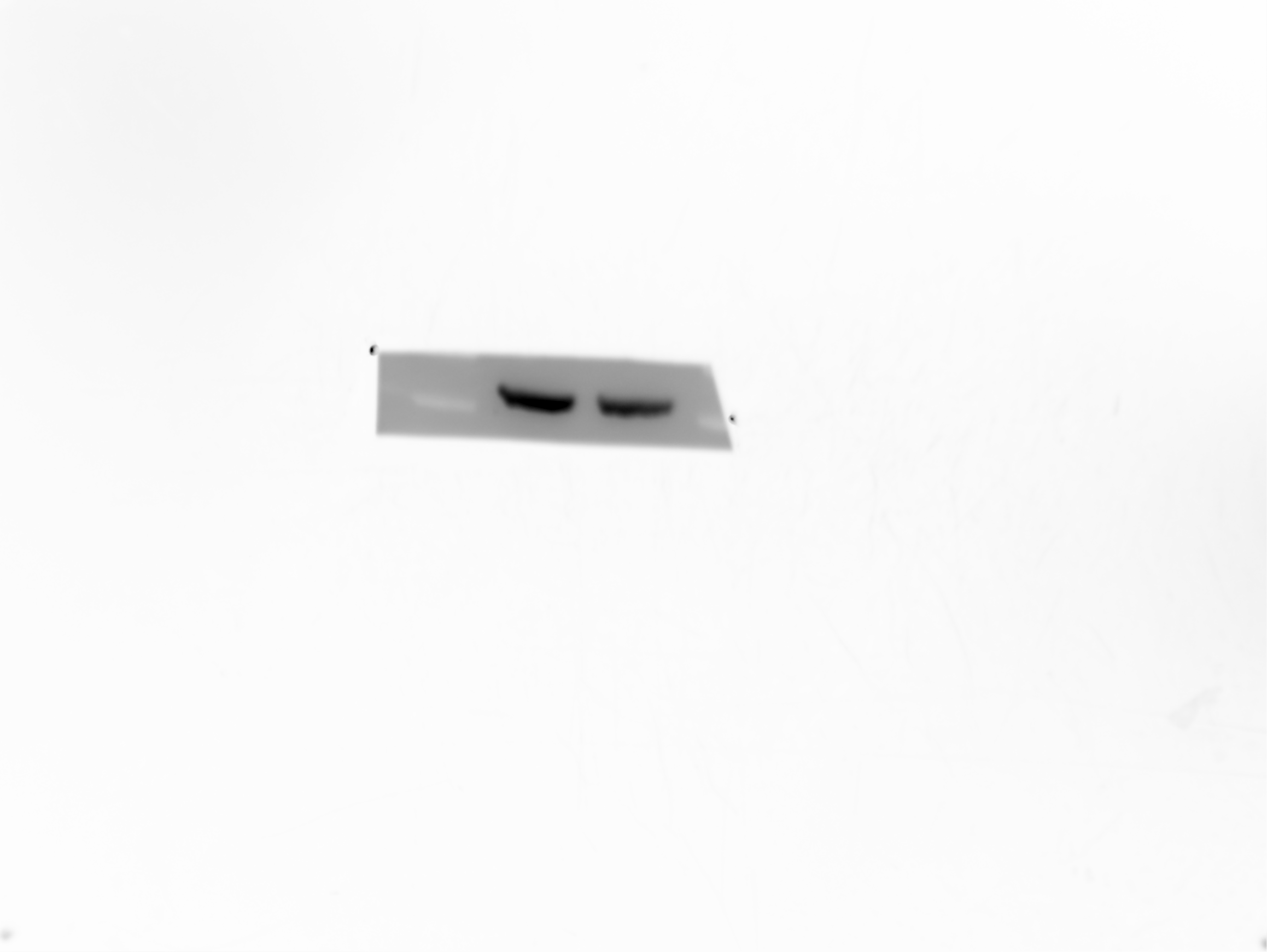

Supplement: Figure 5—figure supplement 3—source data 1. [file elife-83069-fig5-figsupp3-data1.zip › Figure 5-Figure supplement 3 primary blots/mmp13-band.tif]

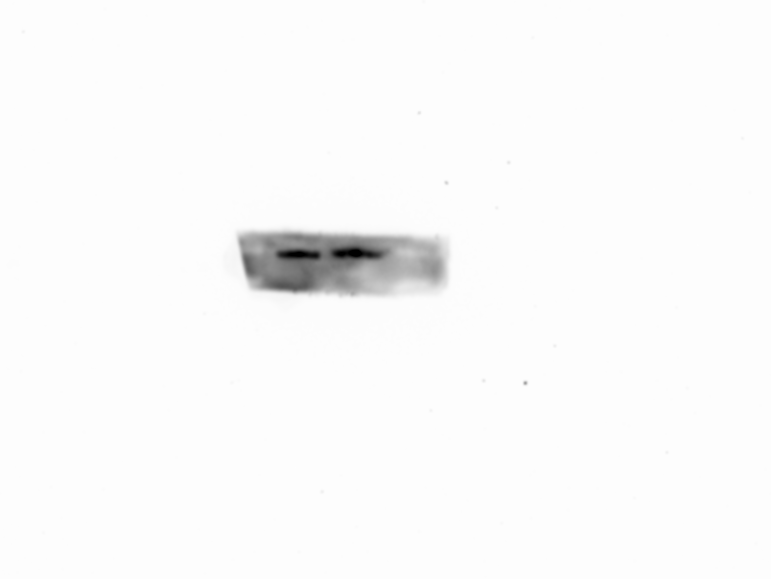

Supplement: Figure 5—figure supplement 3—source data 1. [file elife-83069-fig5-figsupp3-data1.zip › Figure 5-Figure supplement 3 primary blots/p16-band.tif]

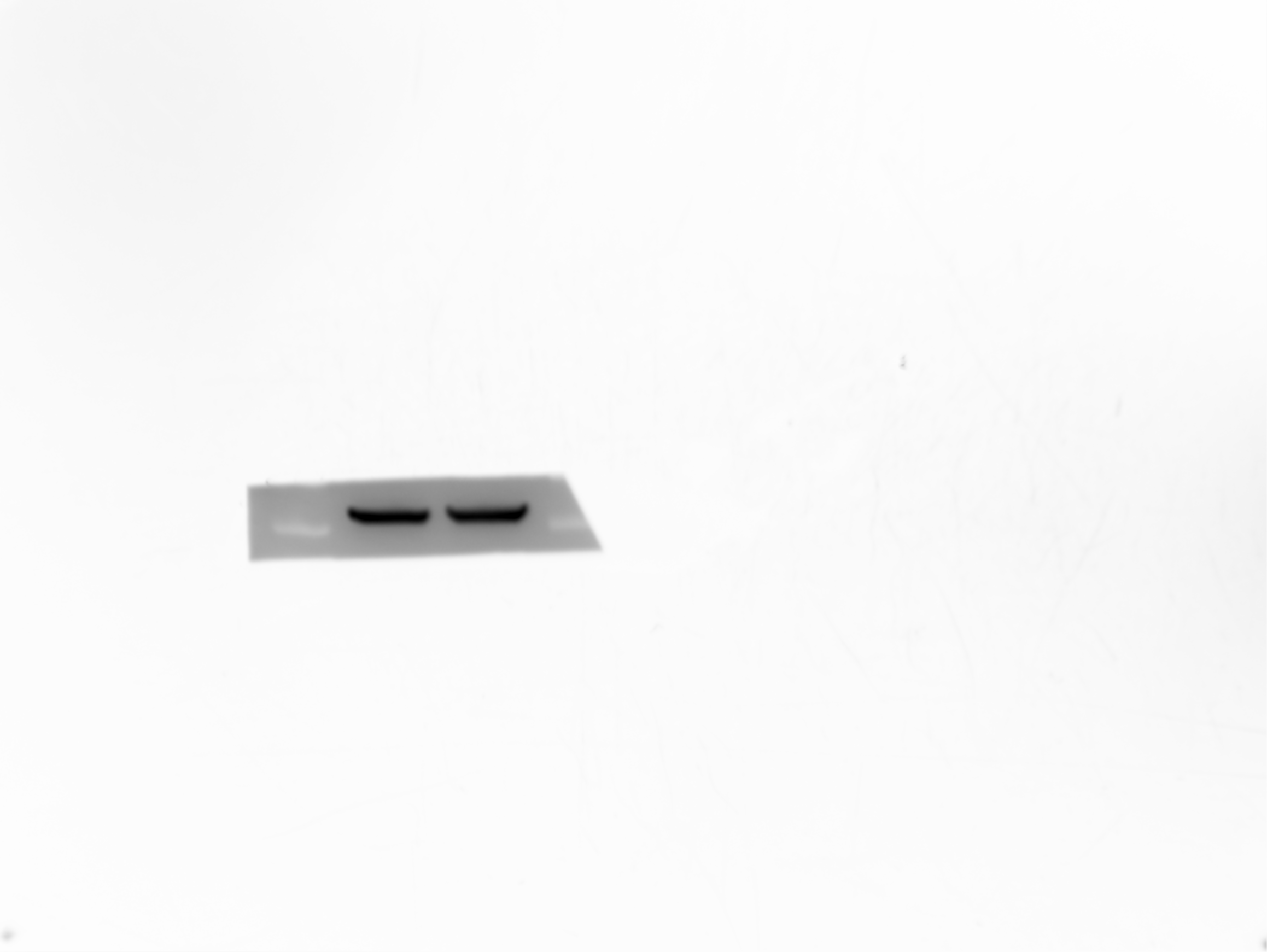

Supplement: Figure 5—figure supplement 3—source data 1. [file elife-83069-fig5-figsupp3-data1.zip › Figure 5-Figure supplement 3 primary blots/p21-band.tif]
